# Biased, Non-equivalent Gene-Proximal and -Distal Binding Motifs of Orphan Nuclear Receptor TR4 in Primary Human Erythroid Cells

**DOI:** 10.1371/journal.pgen.1004339

**Published:** 2014-05-08

**Authors:** Lihong Shi, M. C. Sierant, Katherine Gurdziel, Fan Zhu, Shuaiying Cui, Katarzyna E. Kolodziej, John Strouboulis, Yuanfang Guan, Osamu Tanabe, Kim-Chew Lim, James Douglas Engel

**Affiliations:** 1 Department of Cell and Developmental Biology, University of Michigan Medical School, Ann Arbor, Michigan, United States of America; 2 Department of Computational Medicine and Bioinformatics, University of Michigan Medical School, Ann Arbor, Michigan, United States of America; 3 PharmaMatch, Amsterdam, Netherlands; 4 Institute of Molecular Oncology, BSRC Alexander Fleming, Varkiza, Greece; 5 Department of Integrative Genomics, Tohoku Medical Megabank, Tohoku University, Sendai, Japan; Stanford University School of Medicine, United States of America

## Abstract

We previously reported that TR2 and TR4 orphan nuclear receptors bind to direct repeat (DR) elements in the ε- and γ-globin promoters, and act as molecular anchors for the recruitment of epigenetic corepressors of the multifaceted DRED complex, thereby leading to ε- and γ-globin transcriptional repression during definitive erythropoiesis. Other than the ε- and γ-globin and the *GATA1* genes, TR4-regulated target genes in human erythroid cells remain unknown. Here, we identified TR4 binding sites genome-wide using chromatin immunoprecipitation followed by massively parallel sequencing (ChIP-seq) as human primary CD34^+^ hematopoietic progenitors differentiated progressively to late erythroid precursors. We also performed whole transcriptome analyses by RNA-seq to identify TR4 downstream targets after lentiviral-mediated TR4 shRNA knockdown in erythroid cells. Analyses from combined ChIP-seq and RNA-seq datasets indicate that DR1 motifs are more prevalent in the proximal promoters of TR4 direct target genes, which are involved in basic biological functions (e.g., mRNA processing, ribosomal assembly, RNA splicing and primary metabolic processes). In contrast, other non-DR1 repeat motifs (DR4, ER6 and IR1) are more prevalent at gene-distal TR4 binding sites. Of these, approximately 50% are also marked with epigenetic chromatin signatures (such as P300, H3K27ac, H3K4me1 and H3K27me3) associated with enhancer function. Thus, we hypothesize that TR4 regulates gene transcription *via* gene-proximal DR1 sites as TR4/TR2 heterodimers, while it can associate with novel nuclear receptor partners (such as RXR) to bind to distant non-DR1 consensus sites. In summary, this study reveals that the TR4 regulatory network is far more complex than previously appreciated and that TR4 regulates basic, essential biological processes during the terminal differentiation of human erythroid cells.

## Author Summary

Sequential genome-wide binding studies investigated by deep sequencing (ChIP-seq) represent a powerful tool for investigating the temporal sequence of gene activation and repression events that take place as cells differentiate. Here, we report the binding of an “orphan” nuclear receptor (one for which no ligand has been identified) to its cognate genomic regulatory sites and perform the functional analysis to validate its downstream targets as precursor cells differentiate from very early human hematopoietic progenitors into red blood cells. We discovered that when this receptor is bound at gene proximal promoters, it recognizes a different DNA sequence than when it binds to more distant regulatory sites (enhancers and silencers). Since this receptor can either activate or repress specific target genes, the data suggest the intriguing possibility that the two different modes of DNA recognition may reflect association of the receptor with different partner molecules when regulating gene expression from proximal or distal sequences.

## Introduction

Sickle cell disease (SCD) is an inherited blood disorder caused by a missense mutation in the adult β-globin gene and affects 70,000–90,000 people in the United States and millions worldwide [1], [2]. Patients bearing Hereditary Persistence of Fetal Hemoglobin (HPFH) mutations have high levels of fetal γ-hemoglobin (HbF) that aberrantly persist into adulthood [3]. It has been observed clinically that the co-inheritance of an HPFH mutation in SCD patients significantly mitigates SCD symptoms [4]–[7]. Thus, for the past three decades, the scientific community has made concerted efforts to develop strategies by which we might safely and efficiently induce HbF synthesis in adult definitive erythroid cells as a potential therapy to treat SCD.

We previously identified an essential direct repeat 1 (DR1) *cis*-element, a consensus binding site for non-steroidal nuclear receptors [8], in the ε- and γ-globin proximal promoters [9]. We further experimentally demonstrated that these DR1 elements are critical for ε- and γ-globin gene silencing in adult erythrocytes [9]; consistent with this finding, the adult β-globin gene contains no such element. Using adult (definitive) murine erythroleukemia (MEL) nuclear cell extracts, we identified the orphan nuclear receptors TR2 (NR2C1) and TR4 (NR2C2) that as a heterodimer was capable of preferential high-affinity binding to the ε- and γ-globin DR1 elements and of recruiting chromatin modifying cofactors to those binding sites [10], [11]. We further showed in compound TR2^−/−^/TR4^−/−^ mutant embryos bearing a human β-globin YAC transgene that ε- and γ-globin gene expression was elevated in definitive erythrocytes [12]. Hence, the TR2/TR4 heterodimer mediates ε- and γ-globin gene silencing through essential DR1 elements in the ε- and γ-globin promoters in definitive red cells. We reasoned that TR4 might therefore be a potential molecule to target for developing interventional therapies for SCD. Before that, however, it would be important to identify the array of TR4 target genes and the physiological functions they participate in during human erythroid differentiation.

Compared with TR2, TR4 appears to play a more prominent role in γ-globin repression during definitive erythropoiesis [12], and therefore we focused on TR4 in this study. Although TR4 was initially isolated from human prostate and testis cDNA libraries using sequence homology to other known nuclear receptors [13], it was subsequently shown to be ubiquitously expressed [14]. Although TR4 is categorized as an orphan nuclear receptor, it appears structurally to be able to accommodate a small molecule effector in the position of the usual ligand-binding pocket [15]. TR4 can form either homodimers or heterodimers with TR2, androgen receptor (AR) [16], or estrogen receptor (ER) [17]. Both *in vitro* and *in vivo* studies have shown that TR4 is capable of binding to an imperfect DR consensus sequence separated by zero to five spacer nucleotides (DR0 - DR5) [9], [18]–[20]. When TR4 binds to promoter DR elements, it is reportedly able to act as either an activator or a repressor [21]. Besides developmental regulation of the globin switch [12], TR4 has also been shown to play prominent roles in muscle, neuronal and bone development, as well as in spermatogenesis and lipid/lipoprotein metabolism [20], [22]–[24].

Prior to the present study, genome-wide TR4 binding site analysis was performed in four ENCODE cell lines: K562 embryonic erythroleukemia cells, HepG2 liver carcinoma cells, HeLa cervical carcinoma cells and GM12878 immortalized lymphoblast cells [25]. However, in primary erythroid cells, in addition to the ε- and γ-globin genes, the only other known TR4 target gene was *GATA1*, which is stage-specifically bound by TR4 at a DR1 element located within the *GATA1* hematopoietic enhancer (*G1HE*) [26], [27]. Thus, a comprehensive analysis of the putative targets and physiological functions of TR4 in human primary erythroid cells has not been previously investigated.

In this study, we resolved the genome-wide binding of TR4 in differentiating human erythroid cells by performing chromatin immunoprecipitation followed by next-generation sequencing (ChIP-seq). We also performed whole transcriptome analyses by RNA-seq to identify genes that are regulated by TR4 both before and after lentiviral-mediated TR4 shRNA knockdown in erythroid cells. We found that TR4 preferentially binds to DR1 elements in the promoters of such direct target genes, and that the majority of these genes encode proteins that participate in fundamental biological functions such as mRNA processing, translation, RNA splicing and primary metabolic processes. Interestingly, we also found an increased occurrence of non-DR1 repeat elements (such as DR4, IR1 and ER6) bound by TR4 at sites located more than 10 Kbp away from the nearest gene. This raised the tantalizing possibility that TR4 might heterodimerize with nuclear receptors other than TR2, thereby allowing TR4 to elicit unique transcriptional responses when acting at proximal (promoter) and distal (enhancer and silencer) regulatory sites during human erythropoiesis. In support of this hypothesis, we detected a specific TR4/RXR interaction in MEL cells in which biotin-tagged TR4 was forcibly expressed. In summary, these combined ChIP-seq and RNA-seq analyses provide a genome-wide map of TR4 binding sites as well as a comprehensive list of *bona fide* TR4-regulated downstream target genes during human erythroid differentiation *ex vivo*.

## Results

### Identification of genome-wide TR4 binding in differentiating human erythroid cells

Primary human CD34^+^ hematopoietic progenitor cells were cultured *ex vivo* using a previously described two-phase erythroid differentiation system [10], [11]. At culture day 8 (D8), proerythroblasts were the predominant cell population [10]. By day 11 (D11), most cells were at an intermediate differentiation stage that resembled polychromatic and orthochromatic erythroblasts. Finally by day 14 (D14) of culture, the cells had matured into reticulocytes with approximately 30% of the cells having undergone enucleation [10], [11]. From D8 to D14, endogenous TR4 accumulated more abundantly with maturation (**Figure S1**). D8, D11 and D14 cells were separately harvested for ChIP-seq and RNA-seq assays.

We then performed TR4 ChIP-seq on D8, D11 and D14 cells from two independent differentiating CD34^+^ cell cultures and generated a total of 32, 54 and 55×10^6^ TR4 ChIP-enriched, 50-bp short reads, respectively (**Table 1**). By applying a statistical cutoff of *p*<10^−5^, we identified 1,025, 375 and 323 TR4 peaks on D8, D11 and D14, respectively (**Table 1**). As a representative example, robust TR4 binding to the DR1 site in the promoter of *CHMP4B* was detected throughout differentiation (**Figure S2**). To further assess the quality of the ChIP-seq data, thirty peaks detected at all three differentiation stages were further evaluated by ChIP-qPCR, and a strong correlation (0.82) between the two independent assays was observed for those peaks (**Figure S3A**).

**Table 1:** Total raw reads and peaks from TR4 ChIP-seq analyses of differentiating human erythroid cells.

Differentiation day		D8	D11	D14
**Raw Reads**	Input	38,295,288	49,304,282	54,360,891
	TR4	32,499,078	54,074,411	55,628,112
**Peaks (** ***p*** **<10^−5^)**		1,025	375	323

**Figure 1:**
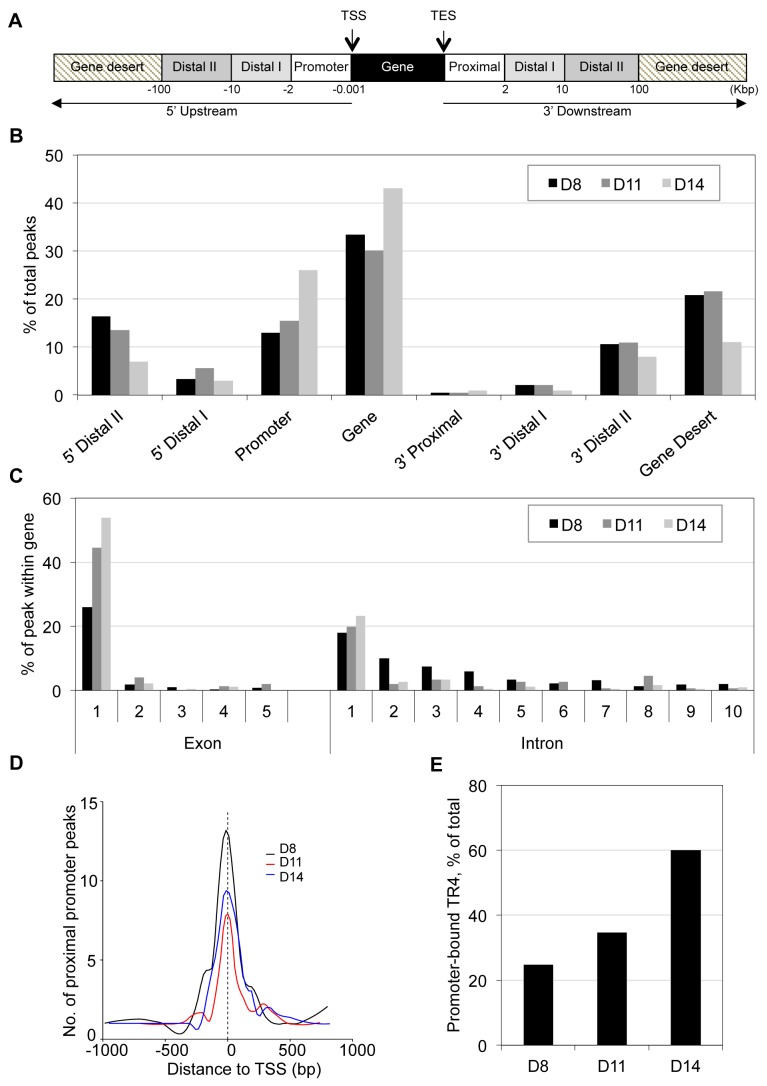
Genome-wide distribution of TR4 binding sites in differentiating human erythroid cells. (**A**) TR4 peak assignment is based on the distance from the peak center to the nearest transcription start site (TSS) of RefSeq genes. Once a peak has been assigned to the nearest gene, its location is classified into: within the gene (from TSS to transcription end site (TES)), 5′ upstream or 3′ downstream. Peaks in the 5′ upstream regions are further grouped into: promoter (from −0.001 to −2 Kbp), 5′ distal I (from −2 to −10 Kbp 5′) and 5′ distal II (from −10 to −100 Kbp 5′), and peaks located 3′ of the TES are grouped as 3′ proximal (from TES to 2 Kbp after TES), 3′ distal I (from 2 to 10 Kbp after TES) and 3′ distal II (from 10 to 100 Kbp 3′ to TES). Peaks >100 Kbp from TSS or TES are reported here to fall within gene deserts. (**B**) Distribution of TR4 binding peaks across the genome in day 8 (D8), 11 (D11) and 14 (D14) erythroid cells. (**C**) TR4 binding peaks that fall within genes are mapped within those gene exons and introns. Here, only the first 5 exons and the first 10 introns are shown. (**D**) Histogram illustrating the distribution of peaks in a window ±1 Kbp from the TSS (proximal promoter) at D8, D11 and D14 of erythroid differentiation. Peaks were combined into 15 bp bins. (**E**) Percentage of peaks that mapped to proximal promoter in D8, D11 and D14 differentiated erythroid cells.

Annotation of TR4 peaks was based on the shortest distance from the center of a peak to the transcription start site (TSS) of the nearest RefSeq gene. Each TR4 peak was then categorized according to position: within the gene or in the 5′ or 3′ flanking genomic sequences (**Figure 1A**). TR4 peaks lying in the flanking 5′ upstream and 3′ downstream were further subcategorized by their distance from the TSS or the transcription end site (TES) into promoter, distal 1, distal II and proximal, distal I, distal II, respectively (**Figure 1A**). Finally the most gene-distal TR4 peaks (>100 Kbp from TSS or TES) were classified as falling into a gene desert (**Figure 1A**).

The genome-wide TR4 peak distribution pattern in D8, D11 and D14 cells was similar, with the majority of peaks (45 to 69%) lying within the gene and promoter (**Figure 1B**). Of the TR4 peaks lying within the gene, the vast majority were located within the first exon or first intron (**Figure 1C**). We further determined that most TR4 peaks within the genes and promoters were positioned within ±1 Kbp of the TSS (referred to hereafter as the proximal promoter; **Figure 1D**). Of *all* TR4 peaks, 25%, 35% and 60% were located in the proximal promoter in D8, D11 and D14 cells, respectively (**Figure 1E**).

Unlike the increased percentage of peaks found in the proximal promoter as erythroid cells differentiated, the percentage of TR4 peaks in distal II (10 to 100 Kbp from TSS or TES) and gene deserts (>100 Kbp away from TSS or TES) lying both 5′ and 3′ to genes decreased between days 8 and 14 (**Figure 1B**). When we determined the peak distribution in the distal II regions by even finer increments, we found that the peak number decreased as the distance to the nearest gene increased (**Figure S4**).

### Identification of TR4 downstream targets by shRNA-mediated knockdown

**Figure 2:**
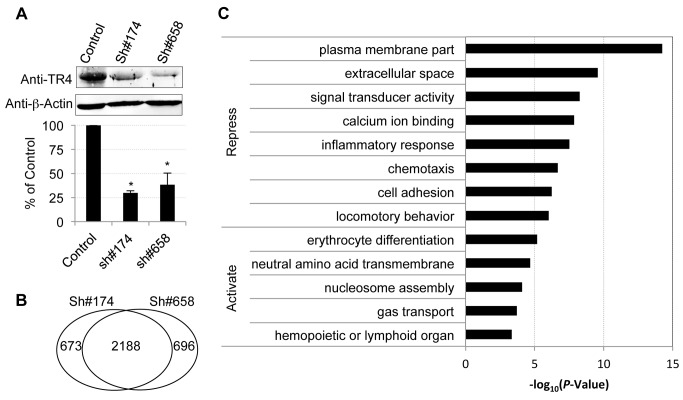
Characterization of TR4 downstream targets with lentiviral-mediated shRNAs. (**A**) The upper panel shows the immunoblots of TR4 and β-actin (internal control) in shRNA lentivirus (#174 and #658) or control virus infected cells at D11. The bar graph shows the relative abundance of TR4 normalized to β-actin and to control cells (*p<0.05 and error bars represent s.e.m.). (**B**) Venn diagram summarizing the common and unique differentially expressed genes after TR4 depletion with lentivirus sh#174 and sh#658. (**C**) The enriched GO terms for TR4 repressed and activated genes, respectively.

In order to identify TR4 regulatory target sequences, anti-TR4 lentiviral-mediated shRNA knockdown in differentiating erythroid cultures was performed. Cells were independently infected with empty control virus or two different TR4 shRNA-containing viruses (#174 or #658) 4 days after initiating erythroid differentiation. Forty-eight hours following infection, the cells were then subjected to puromycin selection. The knockdown RNA samples were harvested at day 11 for RNA-seq analysis using an Illumina HiSeq2000 platform. Prior to submitting the samples for RNA-seq, we confirmed that there was a significant reduction in TR4 protein levels (∼70%) in cells transduced with either shRNA lentivirus by immunoblotting (**Figure 2A**).

Overall, 15,024 (84% of expressed) genes met a minimum established threshold of 0.1 FPKM in at least one mRNA dataset (control, sh#174 or sh#658). After applying double cutoffs of fold change ≥2 and FPKM ≥0.1 in either shRNA-treated or control dataset, we identified 2,861, and 2,884 genes that were differentially expressed after infection with the anti-TR4-sh#174 or -sh#658 lentiviruses, respectively. Of these, 2,188 (∼76%) differentially expressed genes were common in both shRNA-treated datasets, which included both direct and indirect TR4 target genes (**Figure 2B** **; Table S1**). Of these common targets, 80% (1,775/2,188) were transcriptionally induced while the remaining 20% (433/2,188) were repressed after TR4 diminution, suggesting that TR4 acts predominantly as a (direct or indirect) repressor of target genes in differentiating erythroid cells.

Gene ontology (GO) analysis showed that TR4-repressed genes encode proteins that are components of the plasma membrane and extracellular matrix or are involved in diverse cellular processes such as signal transducing activity, calcium ion binding functions, inflammatory responses, chemotaxis and others (**Figure 2C**). In contrast, the TR4-activated genes encode proteins that are closely aligned with neutral amino acid transmembrane transporter activity and nucleosome assembly. Not surprisingly, the genes that are activated by TR4 are significantly enriched in erythroid functions such as erythrocyte differentiation and gas transport, including MAEA, ALAS2, EPB42 and others (**Figure 2C**).

### Genes bound by TR4 in their proximal promoter are direct TR4 regulatory targets

**Figure 3:**
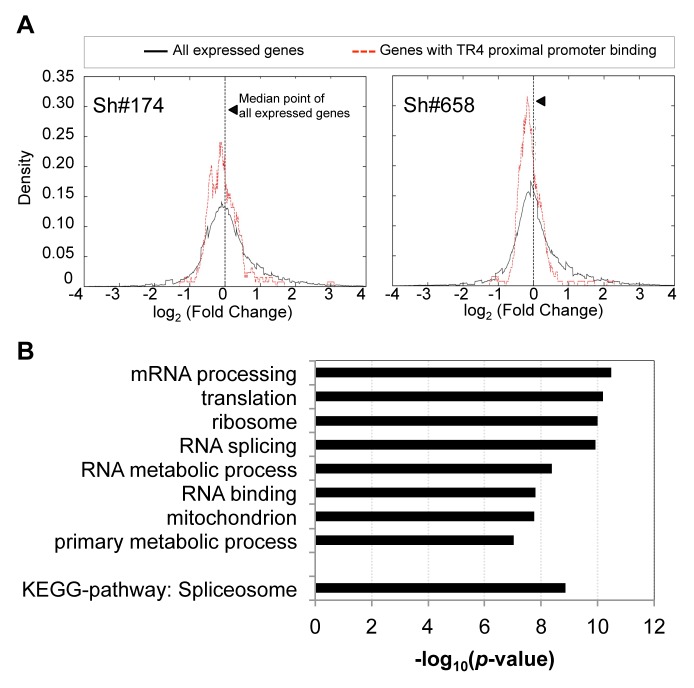
The expression of genes with TR4 bound at proximal promoter is reduced after TR4 depletion. (**A**) The distribution of the expression fold change between genes with TR4 bound in the proximal promoter region (dashed red lines) *vs*. that of all expressed genes (solid black line) after TR4 depletion by either lentivirus sh#174 (right panel) or lentivirus sh#658 (left panel), where positive values indicate an increase in expression after TR4 depletion. (**B**) Genes with TR4 bound at the proximal promoter are enriched in basic biological functions.

We noted earlier that a significant fraction (25% to 60%) of all TR4 peaks were found in gene proximal promoters (**Figure 1E**), where the enrichment of TR4 binding was significantly greater than in all other segments of the genome (**Figure S5A**). To determine whether these putative targets with TR4 proximal promoter binding are in fact regulated by TR4, we investigated the expression profile of these genes in D11 cells after lentiviral-mediated TR4 shRNA knockdown. For each of the 130 genes with TR4 peaks in the proximal promoter at D11 (**Figure 1E**), we plotted the distribution of their expression fold-change against that of *all* expressed genes using DESeq [28]. DESeq is an algorithm that identifies differentially expressed genes based on the negative binomial distribution of raw gene read counts rather than normalized FPKM values, thereby avoiding skewing that would be unduly influenced by abundantly expressed genes. When compared to the distribution of all expressed genes, the nearly entire cohort of 130 genes uniformly displayed lower expression levels after TR4 knockdown (*p* = 0.004 for virus sh#174, and *p* = 1.59×10^−6^ for virus sh#658, two-tailed t-test; **Figure 3A**), resulting in a shift of the distribution curve to the left. This suggests that TR4 is more likely to positively regulate these gene targets when bound at their proximal promoter. GO term analysis revealed that these TR4 direct targets were significantly enriched in fundamentally biological processes (mRNA processing, translation, ribosomal subunits, RNA splicing and RNA metabolic processes; **Figure 3B**). Perhaps consistent with these basic physiological functions, these TR4 direct targets were expressed abundantly (**Figure S5B; Figure S3B**). Since genes encoding such critical physiological functions are more likely to be protected by redundant or compensatory activity, this could partially explain why we did not detect greater than 2-fold change in expression of most of these 130 genes (<2-fold; **Figure 3A**) when the TR4 levels were reduced by only 70% in shRNA-treated D11 cells.

Of the 375 peaks identified in D11 cells (**Table 1**), 173 (46%) were located >10 Kbp away from the nearest gene. These gene-distal TR4 peaks are potentially embedded within distant enhancer or silencer gene regulatory elements. Since enhancers and silencers have been identified to sometimes lie great distances from the gene they regulate [29], it is not readily apparent what are the putative target genes for these TR4 distal sites [30]. Here, we applied a strategy to assign these distal peaks to the nearest differentially expressed gene, which could be identified after both lentiviruses were used for TR4 depletion (using the double cutoffs of fold change ≥2 and FPKM ≥0.1; 2,188 common targets; **Figure 2B**), rather than to the physically nearest RefSeq gene. These 173 distal peaks could be uniquely assigned to 65 closest differentially expressed genes, with a median distance of 901 Kbp. The expression of 78% (51/65) of these genes associated with distal TR4 peaks was induced after TR4 depletion in shRNA-treated cells, suggesting that when bound far away for the regulated gene, TR4 seems to most often act as a repressor.

### Motif analysis reveals that DR1 is overrepresented in TR4 target promoters

Nuclear receptors recognize the hexameric consensus half-site AGAACA or AG(G/T)TCA [8], [31] that can be spatially arranged as direct repeats (DRs), everted repeats (ERs) or inverted repeats (IRs) separated by a small variable number (0 to 8) of spacer nucleotides [32]. Both *in vitro* and *in vivo* studies have shown that TR4 can bind to DR elements with 0 to 5 spacer nucleotides [9], [19], [25], [33], [34]. However, the *in vivo* TR4 consensus recognition motif in any primary tissue is unknown.

**Figure 4:**
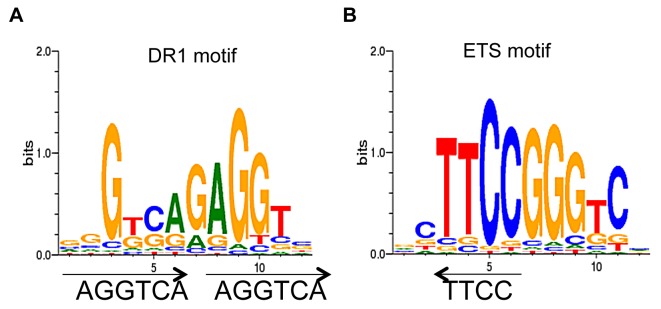
*De novo* motif analysis of TR4 peaks located at the proximal promoter. Motif analysis of peak sequences (250 bp from peak center) identifies DR1 (**A**) and ETS (**B**) motifs as overrepresented among the peaks located at the proximal promoter in D14 erythroid cells.

We used CoreSearch, the *de novo* transcription binding site discovery program, from the Genomatix data analysis package as well as DREME [35] to identify overrepresented transcription factor binding site motifs within the TR4 peaks. Motif analysis of peaks in the proximal promoters was performed by examining the 250 nucleotides from the center of each peak at D8, 11 and 14. DR1 elements with an A or G as the spacer nucleotide were enriched at all differentiation stages. As a representative example, the enriched DR1 motif (11%) from D14 cells is shown in **Figure 4A**. This observation is consistent with a previous study in which we determined that a DR1 motif was also overrepresented in TR4 ChIP-seq peaks lying within ±1 Kbp of the nearest TSS in four ENCODE cell lines [HepG2 (21%), HeLa (21%), K562 (35%) and GM12878 (43%)] [25]. As in this previous study [25], here we also found that the ETS family motif was overrepresented (15%) in TR4-occupied peaks (**Figure 4B**).

**Figure 5:**
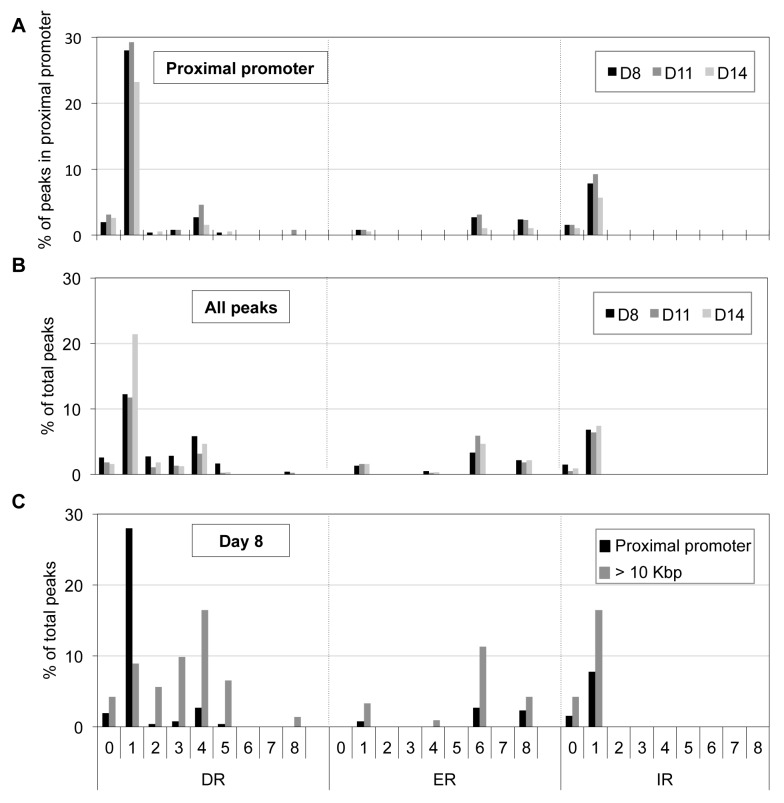
Distribution of potential NR binding sites in TR4-bound peaks. Peak sequences (250 bp from peak center) were interrogated using NHR-scan for the presence of direct repeat (DR), everted repeat (ER) and inverted repeat (IR) motifs with 0–8 spacer nucleotides in D8, D11 and D14 (**A**, **B**) or in D8 (**C**) cells. The percentages of each motif type in peaks located at the proximal promoter (**A**), in all peaks (**B**), and in peaks at proximal promoter *vs*. >10 Kbp from genes (**C**) are represented graphically.

We employed a second nuclear hormone receptor binding site prediction program, NHR-scan, to identify enriched repeat elements [32]. Of the DR elements, we identified DR1 sequences comprising the major site (29% of the 130 peaks at D11) with DR4 (5%) and DR0 (3%) motifs as minor constituents (**Figure 5A**). Intriguingly, other repeat elements such as IR1 (9%), ER6 (3%) and ER8 (3%) were also enriched, albeit modestly, among the TR4 proximal promoter peaks (**Figure 5A**). We additionally subjected the 250 nucleotides from the center of all peaks (**Table 1**) to motif analysis using NHR-scan. DR1 was still the most prevalent in all peaks, but it was not as highly represented as in the proximal promoter peaks (12% *vs*. 29% in D8 cells, respectively) (**Figure 5B**).

We next asked if there is a bias of certain DR or ER or IR motifs in the proximal promoter *vs*. distant sites (>10 Kbp away from the nearest gene). Of the D8 peaks (total 1025), 257 and 490 peaks were located in the proximal promoter or >10 Kbp away from the nearest gene, respectively. Comparing these two groups, we found that only the frequency of the DR1 motif was reduced (from 28% to 8%), but all other motifs significantly increased [(DR4 (from 3% to 16%), ER6 (from 3% to 11%) and IR1 (from 8% to 16%); **Figure 5C**]. Similar results were observed for D11 and D14 sites (data not shown). Thus, there appears to be a repeat element motif bias in gene-proximal *vs*. gene-distal regulatory sequences. While DR1 is markedly over-represented in proximal promoter binding sites, other nuclear receptor binding motifs (such as DR4, ER6, and IR1) occur more often in distant regulatory regions, possibly hinting that TR4 might partner with nuclear receptors other than TR2 to exert long-range regulatory effects.

We next tested the alternative partner hypothesis using MEL cells that stably expressed a biotin recognition sequence-tagged TR4 cDNA and bacterial *birA* biotin ligase [11], [36]. Biotinylated complexes were purified from nuclear extracts of MEL cells that stably expressed either birA alone or birA plus biotinylated-TR4 using streptavidin beads. The proteins eluted from the beads were resolved by SDS-PAGE and immunoblotted using antibodies against TR2, RAR, RXR, and LXR, which have been reported previously to bind to DR4, ER6 and IR1 motifs [37].

**Figure 6:**
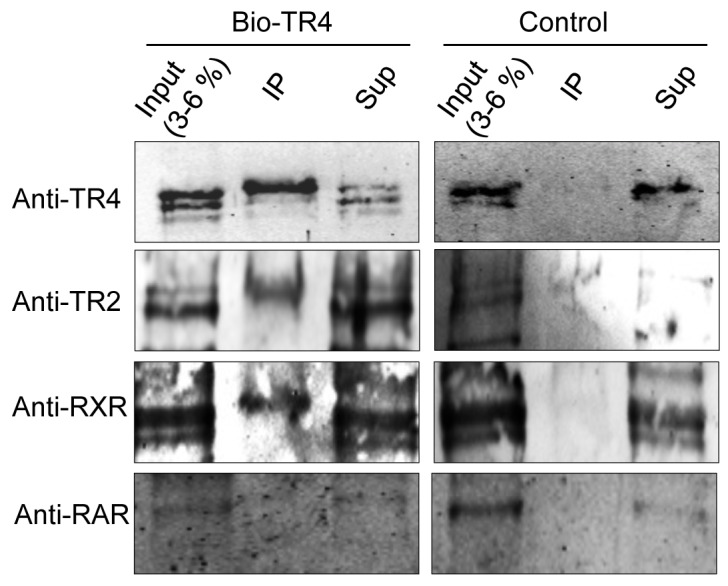
Identification of proteins precipitated with streptavidin beads from MEL cells expressing biotin-tagged TR4. Nuclear extracts were prepared from MEL cells expressing the biotin ligase gene (birA) without (control) or with biotin-tagged TR4 and then incubated with streptavidin beads. Proteins precipitated with the beads (Bound), 3∼6% of the input, and equal amount of supernatants (Sup) were subjected to SDS-PAGE, followed by immunoblotting with antibodies that recognize TR2, RXR and RAR.

As anticipated, we readily detected TR2 among the products that were in complex with biotinylated TR4, but not in control nuclear extracts (**Figure 6**). Interestingly, we also detected a weak interaction between TR4 and RXR (**Figure 6**), verifying a report in a previous study [38]. However, we failed to detect any interaction between TR4 and RAR (**Figure 6**), and failed to detect any LXR expression in MEL cells (data not shown). Thus, the data suggest that TR4 can heterodimerize *in vivo* with RXR in addition to TR2. We do not know the relative abundance of the RXR:TR4 complex in relation to that of the TR2:TR4 complex.

### Gene distant TR4 sites are in domains with enhancer regulatory potential

**Figure 7:**
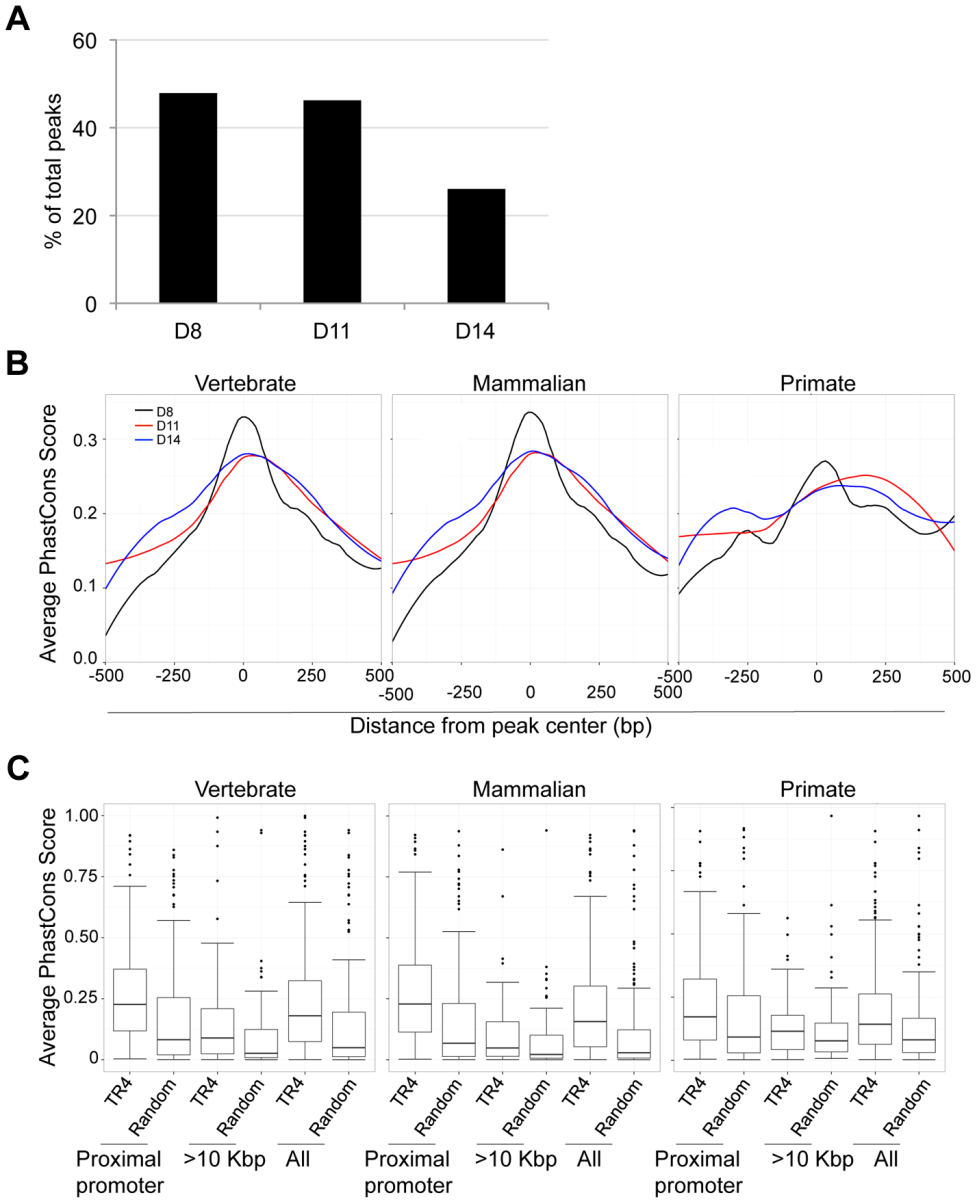
Sequence conservation among TR4 peaks. (**A**) The percentage of peaks located >10 Kbp from the nearest RefSeq genes decreased from D8 to D14 of differentiation. (**B**) For peaks located at the proximal promoter, the average PhastCons score of each nucleotide within a peak (500 bp from peak center) across vertebrate, mammalian or primate species are graphed. The center of each peak is defined as “0”. (**C**) Comparison of the average PhastCons scores of peak sequences and random control sequences in the TR4 peaks located at proximal promoter, >10 Kbp from genes or in all identified peaks at D14 differentiation.

We observed that peaks located far from gene TSSs were enriched for a variety of NR repeat element recognition sites other than DR1 (**Figure 5C**). At 8, 11 and 14 days of erythroid differentiation, 48% (490), 46% (173) and 26% (83) of total peaks were located more than 10 Kbp from the closest gene, respectively (**Figure 7A**). We reasoned that if these distal TR4-bound sites are located in genomic sequences that display regulatory activity (such as enhancers), then they might show interspecies sequence conservation [29].

To test this hypothesis, we retrieved PhastCons scores for all TR4 peaks from the UCSC genome browser, which represents a base-by-base conservation score at every nucleotide position between the genomic sequences of multiple species [20], [31]. We calculated and graphed the average PhastCons score of all peaks from the peak center toward both ends [*i.e*. the sum of PhastCons score of nucleotides at specific positions (peak center defined as “0” and followed −2, −1, +1, +2 *etc*. toward both ends) divided by the total peak number] at each differentiation stage. The analysis revealed that the sequences at the center of all peaks across differentiation days 8,11 and 14 were generally more highly conserved than the flanking sequences across either vertebrate, mammalian or primate species (**Figure 7B**, data not shown). However, the peaks in the proximal promoters (**Figure 7B**), when compared to peaks lying far away from the nearest genes, exhibited the greatest conservation.

Next, we extracted the 250 bp from center of each peak and recomputed the average PhastCons score for each peak by dividing the sum of the PhastCons scores of individual nucleotides by peak length. For each sequence in three peak categories (proximal promoter, >10 Kbp from the nearest genes and all peaks), a random sequence of the same length (250 bp) and GC content from the same chromosome was used to compute a background conservation score. These analyses revealed that the peaks in the proximal promoters of D8, D11 and D14 cells showed the highest conservation across vertebrate, mammalian and primate species (**Figure S6;** **Figure 7C**). Furthermore in D14 cells, the TR4-bound sequences located >10 Kbp from the nearest gene were more highly conserved among all vertebrate, mammalian and primate species (**Figure 7C**). This suggests that distal TR4-bound sequences have potential (enhancer or silencer) regulatory activity during late erythroid differentiation.

We therefore next asked if any enhancer marks were present in the sequences (±2 Kbp) immediately adjacent to each TR4 peak that was located >10 Kbp from the nearest gene using ENCODE data from analysis of the K562 erythroid cell line [39]. Chromatin enhancer-related signatures include active (transcriptional coactivator P300 binding [40]–[42] and H3K27ac [43]), inactive (poised; H3K27me3 [44], [45]) and persisting (H3K4me1 [46], [47]) histone epigenetic modifications. We found that more than half of the TR4 distant peaks co-localized with relevant enhancer marks in K562 cells (**Table 2**), supporting the hypothesis that the TR4 peaks located >10 Kbp from genes could have gene regulatory (in this case, enhancer) activity.

**Table 2:** Number and percentage of epigenetic enhancer marks that co-localize with TR4 distal peaks.*

	D8	D11	D14
**P300**	29 (*6*)	23 (*13*)	16 (*19*)
**H3K27ac**	35(*7*)	16 (*9*)	11(*13*)
**H3K4me1**	59 (*12*)	28 (*16*)	15(*18*)
**H3K27me3**	166 (*34*)	31(*18*)	18 (*22*)

*Within ±2 Kbp of TR4 distal peaks.

We next performed *de novo* motif analysis to identify other enriched transcription factor binding sites (TFBS) within distant TR4 peaks using CoreSearch and DREME. This analysis did not reveal any single overrepresented TFBS at any stage of erythroid differentiation (data not shown). For example, at day 14, a variety of sites (including MZF1 or GATA1) were scattered among these peaks, suggesting that TR4 might interact directly or indirectly with these transcription factors to regulate specific target genes from great distances.

We further queried the sequences of each peak (250 bp) in the proximal promoters and lying >10 Kbp from genes using the “overrepresented transcription factor binding modules” analytical tool from Genomatix. This analysis tool searches for all TFBS and generates statistics on possible TFBS pairs (modules) within the input sequences over the genomic background. Of the top ten transcription factor families that could interact with the NR2 family (to which TR4 belongs), there was no overlap in the TFBS pairs between gene-proximal and -distal TR4-bound peaks, possibly suggesting that TR4 collaborates with distinct transcription factors in gene-proximal and -distal regulatory elements in a highly tissue-specific manner to exert its effects on transcription (**Table 3**).

**Table 3:** Overrepresented transcription factor binding motifs near NR subfamily 2 promoter binding sites *vs*. those near NR2 subfamily binding sites lying >10 Kbp away from the closest gene.

	Modules with NR2F*	Prom. assoc. known^1^	Expected (genome)^2^	Over representation (genome)^3^	Z-Score (genome)^4^
**<1 Kbp**	NRF1	yes	0.58	114.14	85.38
	ZF5F	yes	1.34	47.79	53.72
	E2FF	yes	11.73	13.89	44.02
	EGRF	yes	8.8	12.39	33.62
	XCPE	yes	1.61	27.39	33.05
	SP1F	yes	9.25	10.59	29.01
	HDBP	yes	0.53	41.5	28.8
	ZF02	yes	12.75	9.02	28.51
	MTEN	yes	1.44	22.96	25.91
	CTCF	yes	5.47	11.52	24.39
**>10 Kbp**	PTF1	no	2.09	6.22	7.2
	EREF	no	6.09	3.94	7.05
	NEUR	no	6.7	3.13	5.33
	HAND	no	16.39	2.26	4.97
	PERO	no	9.16	2.62	4.74
	NRSF	no	3.39	3.54	4.41
	HICF	no	2.8	3.57	4.01
	HESF	no	4.27	3.04	3.98
	IRXF	no	6.43	2.49	3.58
	NF1F	no	5.63	2.31	2.9

*Transcription factor binding sites that are statistically overrepresented and lying within 1 Kbp of any NR2 subfamily member (ARP1, COUP, HNF4, HPF, PNR, TR2 or TR4) binding site; comparison is between the top 10 TF family motifs near NR2 promoter sites versus the top 10 TF family motifs near NR2 sites lying greater than 10 Kbp from the nearest gene.

1 TF families known to associate with promoters; yes or no.

2 Expected number of matches in an equally sized sample of the genomic background.

3 Overrepresentation against the genomic background: Fold factor of match numbers in input sequences compared to an equally sized sample in the background (i.e. found *vs*. expected).

4 Z-score [70] of overrepresentation against the genomic background: A Z-score of <−2 or >2 is statistically significant and corresponds to a *p*-value of about 0.05.

In summary, we found that TR4 peaks located far from genes had significant cross species conservation and some of these also bore enhancer signature epigenetic marks. However, using multiple analysis tools, we were unable to identify any preferred transcriptional co-effectors in the hypothesized TR4-mediated long-range gene regulatory sites.

## Discussion

In this study, we surveyed all orphan nuclear receptor TR4 binding sites throughout the genome as well as its downstream targets using ChIP-seq and RNA-seq in human primary CD34^+^ cells as they differentiate into erythroid cells. These analyses revealed 1,025, 373 and 325 TR4 binding sites in erythroid cells at early through late differentiation stages (8, 11 and 14 days after the onset of differentiation). Of these binding sites, the frequency of TR4 proximal promoter binding rose from 25% to 60% between the proerythroblastic and reticulocyte stages (from day 8 to day 14). *De novo* motif analysis revealed that in proximal promoters, DR1 NR binding sites and ETS motifs were the most overrepresented sequences. This conclusion is consistent with our previous TR4 ChIP-seq study examining TR4 binding in four ENCODE cell lines K562, HepG2, HeLa and GM12878 [25]. While TR4 has been reported to bind DR elements with 0 to 5 spacer nucleotides [18], [19], our past and present *in vivo* genome-wide TR4 ChIP analyses indicate that TR4 preferentially binds to DR1 elements in the proximal promoters in ENCODE cell lines [25] and in primary human erythroid cells (this study).

Our combined ChIP-seq and RNA-seq data suggest that TR4 acts primarily as a transcriptional activator at the proximal promoter of target genes, and most of these are involved in fundamental physiological functions (such as mRNA processing, ribosome synthesis, RNA splicing, and primary metabolic processes) during erythropoiesis. This observation also agrees with an earlier study in which we reported that target genes (shared by all four ENCODE cell lines) exhibiting TR4 proximal promoter binding encode constituents of the spliceosome and the ribosome or are involved in other mRNA metabolic processes [25]. Taken together, we conclude that TR4-regulated target genes control fundamental biologic processes that are common to many different cell types that express this (essentially ubiquitous) orphan nuclear receptor.

The expression of most TR4 direct targets with peaks in the proximal promoter was only weakly reduced in abundance (<2-fold change) after TR4 knockdown. This could be a consequence of the residual TR4 protein (30%) in the knockdown cells that may still be able to execute much of TR4 normal function, or of compensation by other nuclear receptor such as TR2, or both. In an earlier study, we observed that the combined loss of *Tr2* and *Tr4* alleles leads to greater γ-globin induction in definitive erythrocytes of mutants bearing a human β-globin YAC transgene than mice bearing single *Tr2* or *Tr4* null mutations [12], implying that at least to some extent, TR2 and TR4 fulfill overlapping and partially compensatory functions.

More recently, with the advent of deep sequencing technologies the genome-wide nuclear receptor binding site analyses of ERα, AR, GR, PPARγRXR and VDR have suggested that nuclear receptors act far more frequently (>60%) from distant sites than do other classic transcription factors that bind to the proximal promoters [48]–[54]. Therefore, it is perhaps not surprising that 48%, 46% and 26% of all TR4 ChIP-seq peaks after 8, 11 and 14 days of erythroid differentiation induction, respectively, were located more than 10 Kbp from the nearest gene.

One major conceptual and experimental challenge is how we might link the binding of distant nuclear receptor sites to their *bona fide* target genes. For example, in the present study, when we assigned the TR4 distal peaks to the closest differentially expressed gene, instead of the physically closest gene, the median distance was more than 900 Kbp. In other nuclear receptor studies, the median distance from GR binding sites to their closest activated or repressed targets was 11 Kbp or 146 Kbp, respectively [55], while the majority of ERα binding sites were located within 50 Kbp of ER-regulated genes [52].

Both sequence conservation and the coincidence of enhancer epigenetic signatures indicate that a significant fraction of TR4 distal binding sites have *cis*-regulatory potential. However, the mechanism underlying such distal regulation is still largely unknown, although it has previously been speculated that chromatin modifying cofactors might aid nuclear receptors in regulating transcriptional targets from a distance [56], [57]. With the advent of high throughput chromosome conformation capture (3C) such as Hi-C [58], [59] or tethered conformation capture (TCC) [60], which can decipher genome-wide chromatin contacts on the megabase scale, we may soon be able to readily capture insights into long distance gene regulation independent of gene proximity.

When we restricted the binding site motif analysis exclusively to repeat elements using the NHR-scan program, we discovered that while DR1 was the most represented motif in TR4 proximal promoter bound peaks, other nuclear receptor binding motifs (DR4, ER6, and IR1) were more frequently represented in peaks lying greater than 10 Kbp away from the nearest gene. Hence, we speculate that within the nuclear receptor superfamily, TR4 binds to promoter DR1 element of its targets most often by heterodimerizing with TR2, but that TR4 exerts its long-range regulatory effects by binding to DR4, ER6 and IR1 gene-distal repeat elements, perhaps in association with other nuclear receptors such as RXR.

One curiosity we report was that over half of the TR4 binding peaks reported here did not contain any recognizable nuclear receptor repeat element. It has been proposed that nuclear receptors can modulate gene expression by interfering with other transcription factors to alter target gene expression, referred to as “transcriptional crosstalk” [31], [61]. For example, among ERα genome-wide enriched peaks, the binding motifs of transcription factors such as FOXA1, Sp1, KNX3.1 and others were also enriched [48], [62], [63]. Similarly, in the case of PPARγChIP-chip or ChIP-seq peaks, the binding motif for C/EBP family transcription factors was overrepresented [53], [64]. Our previously reported ChIP-seq data examining several ENCODE cell lines [25] and the present ChIP-seq data investigating TR4 binding in primary differentiating cells indicate that TR4 also shares such transcriptional machinery by “transcriptional crosstalk” with ETS family members in promoters and with transcription factors such as MZF1 and GATA1 at putative distal enhancer sites.

In this study, we also found that TR4 protein was expressed more abundantly at later erythroid differentiation stages. Based on the expression profiling of differentially expressed genes (fold change ≥2 and FPKM ≥0.1 in any dataset) after TR4 depletion, we found that TR4 most often acts as a repressor. During the progression of erythroid differentiation the majority of genes become repressed and chromatin becomes condensed. The more abundant expression of TR4 during later stages might be required to fulfill these suspected repressor functions during differentiation. However, it seems contradictory that TR4 increases in expression as the total number of TR4 binding peaks diminish as erythroid cells mature. One possible explanation is that TR4 might be temporarily required for the initiation of transcriptional repression by recruiting repressive cofactors to target gene loci thereby facilitating chromatin condensation. During the process of chromatin condensation, after TR4 has fulfilled its function (bringing corepressors to chromatin) it might be released from its target binding sites. That could partially explain why we observe an opposite trend of increasing TR4 expression at the same time as the number of TR4 binding sites diminishes later during erythroid differentiation.

Finally, we note that reducing the abundance of TR4 in human erythroid cells resulted in the down-regulation of genes enriched in erythroid differentiation and gas transport. However, the closest TR4 binding to these genes was >1 Mbp away. In this regard, it is difficult to distinguish whether these genes are under TR4 long-range regulation or whether they are only indirectly affected by TR4 depletion. While we have definitively documented that TR2/TR4 heterodimer binding at the γ-globin promoter DR1 site was a prerequisite for γ-globin silencing in definitive erythroid cells [9], we did not detect a TR4 peak at this DR1 element in this study. We surmise that the lesser enrichment of TR4 at the γ-globin DR1 element might be beyond the sensitivity of current sequencing depth and/or might be affected by limited biological replicates (2 biological replicates in current study) [65], [66]. In the future, the combination of ChIP-seq performed with increasing sequencing depth, expression profiling and Hi-C or TCC may shed more light on these enigmatic nuclear receptor regulatory mechanisms, and with those insights, we may be able to more confidently identify a greater number of *bona fide*, direct target genes.

## Materials and Methods

### Ethics statement

Research on human specimens was conducted in full compliance with federal and institutional regulations and guidelines.

### *Ex vivo* erythroid differentiation of human CD34^+^ progenitor cells

Cryopreserved primary human CD34^+^ hematopoietic progenitor cells, isolated from the peripheral blood of healthy adult donors following granulocyte colony-stimulating factor (G-CSF) mobilization, were purchased from the Fred Hutchinson Cancer Research Center. Briefly, these cells were cultured *ex vivo* using a two-phase culture method, as described previously [10], [67]. On differentiation days 8, 11 and 14, cells were separately harvested for chromatin and RNA isolation for analyses in ChIP-seq and RNA-seq experiments, respectively.

### ChIP assay and library construction

ChIP was performed as described previously [20], [25], with the exception that the sonication was performed to generate DNA fragments of 500–700 bp. In brief, 10^8^ cells were harvested on culture days 8, 11 or 14 and cross-linked with formaldehyde. Chromatin in complex with TR4 was then sonicated and incubated with rabbit anti-TR4 antibody [10]–[12], [25] at 4°C overnight in IP dilution buffer. The precipitated protein bound DNA fragments were then reverse cross-linked and purified for use in library construction. Briefly, ChIP DNA was quantified using a QuBit and the size distribution of the recovered fragments was analyzed using an Agilent Bioanalyzer. Library construction was performed using the IntegenX ChIP-seq sample preparation kit as per manufacturer's instructions. The libraries were amplified by PCR (15 cycles) and then purified using Agencourt XP beads with a DNA fragment size selection of 500–700 bp. ChIP DNA libraries were analyzed on a Bioanalyzer and qPCR assay to assess the library quality and pooled in equimolar ratios before sequencing. Cells for the ChIP experiment were harvested in duplicate from independent cell culture.

### Sequencing and data analysis

ChIP-enriched and input control DNA libraries were sequenced on an Illumina HiSeq 2000. DNA sequencing reads that passed FastQC quality control were aligned to the reference genome (hg19, Feb 2009), and peaks were identified using MACS (version 1.4) [68]. ChIP-seq data have been deposited in the NCBI Gene Expression Omnibus (accession number GSE54759).

### Depletion of TR4 mRNA using lentiviral shRNAs

The pLKO.1-puromycin resistant lentiviral shRNAs (short hairpin RNAs) used to reduce TR4 mRNA levels were purchased from Sigma-Aldrich (TRCN0000245174 and TRCN0000021658). The pLKO.1 control vector was generated as described previously [10]. Lentivirus packaging into virions was performed by University of Michigan vector core facility. For infection, cells were exposed to virus on day 4 for 24 hours and then selected using 1 µg/µl puromycin addition to the media on days 6 through 14. On day 11, the cell were harvested for examination of TR4 depletion and then subjected to library construction and sequencing. Gene Ontology analysis was performed using the **D**atabase for **A**nnotation, **V**isualization and **I**ntegrated **D**iscovery (DAVID) webtool (*p*-value <0.05) [69].

### RNA-seq library construction and sequencing

Total RNA from CD34^+^ cells on days 8, 11 or 14 was purified by ISOGEN (Nippon Gene). RNA integrity and quality were verified using a 2100 Bioanalyzer (Agilent Technologies). The RNA integrity number (RIN) for all RNA samples used in this study was 10, indicating that the RNA quality was maximal.

Construction of the RNA-seq libraries was performed according to the manufacturer's instructions (Illumina). In brief, polyA^+^ RNA was purified using poly-dT oligo-attached magnetic beads from 10 µg of total RNA extracted from day 8, 11 and 14 differentiated erythroid cells, and then sheared into short pieces. These RNA fragments were reverse-transcribed using random primers into double-stranded cDNA fragments. Finally, these cDNA fragments were end repaired and Illumina adapters were appended to both ends. After PCR amplification, cDNA fragments of approximately 200 bp in length were selected for library generation and were sequenced in a paired-end 72-bp sequencing format using the Illumina Genome Analyzer IIx system at the University of Michigan DNA Sequencing Core. For erythroid cells in which TR4 was depleted using lentiviral shRNAs, the process was the same except that the sequencing platform was a paired-end 50-bp format on an Illumina Hiseq2000.

### Motif analysis

Both DREME [35] and CoreSearch (the motif identification program in the Genomatix software) were used to identify statistically overrepresented motifs retrieved from the UCSC Genome Database (hg19, Feb 2009) that lay within 250 bp of the TR4 binding peaks. Recovered motifs identified by DREME were further annotated using TOMTOM [70] within the JASPAR CORE vertebrate transcript factor binding motif database webtool [71]. For the core search, in addition to the default setting, the following settings were applied: length of core  = 9; minimal matrix similarity  = 0.6; maximum number of motifs  =  unlimited. We additionally performed the motif analysis using a nuclear receptor binding site prediction program (NHR-scan) with default parameters [32].

### Identification and quantification of gene expression by RNA-seq

For RNA-seq data analysis, we aligned the raw reads from ELAND to the human genome (build hg19, 2009) using TopHat (version v2.0.10) with default settings [71], [72]. We used Cufflinks (version v2.1.1) [73] to identify transcripts and determine their expression levels [Fragments Per Kilobase of transcript per Million mapped reads (FPKM)]. The expression data has been deposited in the NCBI Gene Expression Omnibus (GSE54602 and GSE54760).

### Streptavidin pulldown of biotin-tagged TR4 in MEL cells

The generation of biotin-tagged TR4 in MEL cells was described previously [11], [36]. Nuclear extracts were prepared [36], [74], and then incubated overnight with streptavidin beads at 4°C on a rotating wheel. The next day, the beads were washed 3 times with ice-cold PBS plus 0.5% Tween20 and twice with ice-cold 1X Laemmli sample buffer, and then boiled in SDS-PAGE sample loading buffer for 5 minutes.

### Immunoblotting

After SDS-PAGE, the proteins were transferred to a nitrocellulose membrane (Li-Cor) and probed with TR4, RXR (Santa Cruz, SC-774), RAR (Santa Cruz, SC-773) as well as fluorescence-conjugated secondary antibodies (Li-Cor). The proteins were visualized on an Odyssey infrared imaging system (Li-Cor).

### Conservation analysis

The center 250 bp of each ChIP-seq peak was examined for interspecies sequence conservation by calculation of PhastCons scores. In detail, the PhastCons scores of 46 vertebrate species and two subsets including 33 placental mammal species and 10 primate species were first extracted from the UCSC table browser (http://hgdownload.cse.ucsc.edu/goldenPath/hg19/phastCons46way/README.txt). The total PhastCons score of the individual nucleotides was then divided by the length (250 bp) to achieve an average PhastCons score for each peak.

### Validation of ChIP assays

Ten genes at each differentiation stage that exhibited TR4 proximal promoter binding were selected for validation. For ChIP-seq data analysis, the peak fold-enrichment was defined as the fold change of TR4-enriched reads over that of input at any specific genomic locus, while for ChIP-qPCR the fold-enrichment was determined by TR4 antibody in comparison to an IgG control. To properly compare the correlation of these two methods, we normalized the fold change to that of day 8 in both assays to calculate the Pearson correlation coefficient. For RNA-seq and RT-qPCR correlation analysis, 18s rRNA was used as the internal control and values were again normalized to day 8. We input the 250 bp of sequence flanking the center of any TR4 binding peak to design ChIP-qPCR primers using Primer Express (v 3.0.1) and, if there was a predicted DR, IR or ER repeat element (i.e. a potential NR binding site), we ensured that the amplicon included that region. For RNA-seq data validation, we randomly selected twenty genes that were broadly expressed and performed RT-qPCR. Primer pairs were designed to span exon/intron junctions. The methods for RNA extraction, cDNA synthesis and qPCR were as described previously [10], [11].

## Supporting Information

Figure S1 Expression of TR4 as human erythroid cells differentiate *ex vivo*. (**A**) Immunoblots of TR4 and β-actin (internal control) during erythroid differentiation on day 8, 11 and 14. (**B**) Quantification of the TR4 expression by normalized it to the signal intensity of β-actin (**p<0.01 and error bars represent s.e.m.).
(PDF)

Figure S2 A representative view of TR4 binding as erythroid differentiation progresses at the proximal promoter of the CHMP4B gene by integrative genomics viewer (IGV). The red lines indicated TR4 binding at differentiation days 8, 11 and 14; the blue lines represent the corresponding input controls in the same sequences.
(PDF)

Figure S3 Validation of TR4 ChIP-seq and RNA-seq data. (**A**) TR4 binding detected by ChIP-seq was validated by ChIP-qPCR. (**B**) Gene expression detected by RNA-seq was validated using RT-qPCR. The relative fold change in TR4 enrichment or the gene expression in all assays was normalized to that of day 8 erythroid cells and the data are presented as binary logarithms (log_2_). The Pearson's correlation coefficient, r, for each data set is indicated.
(PDF)

Figure S4 Distribution of peaks located >10 Kbp from the nearest genes. Peaks located 10–100 Kbp upstream (5′ distal II), 10–100 Kbp downstream (3′ distal II) or >100 Kbp either 5′ or 3′ from the nearest genes (gene desert) are graphed. The bin size is 10 Kbp for 5′ distal II and 3′ distal II and 100 Kbp for the gene deserts.
(PDF)

Figure S5 TR4 peaks close to TSSs are more enriched and are associated with higher gene transcription levels. Box-and-whisker diagrams illustrating the correlation of peak enrichment (**A**) or gene expression (**B**) given the distance (from peak center) to the nearest TSS in day 8, 11 and 14 erythroid cells. The gene expression profile was generated by RNA-seq and measured as FPKM.
(PDF)

Figure S6 Comparison of the average PhastCons scores of TR4 peaks and random control sequences among the peaks located at the proximal promoter, >10 Kbp from genes or of all identified peaks at day 8 (upper panels) or day 11 (lower panels) of differentiation.
(PDF)

Table S1 Differentially expressed genes in differentiated CD34^+^ cells after TR4 depletion by two different shRNA-encoding lentiviruses (sh#174 and sh#658; Materials and Methods).
(PDF)
