# Supplementary figures and images for: Biased, Non-equivalent Gene-Proximal and -Distal Binding Motifs of Orphan Nuclear Receptor TR4 in Primary Human Erythroid Cells
Source: PLoS Genet. 2014 May 8;10(5):e1004339. doi: 10.1371/journal.pgen.1004339 (PMC4014424; doi:10.1371/journal.pgen.1004339)

Supplemental Figure 1

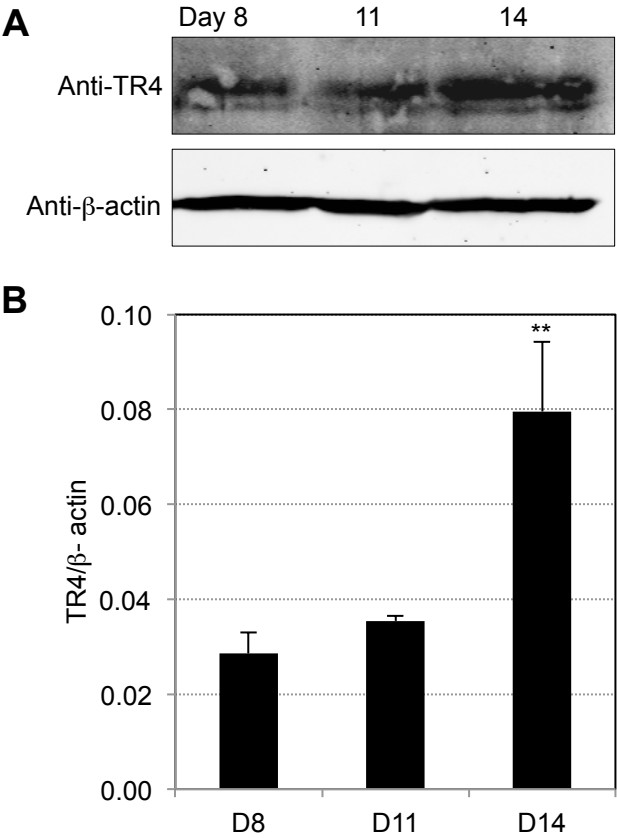

Supplement: Figure S1 — Expression of TR4 as human erythroid cells differentiate ex vivo. (A) Immunoblots of TR4 and β-actin (internal control) during erythroid differentiation on day 8, 11 and 14. (B) Quantification of the TR4 expression by normalized it to the signal intensity of β-actin (**p<0.01 and error bars represent s.e.m.). (PDF) [file pgen.1004339.s001.pdf]

Supplemental Figure 2

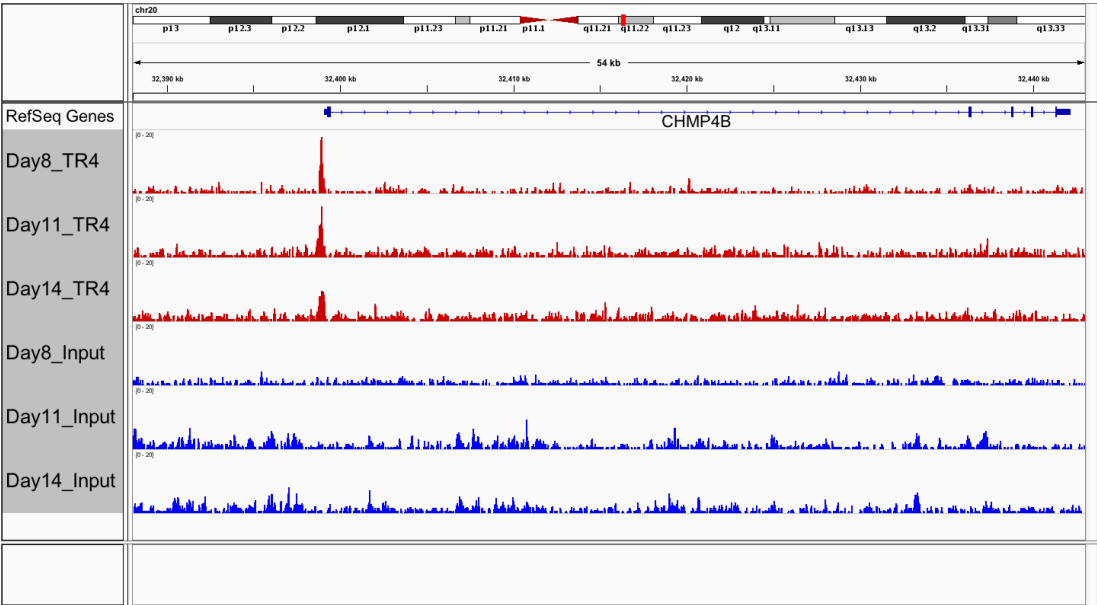

Supplement: Figure S2 — A representative view of TR4 binding as erythroid differentiation progresses at the proximal promoter of the CHMP4B gene by integrative genomics viewer (IGV). The red lines indicated TR4 binding at differentiation days 8, 11 and 14; the blue lines represent the corresponding input controls in the same sequences. (PDF) [file pgen.1004339.s002.pdf]

Supplemental Figure 3

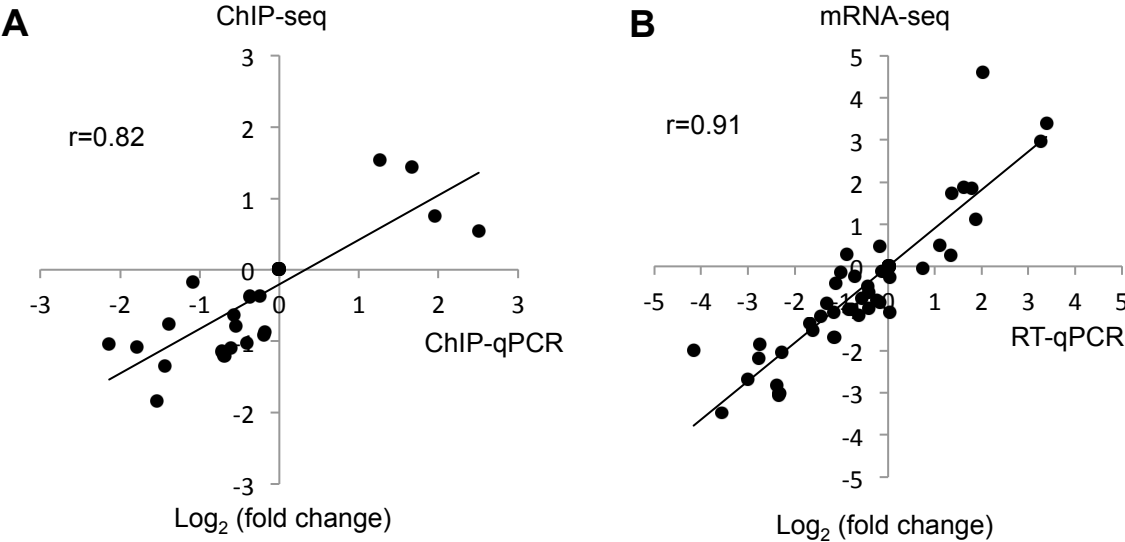

Supplement: Figure S3 — Validation of TR4 ChIP-seq and RNA-seq data. (A) TR4 binding detected by ChIP-seq was validated by ChIP-qPCR. (B) Gene expression detected by RNA-seq was validated using RT-qPCR. The relative fold change in TR4 enrichment or the gene expression in all assays was normalized to that of day 8 erythroid cells and the data are presented as binary logarithms (log2). The Pearson's correlation coefficient, r, for each data set is indicated. (PDF) [file pgen.1004339.s003.pdf]

Supplemental Figure 4

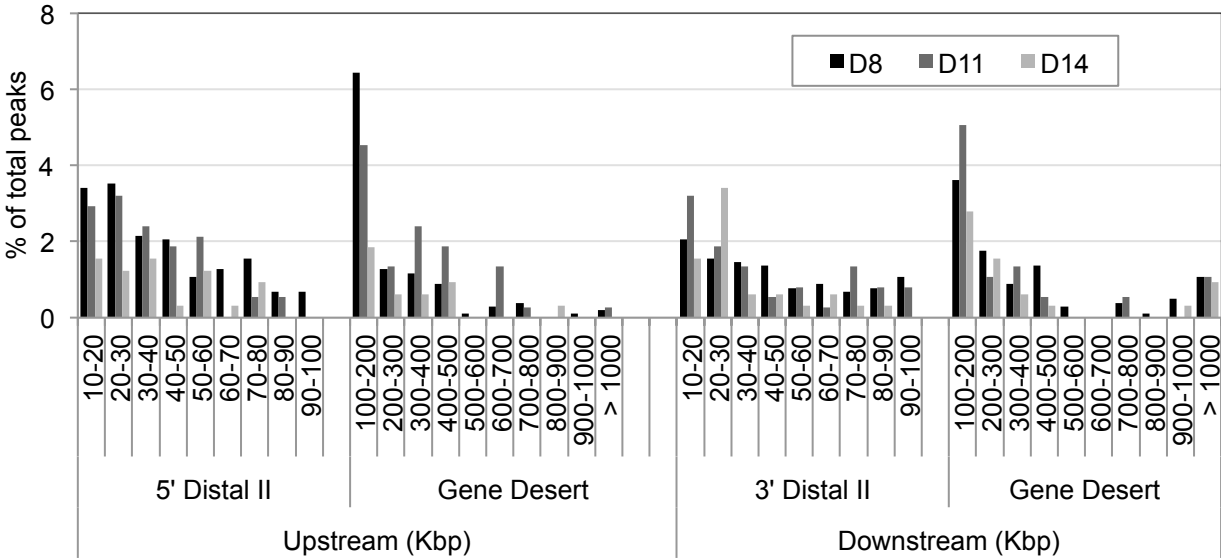

Supplement: Figure S4 — Distribution of peaks located >10 Kbp from the nearest genes. Peaks located 10–100 Kbp upstream (5′ distal II), 10–100 Kbp downstream (3′ distal II) or >100 Kbp either 5′ or 3′ from the nearest genes (gene desert) are graphed. The bin size is 10 Kbp for 5′ distal II and 3′ distal II and 100 Kbp for the gene deserts. (PDF) [file pgen.1004339.s004.pdf]

Supplemental Figure 5

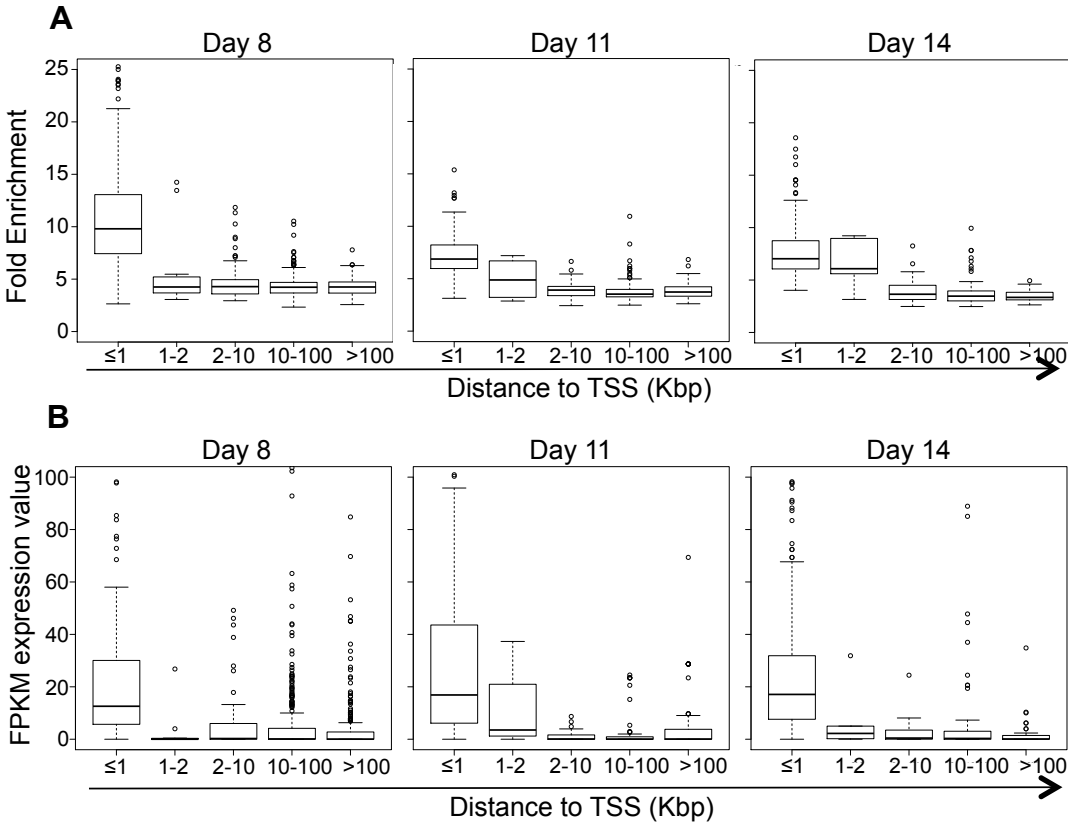

Supplement: Figure S5 — TR4 peaks close to TSSs are more enriched and are associated with higher gene transcription levels. Box-and-whisker diagrams illustrating the correlation of peak enrichment (A) or gene expression (B) given the distance (from peak center) to the nearest TSS in day 8, 11 and 14 erythroid cells. The gene expression profile was generated by RNA-seq and measured as FPKM. (PDF) [file pgen.1004339.s005.pdf]

Supplemental Figure 6

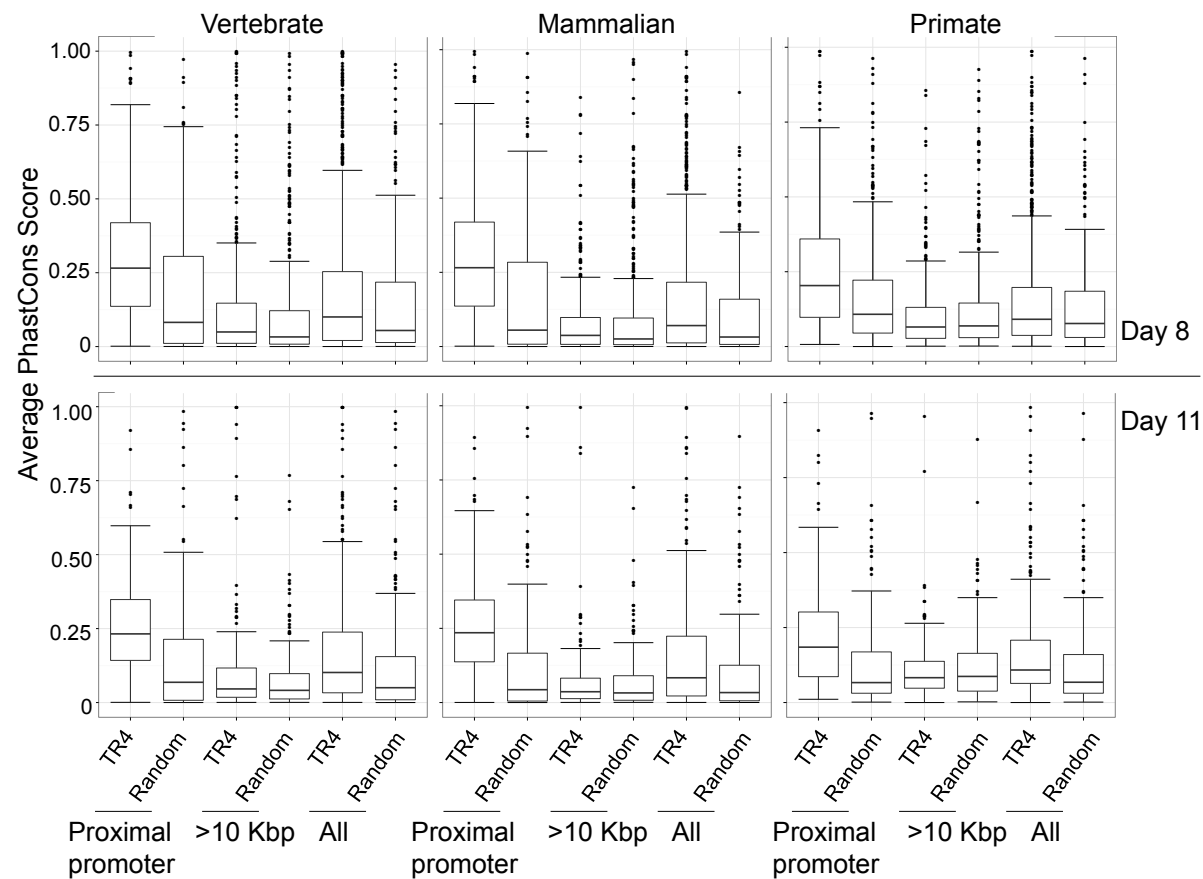

Supplement: Figure S6 — Comparison of the average PhastCons scores of TR4 peaks and random control sequences among the peaks located at the proximal promoter, >10 Kbp from genes or of all identified peaks at day 8 (upper panels) or day 11 (lower panels) of differentiation. (PDF) [file pgen.1004339.s006.pdf]
