# Supplementary material for: Biased, Non-equivalent Gene-Proximal and -Distal Binding Motifs of Orphan Nuclear Receptor TR4 in Primary Human Erythroid Cells
Source: PLoS Genet. 2014 May 8;10(5):e1004339. doi: 10.1371/journal.pgen.1004339 (PMC4014424; doi:10.1371/journal.pgen.1004339)
Supplement: Table S1 — Differentially expressed genes in differentiated CD34+ cells after TR4 depletion by two different shRNA-encoding lentiviruses (sh#174 and sh#658; Materials and Methods). (PDF) [file pgen.1004339.s007.pdf]

DIFFERENTIALLY EXPRESSED GENES FOLLOWING shRNA-MEDIATED TR4 KNOCKDOWN

Gene_Name	FPKM_Control	FPKM_Sh#174	FPKM_Sh#658	FoldChange.Sh#174	FoldChange.Sh#658
A1BG	0.1	0.5	0.6	5.6	6.7
A1BG-AS1	0.0	0.1	0.2	6.9	11.5
A2M	0.1	0.6	0.3	8.3	4.5
AADAC	0.3	0.0	0.1	0.1	0.3
AATK	0.1	0.1	0.1	2.3	2.0
ABCA1	0.3	0.0	0.0	0.1	0.1
ABCA2	0.9	2.4	2.6	2.8	3.0
ABCA3	0.1	0.4	0.5	3.9	4.7
ABCB10	83.4	41.6	39.6	0.5	0.5
ABCB1	0.1	1.1	1.0	13.2	12.0
ABCC13	0.1	0.0	0.0	0.2	0.1
ABCC5-AS1	0.2	0.1	0.0	0.3	#NAME?
ABCC6	0.1	0.2	0.4	4.3	8.2
ABCC9	0.6	1.7	1.5	2.9	2.5
ABCG2	4.7	1.2	1.3	0.3	0.3
ABHD12B	0.0	0.1	0.0	Inf	Inf
ABLIM1	4.0	15.2	17.2	3.8	4.3
ABR	1.4	4.8	5.6	3.4	3.9
ABTB2	0.3	0.6	0.8	2.2	3.1
ACCSL	0.0	0.0	0.2	Inf	Inf
ACE	0.2	0.9	0.9	4.3	4.3
ACHE	12.2	2.3	2.9	0.2	0.2
ACPL2	0.3	0.1	0.1	0.3	0.3
ACPP	0.6	0.1	0.1	0.1	0.1
ACSL6	0.3	0.0	0.0	0.2	0.1
ACSM3	4.5	13.1	12.4	2.9	2.8
ACTL8	0.1	0.1	0.3	2.5	5.2
ADAM10	8.4	20.3	18.8	2.4	2.2
ADAM20P1	0.0	0.1	0.2	4.5	10.0
ADAM22	0.3	0.7	0.6	2.6	2.2
ADAM28	0.2	1.5	1.1	7.6	5.5
ADAM32	0.2	0.4	0.4	2.3	2.1
ADAMDEC1	0.0	0.1	0.0	Inf	Inf
ADAMTS10	0.4	1.0	1.1	2.7	3.1
ADAMTS1	0.0	0.1	0.1	2.8	6.7
ADAP1	0.8	2.4	2.7	3.0	3.3
ADARB1	3.3	6.9	7.1	2.1	2.2
ADCY1	0.0	0.1	0.2	3.3	4.7
ADCY6	2.2	5.7	5.9	2.6	2.7
ADCY9	0.1	0.3	0.2	4.0	2.3
ADCYAP1	0.2	0.9	0.9	4.5	4.4
ADD1	73.0	33.5	36.3	0.5	0.5
ADD2	130.8	54.8	63.1	0.4	0.5
ADD3	11.3	5.2	5.4	0.5	0.5

ADM2	0.4	0.1	0.1	0.2	0.3
ADM	0.9	0.0	0.0	#NAME?	#NAME?
ADORA3	0.1	0.6	0.5	5.3	4.1
ADRA2A	0.6	3.0	3.1	5.0	5.1
AEBP1	0.1	0.2	0.2	3.5	3.4
AFF2	0.0	0.2	0.2	5.8	5.7
AFF3	0.1	0.0	0.0	0.3	0.3
AGAP1	0.8	0.3	0.3	0.5	0.4
AGFG2	16.8	5.6	6.6	0.3	0.4
AGPAT4	0.6	1.2	1.6	2.1	2.7
AGPHD1	0.1	0.3	0.4	3.0	5.1
AHCTF1P1	0.1	0.3	0.3	2.4	2.4
AHCYL2	1.5	3.5	3.2	2.4	2.2
AHRR	0.8	3.3	4.1	4.0	4.9
AHSP	1538.0	583.6	627.8	0.4	0.4
AIF1	1.5	15.6	13.7	10.5	9.2
AIF1L	0.1	0.7	0.4	11.6	7.2
AIFM2	18.0	6.7	7.6	0.4	0.4
AIM1	4.0	1.4	1.4	0.4	0.4
AJUBA	0.1	0.2	0.3	2.5	3.0
AK1	31.6	11.0	11.4	0.3	0.4
AK4	2.5	8.1	7.3	3.2	2.9
AK8	0.3	0.7	0.6	2.6	2.5
AKAP11	3.3	6.9	7.0	2.1	2.2
AKAP12	0.3	1.5	1.1	4.5	3.4
AKAP4	0.2	1.0	1.3	6.4	8.8
AKAP9	1.8	5.7	5.5	3.2	3.1
AKNA	0.5	1.1	1.1	2.2	2.2
AKR1C1	14.4	51.4	45.0	3.6	3.1
AKR7A2P1	0.4	0.1	0.1	0.3	0.2
AKT3	0.2	2.3	2.0	9.3	8.3
ALAD	110.8	24.4	25.9	0.2	0.2
ALAS2	262.3	72.6	90.8	0.3	0.3
ALDH2	0.7	2.4	2.3	3.2	3.0
ALG1L2	0.2	0.4	0.4	2.1	2.0
ALOX5	9.5	3.5	3.1	0.4	0.3
ALOX5AP	2.4	13.0	9.7	5.4	4.0
ALPL	2.9	0.6	0.4	0.2	0.1
ALS2CL	0.0	0.1	0.1	13.4	11.8
ALS2CR12	0.6	0.2	0.2	0.4	0.4
AMH	0.0	0.2	0.2	3.2	4.9
ANGPT1	0.6	4.8	3.3	7.7	5.2
ANKDD1A	0.2	1.0	1.0	4.0	4.0
ANKLE1	37.6	17.1	17.3	0.5	0.5
ANKRD12	2.6	7.8	7.3	3.0	2.8
ANKRD13B	0.2	1.0	1.2	6.1	6.7
ANKRD18A	0.3	0.1	0.1	0.4	0.2

ANKRD20A4	0.1	0.0	0.0	0.2	0.3
ANKRD20A8I	0.3	0.1	0.1	0.4	0.5
ANKRD29	0.0	0.1	0.4	5.5	30.4
ANKRD30BL	1.4	4.4	3.0	3.1	2.1
ANKRD35	0.0	0.1	0.1	15.3	8.4
ANKRD55	0.0	0.1	0.1	4.2	8.4
ANKRD61	2.6	0.7	1.1	0.3	0.4
ANKRD63	0.0	0.1	0.1	4.7	3.1
ANKRD6	0.1	0.3	0.3	4.5	4.8
ANKS6	0.1	0.3	0.3	2.8	3.1
ANPEP	1.3	7.2	7.3	5.4	5.5
ANTXR1	0.0	0.1	0.1	7.5	4.2
ANXA2	46.3	111.4	94.6	2.4	2.0
ANXA2P2	1.7	3.8	4.1	2.3	2.4
ANXA3	0.1	0.4	0.3	3.3	2.3
AOAH	0.1	0.4	0.6	4.7	6.3
AP1S2	2.9	11.1	9.2	3.8	3.2
AP3D1	36.0	15.5	17.7	0.4	0.5
APBA2	1.7	6.1	7.4	3.5	4.3
APC	2.7	7.1	7.3	2.6	2.7
APOA1	0.0	0.2	0.2	Inf	Inf
APOBEC2	0.7	0.4	0.2	0.5	0.3
APP	20.5	48.4	49.0	2.4	2.4
AQP1	5.6	0.9	0.7	0.2	0.1
AQP5	0.0	0.1	0.1	3.4	2.1
ARAP2	0.3	0.7	0.7	2.2	2.1
ARG2	11.6	4.2	4.4	0.4	0.4
ARHGAP22	2.1	6.3	5.5	3.1	2.7
ARHGAP23	16.4	6.4	7.1	0.4	0.4
ARHGAP25	3.5	8.5	9.6	2.4	2.7
ARHGAP26	0.5	1.3	1.4	2.4	2.6
ARHGAP27	0.5	1.2	1.2	2.4	2.4
ARHGAP30	1.6	8.1	8.0	5.1	5.0
ARHGAP31	0.4	0.9	0.9	2.4	2.6
ARHGAP5	2.6	6.8	6.8	2.7	2.7
ARHGAP8	1.4	0.4	0.5	0.3	0.3
ARHGAP9	2.0	5.2	5.3	2.6	2.7
ARHGEF10	0.2	1.0	1.0	4.5	4.4
ARHGEF12	16.4	7.0	7.2	0.4	0.4
ARHGEF17	0.0	0.3	0.4	18.2	19.9
ARHGEF26	0.0	0.1	0.1	6.3	5.6
ARHGEF35	0.2	0.1	0.1	0.5	0.3
ARHGEF3	0.6	1.5	1.8	2.4	2.8
ARHGEF40	0.2	0.8	0.9	3.6	3.8
ARID5B	0.3	0.6	0.8	2.4	2.8
ARL10	0.1	0.3	0.6	5.2	10.7
ARL11	0.1	0.4	0.5	2.9	3.5

ARL13A	0.1	0.0	0.0	0.2	0.4
ARMCX1	0.3	1.2	1.0	3.8	3.1
ARMCX5-GPI	0.1	0.4	0.4	2.8	2.6
ARRDC3	12.3	4.5	5.1	0.4	0.4
ARSD	0.2	1.0	1.2	4.5	5.5
ART4	4.1	0.2	0.6	0.1	0.1
AS3MT	0.5	0.0	0.0	0.0	0.0
ASAP2	0.5	1.2	1.3	2.5	2.7
ASB9P1	0.1	0.1	0.1	2.3	2.1
ASCL2	0.0	0.1	0.1	8.3	5.2
ASIC1	0.0	0.1	0.1	Inf	Inf
ASNS	59.8	21.0	19.2	0.4	0.3
ASPDH	0.0	0.2	0.1	Inf	Inf
ASPH	3.9	11.4	9.8	2.9	2.5
ASPM	7.9	17.4	17.3	2.2	2.2
ASS1	1.0	0.2	0.2	0.2	0.2
ATF3	4.1	1.1	0.6	0.3	0.1
ATG4D	16.6	6.2	7.0	0.4	0.4
ATHL1	1.0	2.7	2.6	2.6	2.5
ATP10D	0.9	2.3	2.0	2.4	2.1
ATP1A3	0.1	0.5	0.4	7.0	5.9
ATP1B2	8.2	3.1	3.3	0.4	0.4
ATP1B4	0.4	2.1	2.1	5.8	5.8
ATP2A3	7.0	20.4	22.2	2.9	3.2
ATP6V0D2	0.0	0.2	0.1	17.3	6.3
ATP6V1G2	0.1	0.6	0.5	5.3	4.1
ATP8A1	0.9	2.1	1.9	2.2	2.1
ATP8A2	0.0	0.1	0.1	20.3	9.5
ATP8B2	3.7	9.9	11.2	2.7	3.0
ATP8B4	0.7	1.7	1.4	2.5	2.1
ATRX	2.6	9.0	8.9	3.5	3.4
ATXN1	0.3	0.9	1.0	2.6	3.1
AUTS2	0.5	1.2	2.3	2.3	4.3
AVP	0.0	0.3	0.1	Inf	Inf
AVPR2	0.0	0.4	0.4	Inf	Inf
AZGP1	0.8	0.2	0.2	0.2	0.2
AZU1	1.4	4.4	4.6	3.2	3.3
B3GNT1	3.3	1.3	1.5	0.4	0.5
BACH2	0.0	0.1	0.1	3.5	3.3
BAIAP2-AS1	0.6	1.4	1.4	2.2	2.2
BANK1	0.5	2.0	2.7	3.9	5.4
BASP1	0.3	1.4	1.1	4.9	3.9
BAZ2B	2.1	4.6	4.7	2.2	2.2
BCAS4	0.3	0.7	0.9	2.2	3.2
BCL2A1	0.0	0.3	0.2	Inf	Inf
BCL6	0.9	0.1	0.1	0.1	0.1
BCRP3	0.2	0.6	0.7	2.7	3.2

BDNF-AS	0.1	0.5	0.4	4.0	3.6
BDNF	0.1	0.5	0.6	3.7	4.4
BDP1	3.6	8.3	8.1	2.3	2.2
BEGAIN	0.4	1.1	1.0	2.5	2.2
BEND4	0.0	0.5	0.5	17.3	18.8
BEND6	0.0	0.1	0.2	3.2	6.3
BEST3	0.2	0.0	0.0	0.1	0.3
BHLHE40-AS1	0.0	0.1	0.2	Inf	Inf
BIN1	0.2	0.5	0.7	3.0	4.5
BLVRB	899.3	318.2	378.3	0.4	0.4
BMX	0.2	0.6	0.6	2.5	2.8
BOD1L1	4.3	9.6	9.2	2.2	2.1
BRCA2	2.1	4.8	4.9	2.3	2.4
BRDT	0.0	0.1	0.0	13.2	4.2
BRSK1	0.0	0.1	0.1	6.3	7.4
BST1	0.3	1.6	1.2	4.7	3.7
BTBD19	0.4	0.9	0.9	2.2	2.0
BTC	0.0	0.2	0.1	6.1	4.1
BTG2	32.0	11.2	10.8	0.4	0.3
C10orf118	0.6	2.6	2.7	4.2	4.4
C10orf25	0.0	0.1	0.1	4.4	3.5
C10orf32-AS1	0.0	0.4	0.3	3053.3	2434.1
C10orf68	0.1	0.4	0.3	2.9	2.4
C11orf84	2.8	6.3	5.8	2.3	2.1
C12orf69	0.1	0.0	0.0	#NAME?	0.3
C12orf71	0.0	0.2	0.2	Inf	Inf
C12orf76	2.0	4.5	5.5	2.2	2.7
C15orf52	0.2	0.4	0.5	2.2	2.8
C17orf50	0.0	0.1	0.3	3.4	9.2
C17orf51	0.1	0.0	0.1	0.2	0.4
C17orf61-PLS	0.9	0.0	0.0	0.0	0.0
C17orf67	0.2	1.0	0.8	5.6	4.4
C17orf99	2.4	1.0	0.8	0.4	0.3
C19orf33	0.9	2.4	2.0	2.7	2.2
C19orf35	0.2	0.7	0.4	3.3	2.0
C19orf38	0.2	0.4	1.0	2.6	6.4
C19orf59	0.0	0.4	0.4	15.6	14.5
C19orf77	0.2	1.3	1.6	8.3	10.5
C1orf114	0.1	0.4	0.2	5.7	2.6
C1orf115	0.0	0.5	0.3	22.6	13.1
C1orf145	0.1	0.6	0.2	11.0	3.3
C1orf151-NB	0.5	3.1	2.4	5.9	4.6
C1orf162	0.8	2.9	2.5	3.5	3.1
C1orf186	8.4	3.9	3.0	0.5	0.4
C1orf227	0.2	0.0	0.0	#NAME?	#NAME?
C1orf228	0.4	1.0	0.8	2.6	2.0
C1QA	0.0	0.1	0.0	Inf	Inf

C1QB	0.0	0.3	0.1	Inf	Inf
C1QC	0.0	0.3	0.3	Inf	Inf
C1QL3	0.3	0.7	0.8	2.3	2.8
C1QTNF9B	0.1	0.4	0.6	2.5	4.1
C20orf197	0.7	4.8	4.0	6.6	5.5
C20orf203	0.1	0.3	0.3	3.8	4.1
C21orf91	1.6	5.2	5.5	3.3	3.4
C2CD4B	0.3	0.0	0.0	0.1	#NAME?
C2orf70	0.0	0.1	0.4	Inf	Inf
C2orf81	0.0	0.2	0.1	8.6	4.7
C2orf82	0.3	0.1	0.0	0.2	#NAME?
C2orf83	0.0	0.1	0.2	3.1	5.2
C3	0.0	0.5	0.4	9.8	9.6
C3orf14	2.3	6.2	7.0	2.6	3.0
C3orf27	0.0	0.1	0.3	4.9	12.1
C3orf55	0.1	0.3	0.5	2.7	4.1
C3orf80	1.6	5.2	4.0	3.3	2.5
C4orf26	0.2	0.0	0.1	0.1	0.5
C5	0.2	0.5	0.5	2.2	2.1
C5orf4	31.9	14.6	14.5	0.5	0.5
C6orf165	0.0	0.1	0.1	2.3	2.4
C6orf25	4.9	16.0	15.8	3.3	3.3
C7orf53	0.2	0.5	0.6	2.7	2.8
C9orf117	0.1	0.2	0.4	3.0	7.3
C9orf131	0.1	0.3	0.2	4.6	3.3
C9orf139	0.1	0.3	0.2	4.8	3.2
C9orf171	0.2	0.0	0.1	#NAME?	0.4
C9orf24	0.1	0.3	0.3	6.0	5.5
C9orf89	9.4	19.0	21.1	2.0	2.3
C9orf91	0.8	0.3	0.4	0.4	0.5
CA11	1.5	3.4	3.7	2.2	2.4
CA1	490.7	42.0	35.3	0.1	0.1
CA2	270.7	56.3	48.6	0.2	0.2
CA3	2.1	0.6	0.6	0.3	0.3
CABLES1	3.3	7.4	6.9	2.2	2.1
CABP5	0.1	0.0	0.0	0.3	#NAME?
CABP7	0.1	0.8	0.8	8.1	7.8
CABYR	0.0	0.1	0.1	2.7	3.4
CACNA2D2	0.1	0.2	0.3	2.5	3.8
CACNB3	0.3	1.3	1.8	4.2	5.8
CACNB4	0.0	0.1	0.1	5.3	5.5
CAHM	0.3	1.1	1.0	3.7	3.6
CALB1	19.3	45.3	46.6	2.3	2.4
CALB2	0.8	3.7	3.2	4.4	3.9
CALD1	0.2	0.4	0.4	2.2	2.2
CAMKK1	1.4	3.6	4.1	2.5	2.9
CAPSL	0.2	0.9	1.1	4.0	5.3

CARD10	0.3	0.9	1.0	3.6	3.7
CARD11	2.4	10.2	11.6	4.2	4.8
CASP1	0.3	1.7	1.6	5.3	5.0
CAV1	0.3	2.7	2.4	9.7	8.5
CBFB	8.8	22.2	18.0	2.5	2.0
CBR3	0.0	0.1	0.4	Inf	Inf
CBS	15.4	6.0	5.1	0.4	0.3
CBWD6	0.1	0.7	0.9	8.8	12.0
CBX7	5.2	2.1	2.4	0.4	0.5
CCBP2	0.2	1.0	1.3	4.1	5.3
CCDC102A	0.1	0.5	0.4	5.7	4.3
CCDC103	0.2	0.8	0.6	3.3	2.3
CCDC13	0.1	0.4	0.3	3.7	2.2
CCDC146	0.1	0.6	0.5	4.9	3.8
CCDC152	0.3	0.7	0.8	2.2	2.2
CCDC153	0.2	0.0	0.0	0.2	#NAME?
CCDC171	0.4	1.3	1.4	3.1	3.3
CCDC173	0.1	0.8	0.8	6.1	6.4
CCDC175	0.0	0.2	0.1	6.2	2.8
CCDC18	2.2	6.3	6.5	2.8	2.9
CCDC19	0.3	0.7	0.8	2.3	2.4
CCDC39	0.3	1.2	1.1	3.9	3.5
CCDC54	0.1	0.0	0.1	#NAME?	0.4
CCDC62	0.0	0.1	0.1	6.8	3.4
CCDC64	0.0	0.2	0.5	5.4	12.1
CCDC65	0.1	0.3	0.4	4.5	5.2
CCDC74A	0.1	0.5	0.8	6.9	11.4
CCDC78	1.0	2.8	2.5	2.7	2.4
CCDC88A	4.8	12.7	12.0	2.6	2.5
CCL16	0.2	0.0	0.0	0.3	0.3
CCL1	1.5	5.7	6.0	3.8	4.0
CCL27	0.1	0.0	0.0	#NAME?	#NAME?
CCL2	0.2	0.6	1.1	3.0	5.3
CCL3	0.0	0.3	0.8	5.9	16.2
CCND1	1.8	5.7	5.1	3.3	2.9
CCNJL	0.1	0.6	0.6	9.6	9.5
CCR10	0.4	0.1	0.1	0.3	0.2
CCR1	0.0	0.3	0.2	7.3	3.7
CCT6B	0.7	1.4	1.5	2.1	2.2
CD109	0.9	2.6	2.7	2.9	3.0
CD14	0.1	0.3	0.3	4.7	5.5
CD163	0.0	0.2	0.0	Inf	Inf
CD1D	0.3	0.1	0.1	0.3	0.5
CD200R1	0.1	0.2	0.3	3.2	5.0
CD200R1L	0.1	0.4	0.5	4.9	6.4
CD226	0.5	1.6	1.1	3.3	2.2
CD27	0.3	1.2	0.7	3.8	2.2

CD300A	0.6	2.4	2.2	3.8	3.5
CD300LF	0.2	0.6	0.8	3.2	4.5
CD34	0.2	2.0	2.0	13.3	13.0
CD38	0.1	3.9	2.9	59.2	43.6
CD3D	0.0	0.4	0.1	Inf	Inf
CD3G	0.0	0.1	0.1	4.1	4.1
CD48	0.2	1.3	1.5	8.7	9.7
CD4	2.8	7.9	7.6	2.8	2.7
CD68	12.6	29.2	31.7	2.3	2.5
CD69	4.7	11.0	10.9	2.4	2.3
CD72	0.1	0.2	0.3	2.1	2.5
CD74	10.0	90.6	90.9	9.0	9.1
CD79A	0.1	0.3	0.3	2.4	2.3
CD79B	0.1	0.5	0.8	4.2	7.1
CD7	0.0	0.2	0.2	Inf	Inf
CD96	0.5	4.4	4.9	9.0	10.0
CD9	12.9	48.7	46.8	3.8	3.6
CDC42EP3	1.2	2.9	2.7	2.4	2.3
CDH12	0.0	0.2	0.2	4.7	5.9
CDH1	36.8	16.3	17.2	0.4	0.5
CDHR1	0.1	1.0	1.0	9.3	9.1
CDHR4	0.0	0.1	0.1	3.0	2.4
CDK18	0.1	0.3	0.4	3.4	3.7
CDKN1C	1.8	7.8	7.8	4.3	4.3
CDRT4	0.0	0.3	0.9	2049.3	6278.2
CDSN	1.6	0.5	0.6	0.3	0.4
CEACAM4	0.0	0.1	0.1	2.7	5.2
CEBPA	0.4	2.3	2.1	5.7	5.2
CEBPD	1.0	0.4	0.3	0.4	0.3
CECR1	0.1	0.8	0.7	6.0	5.3
CECR5	1.7	4.6	4.6	2.7	2.6
CELA2A	6.5	20.4	16.4	3.1	2.5
CELA2B	6.4	23.9	24.3	3.8	3.8
CELF2	9.6	25.0	24.0	2.6	2.5
CELF3	0.2	0.0	0.0	0.0	0.1
CELF6	0.3	0.8	0.7	2.9	2.6
CELSR1	0.1	0.2	0.1	2.4	2.0
CENPE	4.5	10.3	9.5	2.3	2.1
CENPF	13.5	32.3	32.7	2.4	2.4
CEP170B	0.1	0.4	0.4	4.4	4.5
CEP290	1.2	3.9	3.3	3.3	2.8
CEP70	4.2	10.4	8.5	2.5	2.0
CERCAM	0.2	0.9	1.3	4.0	5.6
CERK	1.1	3.0	3.0	2.8	2.7
CERS1	0.0	0.1	0.1	Inf	Inf
CERS4	1.6	5.2	4.0	3.3	2.5
CES3	0.3	0.7	1.0	2.2	3.1

CFH	0.1	1.1	1.6	12.4	18.4
CFHR1	0.0	0.1	0.1	Inf	Inf
CFLAR-AS1	0.0	0.1	0.2	2.0	6.2
CGREF1	3.5	8.6	8.4	2.4	2.4
CHAC1	5.0	0.6	0.4	0.1	0.1
CHD9	3.3	8.6	9.0	2.6	2.7
CHDH	0.0	0.1	0.1	2.6	2.3
CHI3L1	0.0	0.0	0.2	Inf	Inf
CHIC1	0.6	2.1	2.0	3.5	3.2
CHM	1.3	2.8	2.7	2.1	2.0
CHML	2.2	4.6	4.5	2.1	2.0
CHPF	0.6	2.1	1.9	3.5	3.1
CHRFAM7A	0.2	0.4	0.4	2.5	2.7
CHRNA6	0.0	0.4	0.3	11.1	8.2
CHRNA7	0.1	0.3	0.3	2.9	2.6
CHST13	0.8	1.8	2.5	2.1	2.9
CHST7	0.1	0.4	0.7	2.7	4.9
CHSY3	0.0	0.1	0.1	3.5	6.3
CIITA	0.1	1.1	1.3	15.2	18.6
CITED2	131.9	61.6	60.4	0.5	0.5
CKAP4	0.7	2.4	2.1	3.3	2.9
CLC	70.7	329.1	275.5	4.7	3.9
CLCNKA	0.0	0.1	0.2	Inf	Inf
CLCNKB	0.2	1.0	1.1	4.9	5.5
CLDN11	0.9	0.0	0.1	0.0	0.1
CLDN14	0.0	0.1	0.3	4.7	10.4
CLDN16	0.0	0.1	0.1	5.1	4.6
CLDN3	0.8	0.1	0.3	0.1	0.3
CLDN4	0.2	0.1	0.0	0.3	0.1
CLDN7	0.9	0.1	0.4	0.2	0.5
CLDND2	0.2	0.6	0.5	3.4	2.9
CLEC12A	0.3	1.1	1.1	4.2	4.5
CLEC12B	0.0	0.2	0.2	Inf	Inf
CLEC2B	2.2	8.1	6.1	3.7	2.8
CLEC3B	0.0	0.3	0.4	Inf	Inf
CLEC5A	0.0	0.2	0.1	20.2	10.5
CLSTN1	3.8	8.3	8.4	2.2	2.2
CLSTN3	2.9	6.0	6.6	2.1	2.3
CLU	5.1	35.7	34.9	7.0	6.8
CMKLR1	0.1	0.4	0.2	4.2	2.2
CMTM3	2.3	11.9	12.5	5.1	5.4
CMTM5	2.8	9.9	10.5	3.6	3.8
CMTM6	10.8	32.5	27.4	3.0	2.5
CNFN	0.2	1.0	1.8	6.0	10.8
CNGA1	0.0	0.1	0.1	Inf	Inf
CNGA4	0.0	0.0	0.1	Inf	Inf
CNKSR2	0.0	0.2	0.1	4.3	2.8

CNN1	0.1	0.0	0.0	#NAME?	0.2
CNNM1	0.2	0.5	0.7	2.6	3.5
CNR2	0.1	0.3	0.4	3.1	3.3
CNTD2	0.0	0.1	0.1	Inf	Inf
CNTNAP1	0.5	0.2	0.2	0.3	0.4
CNTRL	4.5	9.3	9.4	2.1	2.1
COL18A1-AS:	0.2	0.0	0.0	0.3	#NAME?
COL6A1	0.0	0.2	0.1	21.6	20.0
COL6A3	0.1	0.2	0.2	2.1	2.1
COL6A5	0.6	1.3	1.5	2.2	2.5
COL6A6	0.1	0.1	0.2	2.6	4.4
COLEC12	0.0	0.1	0.2	3.1	9.5
COLQ	1.9	5.3	5.7	2.8	3.0
COMMD3-BN	3.4	7.4	8.0	2.2	2.3
COMTD1	11.7	5.7	4.8	0.5	0.4
CORO1A	4.1	11.3	11.9	2.7	2.9
CORO7-PAM	0.3	0.0	0.0	0.0	0.0
COTL1	2.8	11.1	14.1	4.0	5.1
CPA3	8.6	33.5	25.3	3.9	3.0
CPB2	0.0	0.1	0.2	Inf	Inf
CPEB4	14.9	6.7	6.6	0.4	0.4
CPNE2	0.1	0.4	0.6	5.9	8.2
CPNE5	0.1	0.2	0.2	3.9	4.4
CPNE7	0.0	0.6	0.6	Inf	Inf
CPNE8	2.0	5.9	5.3	3.0	2.7
CPS1	0.2	0.6	0.7	2.5	3.2
CPSF4L	0.1	0.0	0.0	#NAME?	#NAME?
CPXM1	0.4	5.3	5.8	14.6	15.9
CR1L	7.7	2.1	2.3	0.3	0.3
CREB3L1	2.2	0.9	0.9	0.4	0.4
CREB3L3	0.5	1.3	1.4	2.6	2.9
CRIM1	5.9	2.8	2.7	0.5	0.5
CRIP1	4.5	20.4	19.8	4.5	4.4
CRIP2	0.4	0.1	0.2	0.3	0.5
CRISPLD1	0.4	2.4	2.1	5.3	4.8
CRMP1	0.0	0.1	0.1	5.2	3.2
CRSP8P	0.0	0.0	0.1	2.0	5.2
CRYBG3	0.0	0.2	0.1	11.8	4.2
CRYGD	0.2	1.4	1.0	6.5	4.5
CRYM-AS1	0.0	0.1	0.3	Inf	Inf
CSF3R	0.1	0.9	0.5	14.8	8.6
CSRP3	0.1	1.0	0.5	7.6	3.5
CST7	32.7	70.7	70.9	2.2	2.2
CSTA	0.0	0.4	0.2	9.3	5.1
CTDSPL	3.5	9.9	9.7	2.8	2.8
CTH	3.9	1.5	1.7	0.4	0.4
CTSE	2.7	0.2	0.2	0.1	0.1

CTSG	0.3	3.7	2.8	10.8	7.9
CTSH	4.1	11.3	11.6	2.7	2.8
CTSW	6.1	23.4	23.5	3.9	3.9
CTTNBP2	0.9	2.4	2.1	2.6	2.2
CTTNBP2NL	0.3	0.6	0.6	2.1	2.2
CTXN1	1.2	2.7	3.7	2.2	3.0
CX3CR1	0.0	0.5	0.5	9.2	9.5
CXADR	1.0	2.2	2.9	2.2	3.0
CXCL1	0.1	0.4	0.3	2.5	2.1
CXCL5	0.0	0.2	0.1	6.9	4.7
CXCR2	0.0	0.1	0.1	2.1	2.7
CXCR2P1	0.2	0.4	0.5	2.2	3.0
CXCR4	4.6	15.3	14.2	3.4	3.1
CXCR6	0.2	0.0	0.0	0.1	0.2
CYB5A	57.5	28.6	27.7	0.5	0.5
CYBA	4.6	16.1	15.9	3.5	3.4
CYFIP2	1.3	4.7	4.9	3.6	3.8
CYP1B1-AS1	0.0	0.1	0.1	Inf	Inf
CYP1B1	7.4	26.6	28.0	3.6	3.8
CYP27A1	0.0	0.2	0.1	Inf	Inf
CYP2U1	0.0	0.3	0.2	10.1	5.9
CYP3A5	0.2	0.6	0.5	3.0	2.4
CYP4F11	0.2	0.8	0.6	4.4	3.3
CYP4F35P	0.1	0.0	0.0	0.2	0.2
CYP4F3	0.7	2.6	2.4	3.7	3.4
CYP7B1	0.3	1.3	1.2	4.0	3.6
CYSLTR1	0.0	0.4	0.2	8.9	5.7
CYSLTR2	0.1	0.6	0.4	7.2	5.1
CYSTM1	14.2	5.5	5.4	0.4	0.4
CYTH4	0.4	1.0	1.1	2.3	2.5
CYYR1	0.0	0.0	0.1	Inf	Inf
DAB2	0.8	2.4	2.3	3.0	2.9
DACT1	0.2	0.3	0.4	2.0	2.5
DACT3	0.0	0.2	0.3	7.3	12.1
DAPP1	0.9	3.7	3.5	4.0	3.9
DBN1	0.4	3.2	3.5	8.7	9.3
DBNDD1	0.0	0.0	0.2	2.1	12.5
DCAF11	52.4	22.0	25.8	0.4	0.5
DCAF4L1	0.0	0.2	0.1	5.2	3.3
DCLK2	0.0	0.2	0.3	Inf	Inf
DDAH2	5.9	14.0	15.5	2.4	2.7
DDIT3	23.6	11.1	8.8	0.5	0.4
DDIT4	132.2	28.7	31.8	0.2	0.2
DEFB124	0.0	1.5	0.9	Inf	Inf
DENND1C	0.2	1.9	1.3	9.4	6.4
DENND3	1.0	2.6	2.4	2.5	2.4
DENND6B	0.2	1.3	1.3	5.8	5.8

DFNB59	0.3	0.8	0.8	2.9	3.0
DGAT2L6	0.0	0.5	0.3	25.4	14.6
DGKE	1.3	3.5	2.9	2.6	2.1
DHH	0.0	0.1	0.2	2.8	6.3
DHRS13	37.7	15.7	16.5	0.4	0.4
DHRS2	2.1	0.3	0.3	0.1	0.1
DHRS3	1.1	0.2	0.1	0.2	0.1
DHRS9	0.1	0.0	0.0	0.1	0.2
DIAPH3-AS1	3.4	1.2	0.6	0.4	0.2
DIP2A-IT1	0.0	0.1	0.2	Inf	Inf
DIRAS1	0.7	2.2	2.4	3.3	3.6
DISC1	0.1	0.3	0.5	3.1	4.9
DISP1	0.1	0.3	0.3	2.4	2.9
DKK1	0.1	0.1	0.1	2.5	2.8
DLC1	9.3	19.3	19.7	2.1	2.1
DLG3	0.1	0.5	0.2	6.6	2.2
DLGAP1-AS3	0.0	0.1	0.1	Inf	Inf
DLK1	0.0	0.1	0.3	3.1	7.8
DLX1	0.0	0.0	0.1	Inf	Inf
DMXL2	0.2	0.4	0.4	2.4	2.3
DNAI2	0.0	0.2	0.1	4.6	2.1
DNAJB14	3.2	8.6	8.0	2.7	2.5
DNAJB2	8.4	2.8	4.1	0.3	0.5
DNAJC12	3.0	6.3	6.8	2.1	2.3
DNAJC18	0.4	1.0	0.8	2.5	2.1
DNALI1	0.1	0.1	0.2	2.1	3.4
DNM1	0.2	1.8	2.1	10.1	11.8
DNM3	1.0	3.5	3.7	3.4	3.5
DOCK1	0.2	1.1	0.8	4.8	3.6
DOK4	0.2	0.5	0.8	2.1	3.4
DPF3	1.5	0.4	0.6	0.2	0.4
DPP10	0.1	0.3	0.2	4.4	2.8
DPP4	0.1	0.7	0.5	10.1	7.8
DPPA3	0.0	0.0	0.2	Inf	Inf
DPPA4	0.8	2.1	2.0	2.6	2.5
DPYD-AS1	0.5	0.1	0.2	0.2	0.5
DRD4	0.2	0.0	0.1	0.1	0.3
DSCR9	0.1	0.2	0.2	3.2	4.2
DTX3	0.9	2.4	2.4	2.8	2.8
DUOX2	0.1	1.0	0.7	7.3	5.5
DUOXA1	0.0	0.1	0.1	4.1	5.2
DUSP1	36.4	10.3	9.1	0.3	0.2
DUSP4	0.1	0.2	0.3	2.1	3.0
DUSP5P1	0.1	0.3	0.4	2.3	3.1
DYNC2H1	0.1	0.6	0.6	4.8	4.5
DYNLRB2	0.2	0.6	1.1	3.4	6.3
DYNLT3	1.8	6.3	5.8	3.5	3.2

E2F2	19.0	7.3	7.2	0.4	0.4
ECM2	0.0	0.1	0.1	5.4	3.2
ECRP	0.0	0.6	0.1	Inf	Inf
EEA1	1.0	4.0	3.8	4.2	4.0
EEF1DP3	0.0	0.3	0.1	Inf	Inf
EEF1E1-MUT	0.0	1.0	0.0	7944.6	14.0
EFCAB12	1.2	4.6	4.4	3.8	3.7
EFCAB5	0.0	0.1	0.3	2.6	6.9
EFHA2	0.1	0.6	0.6	9.4	10.1
EFHB	0.3	0.9	1.0	3.0	3.6
EFHC2	0.3	1.4	1.3	4.4	4.0
EGF	0.1	0.4	0.4	3.0	2.7
EGFL6	0.0	0.1	0.1	4.2	5.8
EGLN3	1.1	4.1	4.3	3.8	4.0
EGOT	0.0	0.1	0.1	Inf	Inf
EHD3	1.3	5.1	5.3	3.8	4.0
ELANE	0.1	3.6	2.7	47.1	34.7
EMB	0.7	5.5	4.9	8.1	7.2
EMP1	0.0	0.2	0.1	Inf	Inf
EMR4P	0.1	0.4	0.3	2.9	2.4
ENPP2	0.1	0.4	0.5	5.7	6.3
ENPP5	0.1	0.4	0.5	3.9	4.4
ENTHD1	0.6	0.3	0.2	0.4	0.3
ENTPD3	0.0	0.2	0.2	9.4	7.9
EPB42	114.1	24.5	28.9	0.2	0.3
EPGN	0.1	0.3	0.2	6.1	3.8
EPS8L2	0.3	0.7	0.9	2.2	2.9
EPS8L3	0.2	0.0	0.0	0.1	0.3
EQTN	0.1	0.0	0.0	#NAME?	#NAME?
EREG	0.7	2.4	2.0	3.3	2.8
ERG	0.1	0.2	0.2	2.5	2.3
ERMAP	70.9	31.3	30.5	0.4	0.4
ERMN	0.0	0.1	0.2	3.6	5.8
ERO1LB	0.4	0.8	0.8	2.1	2.1
ERRFI1	0.3	0.0	0.1	0.1	0.5
ERV3-1	1.3	3.0	3.1	2.4	2.5
ESCO1	2.7	5.7	5.5	2.1	2.0
ESPNL	0.0	0.2	0.2	9.5	10.2
ETAA1	3.0	6.3	6.4	2.1	2.1
ETS1	0.3	1.7	1.6	5.6	5.2
ETV4	0.4	2.7	3.7	6.0	8.4
ETV6	3.7	7.8	7.6	2.1	2.1
EVA1B	0.3	1.6	2.7	5.5	9.0
EVC	0.1	0.3	0.4	5.3	6.6
EVI2A	0.4	1.0	1.0	2.2	2.3
EVI2B	1.6	4.5	3.9	2.7	2.4
EVPLL	0.0	0.2	0.2	10.3	11.5

EXOC3L2	0.9	2.0	2.1	2.3	2.4
EXOC3L4	0.1	0.1	0.2	2.4	3.1
F12	0.1	0.6	0.5	6.3	5.9
F13A1	3.6	8.2	8.6	2.3	2.4
F2RL1	0.1	0.3	0.3	4.5	4.5
F2RL2	0.2	1.2	1.0	5.2	4.4
FA2H	0.2	0.0	0.0	#NAME?	0.1
FABP3	0.3	1.1	0.5	4.2	2.2
FABP5	27.7	78.9	66.1	2.9	2.4
FAIM2	0.0	0.1	0.1	11.9	10.5
FAIM3	0.1	0.4	0.3	4.2	3.4
FAM101B	6.0	21.3	19.8	3.5	3.3
FAM107B	0.6	2.1	1.6	3.2	2.5
FAM108C1	0.4	1.2	1.0	2.9	2.5
FAM111B	0.0	0.8	0.6	41.8	30.6
FAM117A	80.0	24.7	27.4	0.3	0.3
FAM122C	7.2	3.5	3.1	0.5	0.4
FAM133CP	0.0	0.1	0.1	2.8	3.1
FAM169A	0.1	0.2	0.2	2.8	2.2
FAM171A1	6.1	13.6	14.6	2.2	2.4
FAM174B	0.1	0.5	0.3	8.0	5.9
FAM183B	0.0	0.1	0.2	3.8	5.2
FAM196A	0.0	0.2	0.0	Inf	Inf
FAM198A	0.0	0.3	0.3	9.2	7.1
FAM209B	0.1	0.3	0.2	2.6	2.0
FAM212B	1.0	2.6	2.4	2.6	2.4
FAM213A	1.8	5.9	5.1	3.4	2.9
FAM218A	0.1	0.2	0.4	2.2	5.4
FAM24B-CUZ	0.0	0.0	0.3	9178.2	147365655.5
FAM43A	0.1	0.3	0.3	3.6	3.0
FAM45A	2.3	9.6	7.3	4.1	3.1
FAM49A	0.7	0.3	0.3	0.4	0.5
FAM57B	0.7	0.3	0.2	0.4	0.3
FAM69B	0.8	2.0	1.9	2.4	2.2
FAM81B	0.2	1.2	1.2	5.1	5.2
FAM83A	32.0	2.3	2.2	0.1	0.1
FAM83F	23.9	9.7	11.9	0.4	0.5
FAM90A25P	0.0	0.0	0.2	2.8	14.6
FBLN2	0.1	0.5	0.8	7.2	12.0
FBN1	5.4	2.4	2.7	0.4	0.5
FBXO15	0.1	0.2	0.7	2.4	8.9
FBXO17	0.0	0.0	0.1	Inf	Inf
FBXO41	0.1	0.8	0.8	9.2	8.6
FCER1A	4.3	20.7	14.7	4.9	3.5
FCER1G	3.9	10.0	9.0	2.6	2.3
FCGR2A	3.3	7.3	7.3	2.2	2.2
FCGR2B	0.4	0.9	0.9	2.7	2.4

FCGR2C	0.1	0.6	0.6	8.3	7.5
FCRL2	0.0	0.1	0.1	Inf	Inf
FCRLA	0.4	0.1	0.1	0.3	0.2
FENDRR	0.0	0.1	0.1	3.5	3.2
FERMT1	0.3	1.4	1.9	4.5	6.0
FERMT2	0.0	0.1	0.3	2.9	9.4
FEZ1	0.0	0.1	0.1	8.3	6.3
FFAR3	0.1	0.0	0.0	#NAME?	#NAME?
FGF11	0.2	0.5	0.5	2.8	2.7
FGF17	0.5	3.0	2.3	6.1	4.6
FGF2	0.1	0.5	0.7	6.5	7.9
FGF5	0.1	0.3	0.5	3.4	4.6
FGFR1	0.1	0.6	0.4	4.9	3.5
FGL2	0.0	0.6	0.4	12.1	8.0
FHDC1	16.9	7.5	7.4	0.4	0.4
FJX1	0.2	0.8	0.9	4.8	5.1
FKBP10	0.0	0.3	0.4	23.6	40.9
FLI1	1.4	5.2	5.4	3.8	3.9
FLJ12334	0.1	0.2	0.4	2.1	3.6
FLJ14107	0.3	0.1	0.1	0.2	0.5
FLJ35390	0.5	1.2	1.8	2.4	3.4
FLJ37201	0.0	0.1	0.1	5.9	6.3
FLJ39051	0.5	1.2	1.2	2.3	2.4
FLJ42351	0.0	0.3	0.1	Inf	Inf
FLJ42969	0.0	0.0	0.1	Inf	Inf
FLJ45445	0.0	0.1	0.0	9.1	3.7
FLT3	0.0	0.2	0.1	30.7	16.8
FLVCR2	0.1	0.4	0.5	5.1	5.8
FMN1	0.0	0.1	0.1	6.8	7.7
FMO5	0.7	1.6	1.9	2.4	2.7
FMOD	0.3	1.2	1.1	4.5	4.2
FNBP1	0.6	1.5	1.3	2.4	2.1
FOSB	2.2	0.6	0.5	0.3	0.2
FOS	12.9	4.4	3.4	0.3	0.3
FREM1	0.0	0.1	0.1	3.2	4.1
FRMD3	0.0	0.1	0.1	6.2	2.7
FRMD4B	0.8	1.9	1.9	2.2	2.3
FRRS1L	1.0	0.3	0.4	0.3	0.4
FSCN1	14.8	30.2	32.6	2.0	2.2
FSTL4	0.0	0.1	0.2	6.3	12.0
FUT1	7.0	2.9	3.1	0.4	0.4
FUT8-AS1	0.4	1.0	1.2	2.2	2.7
FYB	0.9	5.5	4.9	5.9	5.2
GABBR1	1.0	2.5	2.5	2.5	2.5
GABRE	0.0	0.5	0.2	9.5	4.6
GALNT12	0.1	0.6	0.6	4.7	4.5
GALNT14	0.3	2.3	2.1	9.1	8.0

GALNT3	0.1	0.6	0.4	8.0	5.4
GALR3	0.0	0.1	0.0	Inf	Inf
GAMT	2.7	7.5	10.3	2.7	3.8
GAPT	0.1	1.5	0.9	13.4	8.1
GAS2	0.0	0.5	0.2	16.5	5.2
GAS6	1.5	3.2	3.2	2.1	2.2
GATA5	1.8	0.7	0.4	0.4	0.2
GATS	1.7	4.5	4.5	2.6	2.6
GBP3	0.3	1.1	1.4	4.4	5.4
GBP4	1.3	5.0	4.6	4.0	3.7
GBP5	0.7	3.1	2.9	4.2	3.9
GCC2	2.4	8.9	8.4	3.6	3.4
GCNT4	0.1	0.3	0.3	2.7	2.6
GDF15	81.0	24.9	29.2	0.3	0.4
GDPD5	15.2	5.6	6.6	0.4	0.4
GEM	3.9	1.5	1.7	0.4	0.4
GFAP	0.0	0.0	0.1	2.8	10.5
GIMAP2	2.0	5.3	5.1	2.7	2.5
GIMAP6	0.1	1.4	1.3	10.4	10.0
GIMAP7	0.0	0.2	0.1	Inf	Inf
GIPC3	0.1	0.3	0.4	2.2	3.0
GJA1	0.0	0.5	0.5	9.3	9.5
GJB5	0.0	0.0	0.1	Inf	Inf
GKN2	0.2	0.0	0.0	0.1	#NAME?
GLB1L2	0.2	1.0	1.0	4.3	4.2
GLOD5	0.1	0.3	0.6	2.6	6.0
GLT25D2	0.1	0.2	0.1	3.6	2.2
GLT8D2	0.1	0.2	0.1	3.1	2.1
GMFG	55.5	159.8	160.9	2.9	2.9
GNAT2	0.0	0.3	0.2	6.1	3.6
GNB5	1.2	3.1	2.8	2.6	2.4
GNG11	3.4	8.2	7.4	2.4	2.2
GNG8	0.0	1.9	3.6	Inf	Inf
GNLY	0.2	2.5	1.7	12.5	8.6
GNRH2	0.1	0.0	0.0	#NAME?	#NAME?
GNRHR	0.0	0.2	0.1	5.3	4.0
GOLGA4	4.8	11.2	9.8	2.3	2.0
GOLGA6L7P	0.0	0.2	0.5	Inf	Inf
GOLGA8B	6.0	16.6	13.6	2.8	2.3
GOLGB1	2.8	9.3	9.7	3.3	3.5
GP1BB	7.1	15.0	17.1	2.1	2.4
GPC2	0.1	0.3	0.3	2.3	2.6
GPM6B	0.2	0.9	0.8	3.9	3.4
GPNMB	1.6	3.9	4.6	2.4	2.9
GPR113	0.1	0.3	0.3	3.4	4.0
GPR114	0.0	0.0	0.1	2.1	6.8
GPR126	0.0	0.1	0.1	2.5	3.6

GPR132	0.1	0.3	0.3	5.4	4.5
GPR137C	0.0	0.2	0.1	10.2	3.5
GPR141	0.0	0.2	0.0	Inf	Inf
GPR157	0.1	0.3	0.1	5.1	2.1
GPR161	0.4	1.0	1.1	2.6	2.9
GPR162	0.0	0.1	0.1	8.4	7.4
GPR173	0.0	0.4	0.4	9.1	7.8
GPR21	0.0	0.1	0.0	Inf	Inf
GPR27	0.2	0.1	0.1	0.3	0.3
GPR35	0.0	0.2	0.4	8.3	18.4
GPR3	0.1	0.7	0.9	5.1	6.5
GPR55	0.0	0.1	0.1	3.3	5.6
GPR63	0.2	0.5	0.6	3.2	3.8
GPR65	0.4	1.4	1.1	4.0	3.1
GPR85	0.1	0.2	0.3	3.4	4.6
GPRASP1	0.1	0.2	0.2	2.6	3.3
GPRASP2	0.1	0.0	0.0	0.0	0.0
GPSM1	0.4	2.7	3.4	7.1	9.0
GPSM3	13.0	29.5	31.9	2.3	2.5
GPX7	1.0	3.3	3.0	3.3	3.0
GRAMD1B	0.0	0.2	0.2	8.3	9.4
GRAMD3	1.0	2.6	2.3	2.5	2.2
GRAP	0.0	0.3	0.6	10.3	18.3
GRASP	0.1	0.3	0.6	2.1	4.2
GRIK4	0.0	0.1	0.4	4.4	13.3
GRIK5	0.2	2.7	2.7	11.8	11.6
GRIN2D	0.1	0.3	0.3	5.5	6.3
GRK5	0.8	2.4	2.2	2.8	2.7
GRM4	0.0	0.1	0.2	3.1	4.7
GSTP1	25.1	66.5	64.1	2.7	2.6
GTF2IP1	0.1	0.5	0.4	4.1	3.7
GTF2IP1	0.1	0.2	0.2	2.6	2.8
GTF2IRD2B	0.1	0.4	0.5	3.0	4.3
GTF2IRD2	0.0	0.2	0.2	12.2	13.7
GUCY1A3	0.5	1.9	2.2	3.8	4.5
GUCY1B3	1.8	5.5	5.7	3.0	3.1
GVINP1	0.1	0.3	0.2	4.0	3.3
GYG2	0.1	0.2	0.2	3.0	2.6
GYG2P1	1.5	0.7	0.5	0.5	0.4
GYPA	87.7	28.5	26.5	0.3	0.3
GYPB	126.8	29.1	31.1	0.2	0.2
GYPE	70.7	21.1	23.1	0.3	0.3
H1F0	195.9	39.9	42.1	0.2	0.2
H2AFY2	2.0	0.8	0.8	0.4	0.4
H3F3C	0.0	0.0	0.1	Inf	Inf
HAGH	67.0	30.3	31.0	0.5	0.5
HAL	0.1	0.4	0.4	3.8	3.2

HAVCR1P1	0.0	0.2	0.2	6.1	8.3
HBA2	1045.7	325.7	372.3	0.3	0.4
HBE1	9.9	96.0	104.2	9.7	10.5
HBM	59.1	3.6	3.5	0.1	0.1
HBZ	3.0	0.9	1.1	0.3	0.4
HCG4B	0.0	0.1	0.0	Inf	Inf
HCP5	0.0	0.1	0.1	3.1	5.2
HCST	3.1	13.5	14.2	4.3	4.5
HDC	11.4	31.2	27.1	2.7	2.4
HDX	0.0	0.2	0.2	3.2	3.9
HEG1	0.1	0.1	0.2	2.4	2.7
HEMGN	104.8	44.4	40.8	0.4	0.4
HEPHL1	0.0	0.0	0.1	4.2	10.6
HES6	174.3	66.0	74.5	0.4	0.4
HEY1	0.7	1.8	1.8	2.8	2.8
HEY2	0.0	0.1	0.1	6.2	11.5
HGD	0.6	1.7	1.7	2.9	2.9
HGF	0.0	0.2	0.2	6.8	5.8
HHEX	13.4	29.4	27.5	2.2	2.1
HIPK2	2.3	5.0	5.9	2.2	2.6
HIST1H1T	0.2	0.0	0.0	#NAME?	#NAME?
HIST1H2BK	16.0	37.0	36.6	2.3	2.3
HIST1H2BL	2.1	0.6	0.5	0.3	0.2
HIST1H2BM	1.6	0.8	0.7	0.5	0.4
HIST1H3A	2.4	0.3	1.1	0.1	0.5
HIST1H4D	2.6	0.7	1.0	0.3	0.4
HIST1H4L	0.9	0.0	0.1	#NAME?	0.2
HIST2H2BC	7.6	3.1	2.7	0.4	0.4
HIST2H2BF	0.0	0.2	0.2	Inf	Inf
HIST3H2A	16.1	7.5	7.0	0.5	0.4
HIST3H2BB	1.6	0.3	0.7	0.2	0.4
HLA-B	22.5	67.5	71.9	3.0	3.2
HLA-DMA	7.4	25.5	19.6	3.4	2.6
HLA-DMB	0.0	0.2	0.3	7.5	14.5
HLA-DOB	0.3	0.6	0.8	2.4	3.2
HLA-DPA1	2.1	17.3	14.8	8.3	7.1
HLA-DPB1	1.2	8.3	9.9	7.2	8.5
HLA-DQB1	0.0	0.2	0.1	12.3	6.2
HLA-DRA	1.5	43.8	42.0	29.0	27.9
HLA-DRB1	1.7	32.8	29.4	19.6	17.6
HLA-DRB5	0.4	6.4	5.7	14.8	13.1
HLA-DRB6	0.1	1.8	2.2	16.6	19.9
HLA-F	0.4	1.7	0.9	4.0	2.2
HM13-AS1	0.2	0.9	0.4	4.7	2.4
HMCN1	0.0	0.1	0.1	2.4	4.1
HMGB3P1	0.2	0.1	0.1	0.4	0.4
HMGCR	25.4	57.0	51.9	2.2	2.0

HMGCS1	100.1	348.3	307.1	3.5	3.1
HMOX1	5.1	11.4	10.4	2.2	2.0
HNF4A	0.2	0.0	0.0	0.2	#NAME?
HNRNPA1P3:	0.0	0.2	0.1	Inf	Inf
HOTAIRM1	0.8	1.7	2.5	2.0	3.1
HOXA10	1.0	4.4	3.6	4.4	3.6
HOXA10-HO)	0.0	0.1	0.0	################	169011347631110.0
HOXA1	0.1	0.2	0.3	2.5	3.7
HOXA5	0.0	0.1	0.1	Inf	Inf
HOXA7	0.0	0.5	0.4	14.8	12.0
HOXA9	0.6	3.2	2.5	5.3	4.0
HOXA-AS4	0.0	0.2	0.2	Inf	Inf
HOXB9	0.1	0.0	0.0	#NAME?	#NAME?
HPN	0.1	0.2	0.2	2.9	3.8
HPSE	1.7	5.2	5.1	3.1	3.0
HRASLS	0.1	0.4	0.3	3.8	3.1
HRH2	0.0	0.3	0.2	24.9	12.6
HR	0.2	0.5	0.7	2.6	3.6
HSD17B14	0.7	1.7	1.6	2.4	2.3
HSD17B7	3.6	10.3	8.8	2.8	2.4
HSD17B7P2	0.7	2.1	1.9	3.1	2.8
HSH2D	2.0	7.1	5.6	3.5	2.8
HSPA1B	15.9	7.1	6.7	0.4	0.4
HSPA7	0.4	1.2	0.8	3.3	2.3
HSPE1-MOB4	0.0	0.1	0.1	################	################
HTR2A	0.0	0.2	0.1	4.5	2.4
HTR3C	0.0	0.1	0.1	3.4	6.2
HTRA1	0.0	0.1	0.1	Inf	Inf
HTRA3	0.0	1.0	1.8	42.3	76.0
HVCN1	0.2	0.6	0.8	3.3	4.0
IAPP	0.0	0.1	0.1	2.8	2.8
ICAM4	45.6	21.5	22.3	0.5	0.5
IFI16	0.8	5.4	4.5	6.4	5.4
IFI27L2	18.6	43.6	54.7	2.3	2.9
IFI44	4.0	9.3	8.4	2.3	2.1
IFITM1	16.1	55.1	66.3	3.4	4.1
IFITM2	54.2	124.9	133.4	2.3	2.5
IFITM3	5.9	21.8	16.8	3.7	2.8
IGF2BP3	0.1	0.6	0.6	4.2	4.4
IGFBP2	8.9	25.1	26.8	2.8	3.0
IGFBP4	2.8	11.1	14.1	3.9	5.0
IGFBP7	4.9	13.5	12.4	2.8	2.5
IGFL2	11.3	2.9	2.4	0.3	0.2
IGLL1	3.1	10.2	10.3	3.3	3.4
IGLL3P	0.6	0.2	0.1	0.3	0.1
IGSF10	0.1	1.2	0.5	8.4	3.8
IGSF1	0.0	0.1	0.0	Inf	Inf

IGSF9B	0.1	0.2	0.2	3.8	3.5
IKZF2	0.2	0.6	0.5	3.1	3.0
IL10	0.1	0.2	0.1	3.2	2.4
IL12A	0.0	0.1	0.1	2.4	2.1
IL15RA	16.5	7.9	7.4	0.5	0.5
IL17D	0.0	0.2	0.3	Inf	Inf
IL1B	0.5	5.2	5.7	9.5	10.5
IL21R-AS1	0.1	0.6	0.7	4.9	5.6
IL21R	0.1	0.7	0.7	11.3	11.0
IL24	0.0	0.3	0.1	15.5	5.0
IL2RG	22.1	49.6	50.7	2.2	2.3
IL32	0.0	0.1	0.1	Inf	Inf
IL4	0.1	0.1	0.1	2.1	2.2
IL5RA	0.0	0.1	0.1	13.2	23.2
IL6ST	2.9	6.4	6.1	2.2	2.1
IL7R	1.3	5.1	5.3	3.9	4.1
IMPA1	3.7	7.8	7.7	2.1	2.1
IMPG2	0.1	0.2	0.1	2.5	2.2
INHA	0.3	0.6	1.2	2.2	4.1
INO80B-WBF	0.0	4.9	0.0	34106.1	2.7
INPP1	3.0	7.3	6.2	2.4	2.0
INPP4B	0.3	1.1	0.9	3.2	2.7
IQSEC2	0.7	1.5	2.5	2.1	3.4
IRAK3	2.7	7.0	6.3	2.6	2.3
IRF5	0.2	1.0	1.1	4.1	4.4
IRX3	0.0	0.2	0.1	13.1	7.3
ISCA2	35.5	12.3	13.2	0.3	0.4
ISM1	0.0	0.1	0.3	3.5	14.2
ITGA10	0.0	0.1	0.1	2.0	2.4
ITGA2B	25.8	76.3	83.3	3.0	3.2
ITGA2	0.1	0.5	0.3	4.7	2.7
ITGA6	1.4	3.7	2.9	2.7	2.1
ITGA8	0.0	0.1	0.2	5.2	8.9
ITGAL	0.2	1.1	1.2	5.0	5.7
ITGAM	0.5	1.3	1.1	2.7	2.4
ITGAX	0.8	2.0	2.0	2.3	2.4
ITGB2-AS1	0.1	0.4	0.2	4.3	2.2
ITGB2	2.0	5.2	5.6	2.6	2.9
ITGB5	1.0	2.0	2.2	2.0	2.2
ITGB7	1.8	3.8	3.8	2.2	2.2
ITGBL1	0.2	0.0	0.1	0.1	0.4
ITPR1	2.1	4.9	5.0	2.3	2.4
JAG2	0.0	0.1	0.2	5.2	7.5
JAK3	0.2	0.5	0.4	2.9	2.4
JAKMIP1	0.1	0.5	0.6	6.2	6.7
JAKMIP2	0.1	2.1	1.9	35.2	30.9
JDP2	0.4	0.2	0.2	0.5	0.5

JHDM1D	3.5	1.6	1.8	0.4	0.5
JMJD1C	3.8	7.9	7.9	2.1	2.1
JMJD7-PLA2(	0.4	1.5	3.0	3.9	7.7
JPH1	0.2	0.6	0.4	3.8	2.4
JPH4	0.0	0.2	0.1	12.6	6.3
JUNB	63.8	21.4	21.6	0.3	0.3
JUN	6.0	2.7	1.0	0.4	0.2
KALRN	0.2	0.8	0.9	3.2	3.7
KAZN	0.5	1.1	1.3	2.2	2.6
KBTBD11	0.0	0.3	0.2	15.9	11.4
KCNAB2	8.6	18.2	21.3	2.1	2.5
KCNC3	0.0	0.1	0.1	3.1	7.4
KCNE4	0.0	0.1	0.1	2.8	6.8
KCNIP2	0.8	2.8	2.0	3.4	2.5
KCNIP4	0.4	0.8	0.8	2.0	2.1
KCNJ12	0.0	0.1	0.1	Inf	Inf
KCNJ18	0.0	0.1	0.1	Inf	Inf
KCNQ1OT1	0.5	1.0	1.1	2.1	2.3
KCNQ5	0.4	1.8	1.4	5.1	3.9
KCNT2	0.0	0.0	0.1	Inf	Inf
KCTD12	0.1	0.3	0.3	3.1	2.3
KCTD17	1.3	4.1	4.8	3.2	3.7
KHDRBS3	0.1	0.3	0.1	4.5	2.1
KIAA0125	2.4	8.8	9.0	3.7	3.8
KIAA1009	1.5	3.5	3.0	2.3	2.0
KIAA1033	3.9	11.1	10.2	2.8	2.6
KIAA1107	0.2	0.5	0.8	2.7	4.5
KIAA1161	0.0	0.1	0.2	2.6	10.2
KIAA1377	0.2	1.2	1.3	5.9	6.4
KIAA1598	0.9	2.6	2.6	3.0	3.0
KIF14	3.9	8.3	7.8	2.1	2.0
KIF20B	7.5	16.6	15.3	2.2	2.1
KIF21B	0.2	0.7	0.9	4.6	6.1
KIF3C	1.1	3.4	3.9	3.2	3.6
KIFC3	5.9	13.0	13.4	2.2	2.3
KLB	0.1	0.1	0.1	2.4	2.6
KLC3	0.1	0.2	0.2	2.6	2.1
KLF12	0.0	0.1	0.2	6.9	9.7
KLF13	55.0	26.5	27.0	0.5	0.5
KLF15	1.5	0.3	0.2	0.2	0.1
KLF8	0.1	0.3	0.5	4.9	7.1
KLHDC7B	0.0	0.1	0.1	5.6	10.5
KLHL13	0.3	0.7	1.0	2.7	3.6
KLHL17	1.5	0.7	0.8	0.5	0.5
KLHL31	0.0	0.2	0.2	6.6	6.1
KLHL35	0.4	0.8	0.9	2.1	2.5
KLHL5	0.8	2.4	2.5	3.1	3.3

KLK1	0.4	1.0	0.9	2.6	2.4
KPNA5	0.8	2.5	3.1	3.0	3.7
KRBA1	0.5	1.1	1.5	2.5	3.3
KREMEN1	24.2	7.3	7.4	0.3	0.3
KREMEN2	0.3	0.1	0.1	0.4	0.4
KRT13	16.1	7.3	6.5	0.5	0.4
KRT19	9.2	2.4	2.4	0.3	0.3
KRT74	0.0	0.1	0.2	2.9	3.4
KRT77	0.0	0.1	0.1	2.8	2.6
KRT79	0.6	5.3	6.1	8.5	9.8
KRT81	0.0	0.1	0.2	8.3	9.4
KTN1	19.7	44.4	40.0	2.3	2.0
LACC1	0.4	1.0	1.1	2.4	2.6
LAIR1	0.6	2.8	2.1	4.7	3.5
LAIR2	0.6	2.4	3.1	4.0	5.3
LAMB2	0.2	0.7	0.5	4.6	3.4
LAMB2P1	0.4	0.1	0.0	0.2	0.1
LAMP3	1.1	0.4	0.5	0.3	0.5
LAPTM4B	12.8	52.6	46.6	4.1	3.6
LAT2	3.2	7.1	6.4	2.2	2.0
LCA5	1.7	4.3	3.7	2.5	2.1
LCN15	0.1	0.0	0.0	0.2	0.3
LCN2	2.9	8.4	10.2	2.9	3.5
LCP2	16.5	48.0	47.3	2.9	2.9
LDB3	0.1	0.0	0.0	0.2	0.1
LDLRAD2	0.1	0.1	0.1	2.1	2.1
LDOC1	0.0	0.6	0.5	23.2	20.7
LEF1-AS1	0.3	1.8	1.2	5.1	3.5
LEF1	1.9	6.3	5.6	3.3	2.9
LEFTY1	0.0	0.1	0.1	3.1	2.6
LFNG	0.4	1.4	1.7	3.7	4.6
LGALSL	2.1	7.5	5.7	3.5	2.7
LGMN	0.4	1.1	0.9	2.7	2.1
LGR4	2.6	0.7	0.9	0.3	0.3
LGR6	0.7	2.8	2.8	3.8	3.8
LHX6	0.0	0.1	0.2	3.8	5.4
LIF	1.8	5.8	5.8	3.2	3.2
LIG4	2.6	5.6	5.4	2.1	2.1
LIMD2	3.5	11.9	11.9	3.4	3.4
LIN7A	0.0	0.2	0.4	Inf	Inf
LINC00028	0.2	0.0	0.1	#NAME?	0.3
LINC00035	0.1	0.4	0.6	4.3	5.8
LINC00085	0.2	1.1	0.7	5.6	3.6
LINC00092	0.2	0.7	0.5	3.2	2.3
LINC00173	0.9	2.6	3.1	3.0	3.6
LINC00441	0.1	0.2	0.3	2.5	3.1
LINC00520	0.2	1.0	0.7	6.0	4.0

LINC00570	0.6	0.3	0.1	0.5	0.1
LINC00571	0.2	0.4	0.4	2.2	2.4
LINC00654	0.1	0.4	0.3	3.9	2.8
LINC00656	0.1	0.0	0.0	#NAME?	0.2
LINC00670	0.0	0.1	0.0	Inf	Inf
LIPC	0.0	0.1	0.2	Inf	Inf
LIPH	0.1	0.5	0.6	4.8	5.8
LMO1	0.0	0.1	0.1	3.4	4.1
LNX1	0.3	1.1	1.0	3.3	3.0
LOC1001278	0.2	0.4	0.5	2.7	2.8
LOC1001282	2.3	5.3	5.7	2.4	2.5
LOC1001290	1.9	0.5	0.5	0.2	0.2
LOC1001300	0.1	0.2	0.3	2.3	2.9
LOC1001302	0.0	0.3	0.4	9.3	11.1
LOC1001302	0.0	0.4	0.3	Inf	Inf
LOC1001309	0.2	0.5	0.6	2.2	2.6
LOC1001317	0.9	0.1	0.0	0.1	0.0
LOC1001327	0.0	0.1	0.1	Inf	Inf
LOC1002707	0.1	0.4	0.4	3.3	3.4
LOC1002717	0.1	0.4	0.4	6.2	6.9
LOC1002722	0.6	1.2	1.3	2.1	2.3
LOC1002871	0.6	1.8	2.1	3.0	3.6
LOC1002873	0.7	1.9	1.5	2.8	2.2
LOC1002884	0.2	0.5	0.9	2.8	4.3
LOC1002889	0.2	0.0	0.1	0.2	0.3
LOC1003026	0.3	0.0	0.0	#NAME?	#NAME?
LOC1004991	0.0	0.1	0.1	2.0	4.1
LOC1004992	0.3	1.0	0.6	3.2	2.2
LOC1005056	0.3	0.1	0.1	0.4	0.3
LOC1005057	0.2	0.5	0.5	3.1	3.1
LOC1005058	0.0	0.1	1.0	2.0	26.5
LOC1005061	0.2	1.4	0.7	7.8	3.6
LOC1005061	0.6	1.5	1.7	2.5	2.7
LOC1005061	0.0	0.4	0.2	Inf	Inf
LOC1005061	0.0	0.1	0.1	2.7	5.2
LOC1005062	0.1	0.0	0.0	0.1	#NAME?
LOC1005062	0.1	0.0	0.0	#NAME?	#NAME?
LOC1005063	0.0	0.1	0.3	4.4	9.9
LOC1005067	0.4	0.9	0.8	2.3	2.2
LOC1005072	0.0	0.1	0.1	3.4	3.1
LOC1005073	0.1	0.6	0.7	5.5	6.3
LOC1005073	0.1	0.0	0.0	0.3	0.2
LOC1005074	0.0	0.2	0.2	4.0	3.4
LOC1005074	0.6	2.4	2.5	4.3	4.5
LOC1006309	0.8	2.2	2.6	2.8	3.4
LOC143666	0.2	0.5	0.5	2.4	2.2
LOC151009	0.1	0.4	0.5	7.2	8.1

LOC154761	0.1	0.3	0.3	2.9	2.3
LOC283174	0.1	0.4	0.3	4.3	3.1
LOC283440	0.0	0.3	0.1	Inf	Inf
LOC283692	0.3	0.7	0.6	2.2	2.0
LOC283710	0.0	0.1	0.1	4.0	2.1
LOC284276	0.1	0.1	0.1	2.2	2.1
LOC284889	0.0	0.3	0.2	6.7	4.5
LOC285540	0.3	1.1	0.9	3.4	3.1
LOC285758	0.0	0.0	0.1	3.5	11.5
LOC286190	0.0	0.0	0.1	2.1	6.3
LOC286367	0.5	1.2	1.4	2.2	2.6
LOC339166	0.0	0.1	0.1	Inf	Inf
LOC344595	1.3	2.7	3.0	2.0	2.2
LOC344887	0.3	2.0	2.8	6.3	8.8
LOC388849	0.0	0.1	0.6	Inf	Inf
LOC388948	0.0	0.0	0.1	Inf	Inf
LOC389033	0.0	0.2	0.1	Inf	Inf
LOC389458	0.5	1.1	1.1	2.2	2.1
LOC400752	0.2	0.6	0.6	2.9	2.8
LOC401134	0.0	0.3	0.2	9.9	5.3
LOC643387	0.2	0.0	0.1	0.3	0.5
LOC644248	0.0	0.0	0.5	Inf	Inf
LOC645638	0.1	0.0	0.0	#NAME?	#NAME?
LOC646214	0.5	2.1	2.2	4.5	4.8
LOC646278	0.4	0.7	1.1	2.1	3.1
LOC646862	0.1	0.0	0.0	#NAME?	#NAME?
LOC654342	0.8	1.7	1.8	2.1	2.2
LOC728218	0.0	0.1	0.1	2.7	4.1
LOC728342	0.0	0.1	0.2	2.5	5.3
LOC728392	0.0	0.2	0.3	7.5	13.5
LOC728724	0.0	0.4	0.1	Inf	Inf
LOC728739	0.0	0.1	0.2	Inf	Inf
LOC729683	0.5	1.3	1.3	2.9	2.8
LOC730091	0.0	0.1	0.2	2.1	3.6
LONRF1	2.1	5.9	5.6	2.8	2.7
LOXL2	0.0	0.1	0.2	2.6	3.2
LOXL4	0.2	0.1	0.0	0.4	0.1
LOX	0.1	0.3	0.6	2.6	4.7
LPAL2	0.1	0.2	0.4	2.1	3.3
LPAR4	0.0	0.2	0.2	13.9	13.6
LPAR6	0.1	0.2	0.3	3.5	5.8
LPCAT1	0.1	0.9	0.5	15.2	8.3
LRIG1	0.9	2.1	2.2	2.3	2.4
LRMP	2.8	6.0	5.7	2.1	2.0
LRP12	3.3	8.5	8.4	2.6	2.5
LRP2BP	0.1	0.2	0.1	3.3	2.1
LRRC16A	0.8	1.9	1.6	2.3	2.0

LRRC1	0.1	0.2	0.2	3.0	3.6
LRRC26	0.3	0.1	0.1	0.2	0.2
LRRC70	1.2	0.4	0.4	0.3	0.3
LRRC71	0.0	0.1	0.1	3.8	2.6
LRRC73	0.0	0.1	0.1	Inf	Inf
LRRC9	0.0	0.1	0.1	Inf	Inf
LRRK1	0.3	0.8	1.0	2.9	3.8
LRRN2	0.5	0.1	0.1	0.2	0.2
LSP1	0.3	1.1	0.7	3.3	2.1
LTB	0.0	0.3	0.1	Inf	Inf
LTK	0.1	0.2	0.2	2.9	2.3
LUM	0.0	0.3	0.2	Inf	Inf
LURAP1	0.0	0.1	0.1	8.3	5.2
LY6E	37.3	96.6	102.4	2.6	2.7
LY6G5C	0.4	1.5	1.0	4.1	2.7
LY6G6C	0.1	0.2	0.2	2.3	3.1
LY6G6D	1.3	3.1	3.0	2.4	2.3
LY75	0.2	2.0	2.2	9.4	10.2
LY9	0.4	0.0	0.1	#NAME?	0.3
LYNX1	0.1	0.4	0.3	3.8	3.2
LYPD6B	0.1	0.5	0.4	3.8	2.9
LYZ	1.8	11.0	8.9	6.2	5.0
MAB21L3	0.4	1.5	1.4	3.8	3.4
MAEA	33.8	16.0	15.1	0.5	0.4
MAF	0.0	0.4	0.2	9.6	6.1
MAGED1	15.9	36.2	37.0	2.3	2.3
MAGI2-AS3	0.1	0.6	0.9	6.4	9.5
MALAT1	57.5	118.4	144.2	2.1	2.5
MALL	0.0	0.1	0.2	4.9	6.0
MAL	0.3	1.1	1.2	3.3	3.6
MAML2	0.1	0.2	0.2	2.1	2.5
MAP10	0.5	1.1	1.1	2.1	2.1
MAP1A	1.1	2.9	3.8	2.6	3.5
MAP2	0.0	0.4	0.2	9.2	5.7
MAP3K12	1.2	2.7	2.6	2.3	2.1
MAP4K1	1.3	5.1	5.6	4.0	4.3
MAP7D2	0.3	0.9	1.1	2.6	3.2
MAP7	2.1	6.6	6.0	3.1	2.8
MAP9	0.4	1.7	1.7	4.1	4.1
MAPK11	0.3	0.7	0.6	2.2	2.0
MAPK12	1.5	7.7	8.5	5.2	5.8
MAPK15	0.0	0.1	0.2	7.6	10.4
1-Mar	0.1	0.6	0.4	6.6	4.3
2-Mar	17.5	6.8	6.7	0.4	0.4
MARK2P9	0.2	0.0	0.0	0.1	0.2
MARVELD1	4.0	11.0	11.7	2.8	2.9
MATK	0.5	1.6	1.5	3.3	3.0

MATN1	0.0	0.0	0.2	2.1	7.4
MATN2	0.1	0.5	0.6	3.4	4.3
MB21D2	0.0	0.3	0.1	6.0	3.2
MBNL1-AS1	0.1	0.2	0.2	4.6	3.8
MBOAT4	0.0	0.1	0.1	6.9	5.2
MCAM	0.0	0.2	0.2	7.4	6.3
MCTP1	1.0	3.7	3.3	3.7	3.2
MDFIC	0.1	0.3	0.2	4.3	2.6
MDGA1	0.0	0.2	0.2	29.8	29.1
MED12L	1.1	3.3	3.4	2.9	3.0
MEF2B	1.2	0.4	0.1	0.3	0.1
MEF2C	3.7	10.4	10.7	2.9	2.9
MEG3	0.2	0.8	1.2	3.6	5.1
MEGF6	0.1	0.4	0.4	4.6	4.3
MEI1	0.0	0.3	0.3	9.4	10.0
MEIS1	0.7	2.2	1.6	3.1	2.3
MEIS3	0.0	0.1	0.1	2.8	3.8
MEIS3P1	0.1	0.4	0.5	3.2	3.2
MERTK	0.2	0.1	0.1	0.4	0.4
MEST	3.8	9.4	8.8	2.4	2.3
METTL7B	0.1	0.3	0.6	2.9	6.5
MEX3A	0.2	0.8	1.0	4.6	5.7
MEX3B	3.0	6.8	6.7	2.3	2.2
MFHAS1	28.1	11.8	12.5	0.4	0.4
MFSD2A	0.6	1.6	2.6	2.5	4.0
MFSD2B	23.7	8.6	11.5	0.4	0.5
MFSD7	0.2	0.4	0.5	2.5	3.2
MGC16121	0.0	1.1	1.3	23.1	27.3
MIAT	1.1	3.8	3.7	3.5	3.5
MICAL1	1.7	3.8	3.9	2.3	2.3
MID2	0.0	0.2	0.1	5.1	3.1
MIR1248	78.5	0.0	0.0	#NAME?	#NAME?
MIR1260B	337.3	0.0	0.0	#NAME?	#NAME?
MIR155HG	0.3	0.7	0.9	2.7	3.4
MIR17HG	1.0	3.9	3.3	4.0	3.4
MIR223	219.5	0.0	22.6	#NAME?	0.1
MIR4269	494.4	0.0	0.0	#NAME?	#NAME?
MIR4440	160.4	0.0	0.0	#NAME?	#NAME?
MIR4513	424.3	0.0	0.0	#NAME?	#NAME?
MIR4690	5686.0	0.0	0.0	#NAME?	#NAME?
MIR4722	0.0	4364.6	6501.2	Inf	Inf
MIR4737	5597.1	739.5	2035.1	0.1	0.4
MIR499A	20.1	0.0	0.0	#NAME?	#NAME?
MIR5001	134.9	0.0	0.0	#NAME?	#NAME?
MIR5047	674.5	54.9	63.0	0.1	0.1
MIR548I2	3.9	0.0	0.0	#NAME?	#NAME?
MIR612	0.0	137.2	63.0	Inf	Inf

MIR659	174.7	0.0	0.0	#NAME?	#NAME?
MIR663A	6368.0	1797.5	2194.4	0.3	0.3
MIR663B	212.7	80.0	72.1	0.4	0.3
MIR941-1	29.9	0.0	0.0	#NAME?	#NAME?
MLLT10P1	0.2	0.4	1.0	2.5	6.4
MLLT11	1.4	6.8	6.6	5.0	4.9
MLPH	0.5	0.1	0.1	0.2	0.2
MLXIPL	0.0	0.1	0.1	3.9	2.8
MMP11	0.0	0.1	0.3	3.2	8.0
MMP17	0.1	0.3	0.4	2.1	3.1
MMP2	0.3	0.9	1.0	3.4	3.6
MMP9	0.0	0.3	0.3	10.7	9.9
MMRN1	2.1	7.2	6.5	3.4	3.1
MNDA	0.1	0.6	0.5	5.8	5.2
MORF4L2-AS	0.2	0.5	0.4	2.9	2.0
MPDZ	0.6	1.8	1.8	3.1	3.1
MPL	0.2	0.6	0.9	2.9	4.2
MPO	0.2	5.9	5.4	26.1	23.7
MPP7	1.6	3.4	3.5	2.1	2.2
MPZL3	0.7	0.2	0.3	0.3	0.4
MPZ	0.5	1.1	1.3	2.1	2.5
MRAP2	1.2	0.1	0.2	0.1	0.2
MRVI1	0.2	0.7	0.6	2.8	2.5
MS4A2	4.0	1.9	1.7	0.5	0.4
MS4A3	1.6	9.1	7.9	5.7	4.9
MS4A6A	0.2	0.0	0.1	#NAME?	0.4
MSC	0.1	0.4	0.4	2.6	2.8
MSMO1	23.5	70.1	64.4	3.0	2.7
MSRB3	1.7	3.6	3.4	2.1	2.0
MSTO2P	0.2	0.4	0.3	2.4	2.2
MSX2P1	0.1	0.5	0.5	5.2	5.6
MT1G	2.4	0.5	0.0	0.2	#NAME?
MTMR11	0.2	0.5	0.6	2.7	3.0
MTMR7	0.5	1.1	1.1	2.3	2.3
MTMR9LP	0.1	0.3	0.1	3.7	2.1
MTRNR2L2	411.3	907.6	866.1	2.2	2.1
MTRNR2L5	0.1	0.1	0.1	2.3	2.1
MTRNR2L6	0.3	0.9	0.8	3.0	2.6
MX2	0.0	0.4	0.3	20.5	16.3
MXI1	25.0	12.1	11.1	0.5	0.4
MYBL1	0.0	0.1	0.2	9.4	19.5
MYCT1	0.8	2.9	3.0	3.8	3.9
MYEF2	2.0	4.7	5.0	2.3	2.5
MYEOV	0.6	1.7	2.0	3.0	3.4
MYL10	0.1	0.0	0.0	0.2	#NAME?
MYLIP	6.5	2.9	1.6	0.4	0.2
MYLK4	0.1	0.7	0.5	4.9	3.5

MYLK	1.2	4.2	3.4	3.4	2.8
MYO10	0.1	0.3	0.3	3.5	3.6
MYO18B	0.1	0.3	0.2	2.9	2.0
MYO1F	1.6	4.3	4.2	2.7	2.6
MYO1G	2.1	15.0	17.7	7.3	8.6
MYO5A	0.3	0.7	0.8	2.3	2.8
MYOM1	0.2	1.0	1.2	4.6	5.6
MZB1	0.0	0.2	0.2	Inf	Inf
N4BP2	1.7	4.4	4.6	2.5	2.6
NAALAD2	0.0	0.3	0.2	6.8	4.7
NAALADL1	0.2	0.5	0.5	2.5	2.3
NAP1L2	0.0	0.3	0.3	12.5	11.0
NAP1L3	0.1	0.8	0.3	14.7	5.5
NAP1L6	0.0	0.1	0.0	6.9	2.1
NAT8L	0.0	0.1	0.1	17.5	27.5
NBEA	0.0	0.1	0.1	2.3	2.8
NBEAP1	0.2	0.9	1.0	4.0	4.3
NBL1	14.7	4.1	6.2	0.3	0.4
NBPF10	0.2	0.7	0.7	3.0	2.8
NBPF16	0.3	0.7	0.7	2.7	2.6
NCEH1	17.6	7.1	5.9	0.4	0.3
NCF1C	0.2	0.3	0.5	2.0	3.3
NCOR1P1	0.0	0.1	0.5	Inf	Inf
NDRG2	0.7	3.8	4.5	5.2	6.1
NDST3	0.0	0.1	0.1	2.9	2.9
NEDD8-MDP	0.0	2.7	0.0	522191.4	4.5
NEFH	2.0	4.5	4.1	2.2	2.0
NEK6	1.2	2.8	2.6	2.3	2.2
NES	0.0	0.1	0.2	9.8	15.3
NEU4	0.1	0.3	0.5	2.2	3.3
NEURL3	0.2	0.1	0.0	0.3	0.1
NEURL	0.3	0.7	0.7	2.5	2.7
NEXN	0.4	1.6	1.2	4.4	3.4
NFATC4	0.0	1.9	1.9	47.7	48.2
NFIB	0.1	0.5	0.4	7.3	5.4
NHSL2	0.0	0.1	0.1	2.1	4.6
NID1	0.2	2.0	1.7	11.9	10.5
NIPBL	6.0	12.7	13.2	2.1	2.2
NKAPL	0.0	0.1	0.1	6.2	5.2
NKG7	0.2	1.0	0.5	4.4	2.4
NKPD1	0.0	0.1	0.1	5.2	10.0
NLGN2	0.3	0.6	0.6	2.1	2.2
NLRC3	0.3	1.0	1.0	3.9	3.7
NLRC4	0.0	0.2	0.2	10.4	10.5
NLRP1	0.2	0.8	0.8	4.7	4.8
NLRP3	0.1	0.3	0.3	3.7	3.1
NME5	0.1	0.4	0.2	6.8	3.6

NMU	3.1	1.3	0.7	0.4	0.2
NOL3	0.5	1.0	1.2	2.2	2.6
NOMO3	0.2	0.4	0.4	2.5	2.4
NOS1AP	0.1	0.2	0.3	2.9	5.5
NOTCH1	0.4	1.0	1.0	2.6	2.6
NOTUM	0.1	0.3	0.4	2.1	2.8
NOV	0.1	0.5	0.3	5.0	3.4
NPC1L1	0.8	1.6	1.7	2.0	2.2
NPDC1	0.1	1.0	1.1	12.0	12.7
NPM2	0.5	1.6	1.8	3.4	3.8
NPR3	1.4	3.1	3.0	2.2	2.1
NPRL3	37.7	16.1	17.9	0.4	0.5
NPTX1	0.1	0.4	0.3	4.4	3.5
NQO1	4.8	18.3	17.3	3.8	3.6
NR0B1	0.0	0.0	0.1	Inf	Inf
NR5A2	0.2	1.1	0.9	4.7	3.8
NRARP	0.9	4.4	2.9	5.0	3.3
NRG4	0.2	0.7	0.7	4.3	4.3
NRP2	0.0	0.2	0.2	8.0	6.4
NRTN	0.2	0.0	0.0	0.3	#NAME?
NRXN2	0.1	0.9	0.8	15.1	13.6
NSRP1	7.0	15.3	14.7	2.2	2.1
NSUN5P2	1.0	2.7	2.7	2.6	2.6
NT5C1B-RDH	0.0	0.1	0.0	9069.2	1796.4
NTNG2	0.2	0.6	0.7	2.4	2.8
NTRK1	5.1	11.3	11.7	2.2	2.3
NTRK3	0.0	0.2	0.2	12.2	11.8
NTS	3.7	11.3	7.5	3.1	2.0
NUDT11	0.0	0.5	0.5	13.8	12.9
NUDT7	5.5	1.7	2.1	0.3	0.4
NXF3	0.1	0.3	0.4	4.3	5.7
NXPH4	0.0	0.1	0.1	2.1	3.6
NYAP1	0.9	0.3	0.3	0.4	0.3
NYNRIN	1.4	3.7	4.1	2.6	2.9
OAF	1.2	2.8	3.8	2.4	3.3
OAS1	0.0	0.4	0.1	Inf	Inf
OBSCN	0.2	0.8	0.9	3.6	4.1
OBSL1	0.7	3.5	3.8	5.1	5.6
ODF2L	2.5	5.4	5.0	2.2	2.0
OR10Z1	0.0	0.4	0.3	Inf	Inf
OR2AT4	0.0	0.2	0.3	4.7	7.2
OR2B6	0.0	0.2	0.2	Inf	Inf
OR52H1	0.0	0.1	0.1	Inf	Inf
OR56B1	0.0	0.0	0.1	Inf	Inf
OR7E91P	0.1	0.0	0.0	#NAME?	#NAME?
OR8G2	0.1	0.0	0.0	#NAME?	#NAME?
ORAI2	2.8	6.0	6.2	2.2	2.2

OSBPL6	4.1	10.2	10.1	2.5	2.5
OSGIN1	1.6	5.7	6.0	3.5	3.7
OVOL3	0.0	0.1	0.1	Inf	Inf
OXER1	0.0	0.1	0.1	6.2	5.2
P2RY12	0.1	0.7	0.4	7.3	4.7
P4HA2	0.1	0.4	0.3	2.8	2.2
PAK1	29.8	10.8	10.7	0.4	0.4
PALM	0.6	1.3	1.8	2.0	2.9
PAM	3.0	9.3	9.0	3.1	3.0
PAN3-AS1	0.4	1.3	1.5	3.0	3.5
PAOX	0.2	0.8	0.5	3.8	2.3
PAQR8	0.6	1.5	1.2	2.6	2.0
PAQR9	12.4	1.7	2.0	0.1	0.2
PAR5	0.3	1.1	1.2	3.4	3.5
PARD3	0.3	0.0	0.1	0.2	0.2
PARP8	0.4	1.3	1.2	3.7	3.5
PARVG	0.8	7.8	6.9	10.2	9.0
PAWR	1.0	3.5	2.9	3.6	3.0
PCBP4	0.5	1.3	1.3	2.6	2.6
PCDH1	0.3	0.1	0.2	0.4	0.4
PCDH20	0.0	0.2	0.2	16.1	14.2
PCDH9	0.1	0.2	0.2	3.4	3.5
PCDHGB3	0.0	0.0	0.1	2.1	10.5
PCDHGB5	0.0	0.1	0.1	2.4	5.6
PCK2	49.6	16.0	17.5	0.3	0.4
PCNXL2	0.0	0.1	0.2	Inf	Inf
PCSK1N	0.3	1.3	0.7	5.0	2.6
PCSK1	0.0	0.1	0.1	18.8	12.6
PDCD1	0.0	0.2	0.2	6.6	5.2
PDE4D	0.2	0.6	0.7	3.6	3.9
PDE5A	0.5	1.3	1.0	2.7	2.2
PDE6B	0.8	1.9	2.3	2.3	2.8
PDE6H	0.0	0.2	0.1	Inf	Inf
PDE8B	0.1	0.2	0.2	4.0	2.8
PDGFD	0.1	0.5	0.8	5.4	8.2
PDGFRA	0.0	0.1	0.1	12.6	8.5
PDK2	1.1	6.0	7.9	5.4	7.1
PDLIM2	0.1	0.4	0.3	4.1	2.8
PDLIM4	0.1	0.2	0.2	2.2	2.4
PDZK1IP1	2.4	0.2	0.7	0.1	0.3
PEAR1	5.2	10.5	11.1	2.0	2.1
PECAM1	0.9	2.6	2.6	3.0	3.0
PEG3-AS1	0.0	0.1	0.2	4.8	6.2
PEG3	0.0	0.1	0.1	4.1	2.1
PER1	44.8	15.7	14.3	0.4	0.3
PEX3	4.1	13.6	12.1	3.3	2.9
PF4	20.9	55.2	48.9	2.6	2.3

PFN2	0.0	0.2	0.1	8.6	6.1
PGAP1	0.0	0.2	0.1	3.8	2.4
PGF	3.5	1.2	1.2	0.3	0.3
PGM1	0.5	4.1	4.6	7.7	8.8
PHACTR1	0.5	1.2	1.1	2.3	2.2
PHGDH	1.5	0.4	0.4	0.2	0.3
PHIP	4.8	11.4	11.0	2.4	2.3
PHKA1	0.3	0.6	0.7	2.1	2.5
PHLDA1	0.4	0.9	1.1	2.4	3.1
PHLDB1	0.7	1.9	2.2	2.6	3.2
PHOSPHO1	20.3	3.6	4.8	0.2	0.2
PHOSPHO2-K	0.0	0.3	0.2	2108.7	1044.2
PIEZO2	0.0	0.1	0.2	11.2	20.5
PIFO	0.2	1.1	1.0	4.8	4.1
PIGQ	60.5	21.8	26.4	0.4	0.4
PIK3C2A	2.0	4.3	4.6	2.2	2.3
PIK3CD	0.6	2.2	2.8	3.8	4.8
PIK3R5	0.0	0.5	0.5	16.7	17.2
PIK3R6	0.6	2.1	1.9	3.4	3.0
PILRA	0.3	0.9	0.7	3.1	2.3
PIR-FIGF	0.0	0.6	0.0	5607241467649.7	364547930128.2
PITPNM2	1.0	3.5	3.7	3.6	3.7
PITX1	2.5	0.3	0.3	0.1	0.1
PIWIL3	0.2	0.9	0.7	5.0	3.9
PKD1L3	0.3	0.0	0.1	0.1	0.3
PKDCC	0.4	0.2	0.1	0.4	0.3
PKHD1L1	0.3	1.0	1.1	3.1	3.3
PLA2G1B	0.1	0.4	0.4	5.7	5.9
PLA2G4B	2.4	0.8	0.2	0.4	0.1
PLA2G7	0.0	0.2	0.2	8.2	11.5
PLAC8	1.4	5.3	5.0	3.7	3.5
PLCB2	2.4	7.1	7.4	2.9	3.1
PLCB4	0.1	0.6	0.4	5.0	3.5
PLCH1	0.1	0.3	0.3	4.1	3.6
PLEKHA2	3.7	8.2	8.3	2.2	2.3
PLEKHB1	0.1	0.3	0.3	3.9	4.2
PLEKHG1	0.4	1.2	1.3	3.1	3.3
PLEKHG2	0.5	1.6	1.6	3.0	3.0
PLEKHG3	0.2	1.5	1.1	9.0	6.2
PLEKHG5	0.0	0.1	0.1	3.4	3.5
PLEKHH2	0.2	0.1	0.1	0.4	0.4
PLEKHH3	39.3	5.3	5.4	0.1	0.1
PLEK	19.4	58.0	51.5	3.0	2.7
PLIN5	0.9	0.3	0.4	0.4	0.4
PLOD2	1.3	3.7	3.7	2.8	2.8
PLS3	0.1	1.7	1.2	18.2	12.7
PLSCR3	1.8	5.5	5.0	3.0	2.7

PLXDC2	1.3	2.9	2.6	2.3	2.1
PLXNB1	0.0	0.3	0.2	9.9	7.4
PMM1	17.0	5.1	5.4	0.3	0.3
PNLIP	0.1	0.4	0.5	6.2	7.3
PNMAL1	0.3	0.7	0.8	2.8	3.2
PNPLA3	0.1	0.4	0.5	3.7	4.4
POLK	3.2	7.2	6.8	2.2	2.1
POU4F1	0.4	0.8	0.8	2.2	2.1
PPAPDC1A	4.2	0.8	0.4	0.2	0.1
PPBP	53.5	122.9	117.0	2.3	2.2
PPFIBP1	1.6	5.7	5.6	3.7	3.6
PPIF	48.3	110.7	100.7	2.3	2.1
PPIL6	0.1	0.1	0.2	2.8	3.5
PPM1N	0.1	0.6	0.7	6.2	7.1
PPP1R14A	0.8	5.6	5.2	7.3	6.8
PPP1R15A	94.5	41.4	37.1	0.4	0.4
PPP1R16B	0.2	2.3	2.3	12.3	12.3
PPP1R18	3.4	10.0	9.7	2.9	2.8
PPP2R5B	16.5	4.4	4.2	0.3	0.3
PPP4R1L	0.5	0.2	0.2	0.4	0.4
PRAM1	0.0	0.1	0.2	3.8	5.8
PRDX2	861.2	356.9	379.0	0.4	0.4
PRF1	0.0	0.1	0.1	Inf	Inf
PRH2	0.0	0.1	0.1	2.2	3.6
PRKACB	7.0	32.5	26.6	4.7	3.8
PRKAG2	2.2	7.6	6.6	3.4	3.0
PRKAR2A	11.7	5.6	5.6	0.5	0.5
PRKD1	0.1	0.5	0.3	3.6	2.3
PRKG1-AS1	0.0	0.2	0.1	3.7	3.5
PRKG1	0.2	0.5	0.6	3.3	3.9
PROB1	0.2	0.5	0.4	3.3	2.7
PROCR	0.2	0.5	0.4	3.3	2.7
PRODH	0.1	0.3	0.3	3.1	2.8
PROM1	0.1	0.2	0.3	2.1	3.3
PROX2	0.1	0.2	0.3	3.5	5.8
PRR4	0.0	0.4	0.0	20300.3	4.8
PRR5-ARHGA	0.0	0.2	0.0	19351826.9	66.6
PRR5L	0.1	0.2	0.3	3.0	3.7
PRR7	1.2	2.4	2.9	2.0	2.4
PRRG1	0.2	0.6	0.7	3.5	4.1
PRRT2	0.1	0.3	0.4	3.8	5.1
PRSS23	0.7	3.5	3.2	5.1	4.8
PRSS36	0.1	0.2	0.2	2.3	2.6
PRSS3	0.0	0.9	0.1	Inf	Inf
PRSS57	7.2	19.1	21.4	2.6	3.0
PRSS8	0.4	0.1	0.2	0.4	0.4
PRTFDC1	1.4	4.6	3.4	3.3	2.4

PRTN3	0.0	0.4	0.1	12.1	4.1
PRUNE2	0.1	0.6	0.5	5.1	4.7
PSAT1	23.9	8.2	6.4	0.3	0.3
PSMB8	10.7	26.7	25.0	2.5	2.3
PSMB9	1.3	4.8	4.3	3.8	3.4
PSPN	0.0	0.2	0.3	Inf	Inf
PTCRA	0.7	0.1	0.0	0.2	#NAME?
PTGDR2	0.3	0.8	0.8	2.7	2.8
PTGDS	0.8	3.0	3.4	3.7	4.2
PTGER2	0.6	3.8	3.4	6.0	5.4
PTGER3	0.1	1.4	1.2	10.6	9.7
PTGER4P2-CI	0.0	0.1	0.1	4.4	3.2
PTGES	0.4	0.0	0.0	0.1	0.1
PTH2R	0.1	0.3	0.3	4.8	3.9
PTHLH	0.1	0.0	0.0	0.1	#NAME?
PTK7	0.3	1.4	1.4	4.8	5.0
PTP4A3	0.3	3.0	3.2	11.1	11.7
PTPDC1	0.8	0.3	0.3	0.3	0.4
PTPLAD2	7.2	15.0	15.3	2.1	2.1
PTPN13	0.1	0.6	0.5	6.7	5.7
PTPN22	3.4	9.0	8.4	2.7	2.5
PTPRCAP	1.1	2.3	2.5	2.1	2.3
PTPRC	8.6	25.9	23.3	3.0	2.7
PTPRH	0.4	0.1	0.1	0.2	0.4
PTPRR	0.4	0.9	1.1	2.1	2.6
PTPRS	0.1	0.4	0.4	6.0	6.3
PTX3	0.0	0.3	0.5	Inf	Inf
PUS7L	2.7	5.5	6.5	2.0	2.4
PVALB	1.7	4.9	4.8	2.9	2.9
PVRIG	0.4	0.8	0.9	2.2	2.3
PYCRL	5.7	2.7	2.8	0.5	0.5
QPCT	0.1	0.6	0.3	7.4	3.9
RAB11FIP4	0.2	1.0	1.2	6.2	7.5
RAB11FIP5	0.0	0.5	0.7	12.1	16.3
RAB20	0.1	0.4	0.2	5.7	2.4
RAB27B	3.3	16.8	14.2	5.1	4.3
RAB31	3.1	8.3	8.8	2.7	2.9
RAB32	2.3	8.7	6.4	3.8	2.7
RAB33A	1.4	3.6	3.9	2.7	2.9
RAB34	0.9	3.7	2.3	4.3	2.7
RAB36	0.1	0.6	0.5	4.2	3.4
RAB37	3.5	9.8	9.7	2.8	2.8
RAB3A	0.4	0.2	0.1	0.4	0.2
RAB3D	0.5	1.5	1.4	2.9	2.8
RAB3IL1	27.5	11.7	13.6	0.4	0.5
RAB44	0.3	1.1	1.1	3.1	3.1
RAB4B-EGLN	0.0	0.6	0.6	43883.0	42676.4

RAB9B	0.3	0.9	0.9	3.5	3.7
RAC3	2.7	8.7	8.9	3.2	3.3
RAD23A	127.6	59.5	62.3	0.5	0.5
RAD51L3-RFI	0.7	0.0	0.0	0.0	0.0
RAET1E	0.4	1.6	1.1	4.1	2.8
RAET1G	0.1	0.3	0.5	5.4	8.7
RALGPS1	0.1	0.2	0.2	3.0	2.8
RAMP1	2.5	8.0	7.6	3.2	3.0
RANBP2	5.6	14.4	14.1	2.6	2.5
RAP1GAP2	0.3	0.9	0.9	3.2	3.1
RAPGEF3	0.0	0.1	0.1	3.1	2.2
RARB	0.0	0.1	0.1	11.1	8.4
RASA4	0.9	2.8	2.7	3.1	2.9
RASAL2-AS1	0.1	0.0	0.1	0.3	0.4
RASGRP1	0.1	0.2	0.2	2.7	2.8
RASGRP2	1.8	3.7	4.2	2.1	2.3
RASIP1	1.0	0.4	0.3	0.4	0.3
RASSF2	0.3	1.3	1.1	3.7	3.2
RB1CC1	6.1	13.0	13.2	2.1	2.2
RBAK-LOC389	0.0	0.3	0.0	170262860.8	109941.6
RBKS	2.0	0.8	0.6	0.4	0.3
RBM11	0.1	0.3	0.3	4.7	4.4
RBM38	76.5	33.6	37.6	0.4	0.5
RBM41	7.2	15.6	14.4	2.2	2.0
RBPMS2	0.3	2.1	1.7	6.1	4.8
RCAN3AS	0.2	0.8	1.1	4.5	6.3
RCAN3	0.5	1.5	1.4	2.9	2.7
RCOR2	0.0	0.2	0.1	15.9	9.4
RDH10	4.5	11.9	11.0	2.6	2.4
RDH12	0.0	0.1	0.1	Inf	Inf
RELL2	0.7	1.9	2.2	2.9	3.2
RELN	0.1	0.0	0.0	0.4	0.4
REV3L	3.1	8.7	8.4	2.8	2.7
RFTN1	5.2	12.9	11.5	2.5	2.2
RFX8	0.0	0.6	0.5	16.1	12.5
RGAG1	0.1	0.2	0.4	2.4	4.6
RGAG4	0.0	0.2	0.2	5.0	3.5
RGPD4	0.1	0.2	0.1	3.0	2.2
RGS18	2.2	9.2	8.3	4.2	3.7
RGS1	0.1	0.2	0.3	2.0	3.1
RGS6	2.0	5.7	6.5	2.9	3.3
RGS9	0.3	0.0	0.1	0.0	0.4
RHD	11.4	4.5	5.3	0.4	0.5
RHEBL1	2.9	7.1	6.5	2.5	2.2
RHOBTB1	0.8	2.0	2.6	2.6	3.4
RHOC	14.4	41.7	43.5	2.9	3.0
RHOQ	2.6	6.6	5.3	2.6	2.1

RHOU	0.1	0.5	0.5	6.0	6.3
RHOV	1.0	0.4	0.4	0.4	0.4
RICTOR	3.5	7.3	7.5	2.1	2.2
RIF1	9.0	21.5	21.9	2.4	2.4
RIIAD1	0.2	0.0	0.0	#NAME?	#NAME?
RIN1	0.1	0.2	0.2	2.4	2.4
RIPK3	0.3	1.6	2.0	5.5	6.9
RLN3	0.4	11.5	11.0	27.5	26.3
RLTPR	0.5	1.1	1.0	2.2	2.1
RNASE2	7.6	43.4	39.6	5.7	5.2
RNASE3	0.3	2.0	1.7	6.3	5.4
RNASE6	0.5	1.6	2.0	3.2	4.2
RNF151	0.0	0.0	0.1	Inf	Inf
RNF217	1.4	3.8	4.0	2.7	2.8
RNF5	0.4	0.0	0.0	0.0	0.0
RNU11	0.0	13.0	22.2	Inf	Inf
RNU6ATAC	80.3	0.0	0.0	#NAME?	#NAME?
ROBO1	0.1	0.3	0.4	3.7	5.1
ROBO3	0.2	0.7	0.7	4.1	4.2
ROCK1	5.9	13.4	13.1	2.3	2.2
ROR1	0.0	0.2	0.1	33.3	20.1
ROR2	0.1	0.0	0.0	#NAME?	#NAME?
RPS4Y2	0.2	0.1	0.0	0.3	0.3
RPS6KA2	0.1	0.0	0.0	0.3	0.2
RPS6KL1	9.9	3.4	3.5	0.3	0.3
RRAGD	2.7	5.7	5.6	2.1	2.1
RRM2	110.5	44.1	39.3	0.4	0.4
RRN3P1	0.9	2.3	2.6	2.5	2.8
RSPH1	0.1	0.4	0.4	3.4	3.5
RTBDN	0.0	0.1	0.1	4.2	2.6
RTKN2	0.1	0.4	0.3	5.3	3.8
RTKN	0.1	0.2	0.4	2.4	4.0
RTN1	0.8	5.7	4.8	7.2	6.0
RTP4	0.0	0.6	1.2	16.1	33.8
RUNDC3A	1.0	0.4	0.4	0.4	0.4
RUNX3	1.5	3.7	3.4	2.5	2.3
RXFP1	0.0	0.0	0.2	Inf	Inf
RXFP4	0.2	0.1	0.1	0.3	0.4
RYR2	0.0	0.1	0.1	11.4	5.5
S100A7	0.2	0.0	0.0	#NAME?	#NAME?
S100A8	0.1	0.6	0.2	6.9	2.9
S100A9	0.0	0.8	0.1	Inf	Inf
SACS	2.1	6.3	6.7	3.0	3.2
SALL4	0.9	0.4	0.4	0.4	0.4
SAMD12-AS1	0.1	0.2	0.3	3.3	6.0
SAMD14	0.2	0.4	0.4	2.1	2.3
SAMD15	0.1	0.2	0.4	2.6	5.0

SAMD5	0.2	0.0	0.0	0.2	0.1
SAMD9L	1.3	3.1	3.8	2.5	3.0
SAMD9	2.3	6.8	6.7	3.0	2.9
SARDH	0.0	0.2	0.3	22.3	33.6
SARM1	0.2	0.5	0.4	2.1	2.1
SASH3	0.3	2.2	1.6	8.2	5.9
SBF2	0.4	0.1	0.2	0.4	0.5
SCAF11	9.2	20.2	20.5	2.2	2.2
SCAMP5	0.1	0.3	0.3	3.2	3.3
SCARNA7	0.9	0.2	0.3	0.2	0.4
SCARNA8	0.0	6.9	4.6	Inf	Inf
SCARNA9L	0.2	0.6	0.6	3.4	3.6
SCD5	0.2	0.7	0.5	3.5	2.3
SCG2	0.2	0.6	0.5	3.2	3.0
SCGB1B2P	0.0	0.1	0.1	Inf	Inf
SCGB2B2	0.1	0.0	0.0	#NAME?	#NAME?
SCHIP1	0.3	1.6	1.5	5.1	4.8
SCIMP	0.0	0.1	0.1	2.1	3.5
SCN1B	0.2	0.9	1.5	4.0	6.4
SCN4A	0.2	0.6	0.8	3.1	4.1
SCN9A	0.1	0.8	0.7	6.2	5.2
SCRN1	3.2	8.2	9.3	2.6	2.9
SCT	0.2	0.0	0.0	#NAME?	#NAME?
SCUBE1	0.0	0.2	0.2	9.8	10.0
SDR42E1	0.0	0.0	0.1	2.8	10.5
SEC14L3	1.1	0.0	0.1	0.0	0.1
SEC14L4	13.7	3.1	3.3	0.2	0.2
SECTM1	0.1	0.7	0.9	6.1	8.1
SELENBP1	62.8	6.8	10.0	0.1	0.2
SELL	0.3	0.7	0.8	2.6	2.9
SELM	0.6	1.6	1.3	2.8	2.3
SEMA3B	0.0	0.1	0.1	8.3	10.5
SEMA3C	0.1	0.6	0.4	7.4	5.2
SEMA3F	0.0	0.1	0.1	4.2	7.4
SEMA3G	0.1	0.3	0.3	2.1	2.2
SEMA4A	0.2	3.6	3.7	16.9	17.3
10-Sep	1.0	3.6	3.6	3.5	3.5
SEPT5-GP1BE	3.1	9.4	8.2	3.0	2.6
5-Sep	3.6	11.7	14.6	3.3	4.1
6-Sep	4.4	16.0	15.1	3.7	3.5
SERF2-C15OF	0.2	0.0	0.0	0.0	0.0
SERHL2	0.3	0.7	1.0	2.3	3.4
SERPINB10	0.0	0.1	0.1	Inf	Inf
SERPINB2	0.0	0.2	0.1	Inf	Inf
SERPINB8	0.2	0.8	1.0	4.7	5.9
SERPINE2	12.2	53.7	51.9	4.4	4.3
SERPING1	1.8	9.0	9.0	5.0	5.0

SERPINI1	2.0	9.1	8.9	4.5	4.4
SERTAD3	9.4	4.2	4.3	0.5	0.5
SESN2	10.2	4.0	3.4	0.4	0.3
SETD7	0.6	2.1	1.9	3.7	3.4
SFRP4	0.0	0.1	0.1	6.9	12.6
SGIP1	0.0	0.1	0.1	4.2	4.0
SGMS2	0.2	0.3	0.5	2.2	3.2
SGTB	0.7	1.7	1.4	2.6	2.1
SH2D1A	0.1	0.3	0.3	3.0	4.0
SH2D2A	4.1	13.1	12.7	3.2	3.1
SH3BP4	0.0	0.1	0.2	4.5	10.0
SH3GL1P2	0.0	0.1	0.1	2.8	3.8
SH3RF2	0.3	0.8	0.9	2.4	2.8
SHANK1	0.9	2.1	2.8	2.3	3.0
SHANK2	0.0	0.1	0.1	3.3	5.1
SHANK3	0.0	0.6	0.5	13.7	12.8
SHC4	0.5	1.0	1.0	2.1	2.1
SHF	0.0	0.2	0.2	3.6	4.7
SHISA4	1.4	2.8	3.4	2.0	2.4
SHISA9	1.0	2.7	2.5	2.8	2.6
SIAH3	0.0	0.1	0.2	4.5	11.6
SIGLEC9	0.0	0.1	0.1	Inf	Inf
SIPA1L2	0.1	0.1	0.0	0.5	0.3
SIRPA	0.2	1.2	1.2	6.4	6.3
SIRT4	1.0	0.5	0.2	0.5	0.2
SKIL	0.8	1.7	1.8	2.1	2.2
SKOR1	0.0	0.1	0.1	5.2	3.7
SLAIN1	2.7	5.8	5.7	2.1	2.1
SLAMF7	0.1	0.2	0.3	2.2	2.6
SLA	1.0	7.3	8.1	7.0	7.8
SLC12A2	0.7	2.2	1.6	3.1	2.2
SLC12A8	0.1	0.1	0.1	2.7	2.5
SLC16A14	0.0	0.2	0.1	11.9	4.2
SLC16A3	5.7	12.8	14.5	2.2	2.5
SLC16A5	0.6	1.7	1.7	2.8	2.7
SLC17A7	0.0	0.1	0.2	6.6	8.9
SLC18A2	4.2	11.2	9.7	2.7	2.3
SLC1A5	113.8	54.6	53.0	0.5	0.5
SLC22A15	0.2	0.9	0.7	4.8	3.9
SLC22A16	8.1	4.0	3.5	0.5	0.4
SLC22A17	0.9	6.7	8.5	7.7	9.9
SLC24A3	2.5	5.3	5.1	2.1	2.0
SLC24A5	0.0	0.2	0.3	Inf	Inf
SLC25A24	0.3	1.3	1.0	3.9	3.0
SLC25A27	0.2	1.3	1.1	6.4	5.2
SLC25A29	3.6	8.3	9.8	2.3	2.8
SLC25A37	272.5	69.8	81.4	0.3	0.3

SLC25A39	249.8	88.4	101.2	0.4	0.4
SLC25A41	0.0	0.1	0.1	Inf	Inf
SLC25A52	0.1	0.2	0.1	3.0	2.6
SLC25A53	0.3	1.2	1.4	4.5	5.2
SLC2A6	0.5	1.3	1.4	2.9	3.0
SLC2A9	0.1	0.4	0.3	3.1	2.5
SLC30A10	0.5	0.1	0.1	0.2	0.1
SLC35F2	1.2	3.2	3.5	2.7	2.9
SLC35G2	0.1	0.3	0.4	2.1	2.7
SLC38A1	4.8	13.0	12.3	2.7	2.6
SLC39A14	1.2	4.2	3.6	3.4	2.9
SLC3A2	164.1	72.3	71.8	0.4	0.4
SLC43A1	47.4	17.7	21.4	0.4	0.5
SLC46A1	1.1	2.6	2.4	2.4	2.3
SLC46A2	0.0	0.1	0.2	Inf	Inf
SLC4A1	313.7	29.2	39.3	0.1	0.1
SLC4A4	0.0	0.1	0.1	17.2	19.0
SLC4A7	0.8	2.5	2.5	3.1	3.1
SLC6A13	0.0	0.2	0.3	Inf	Inf
SLC6A19	1.4	0.6	0.5	0.4	0.4
SLC6A9	23.0	2.1	2.7	0.1	0.1
SLC7A5	62.6	19.2	21.5	0.3	0.3
SLC7A7	0.0	0.2	0.4	4.8	9.9
SLC7A8	0.2	0.7	0.7	3.5	3.8
SLC9A3R2	1.4	4.1	4.8	2.8	3.3
SLC9A9	0.6	1.9	1.9	3.2	3.1
SLCO2B1	7.1	14.9	16.0	2.1	2.3
SLCO3A1	0.8	2.2	2.2	2.7	2.7
SLCO4C1	0.6	1.9	1.5	3.2	2.5
SLCO5A1	0.2	0.1	0.0	0.3	0.1
SLFN12L	0.0	0.2	0.1	11.7	6.3
SLFN12	1.0	2.3	2.1	2.2	2.0
SLITRK6	0.0	0.2	0.1	4.3	2.7
SMAD6	0.1	1.5	1.5	18.0	17.1
SMAD9	0.6	0.2	0.3	0.3	0.5
SMARCD3	0.2	1.2	0.8	5.5	3.7
SMIM1	77.4	32.8	38.2	0.4	0.5
SMIM3	2.4	5.9	6.8	2.4	2.8
SMIM5	26.4	10.2	12.8	0.4	0.5
SMIM6	0.3	1.1	1.7	4.3	6.4
SMKR1	0.5	2.8	2.5	5.5	4.9
SMO	0.0	0.2	0.1	5.9	2.7
SMPDL3A	0.0	0.0	0.2	2.8	11.5
SMTNL1	0.3	0.1	0.1	0.3	0.4
SNCG	0.0	0.4	0.5	8.0	10.1
SND1-IT1	0.0	0.0	0.1	2.8	8.4
SNN	2.0	0.9	0.9	0.5	0.4

SNORA10	37.4	10.3	12.4	0.3	0.3
SNORA16A	8.8	0.0	3.9	#NAME?	0.4
SNORA18	39.8	15.2	17.5	0.4	0.4
SNORA19	14.9	0.0	0.0	#NAME?	#NAME?
SNORA20	0.0	13.0	4.4	Inf	Inf
SNORA24	10.6	0.0	4.6	#NAME?	0.4
SNORA2A	0.0	18.6	3.7	Inf	Inf
SNORA2B	7.3	0.0	0.0	#NAME?	#NAME?
SNORA34	0.0	1.7	6.7	Inf	Inf
SNORA3	11.3	0.0	4.9	#NAME?	0.4
SNORA43	0.0	0.0	8.3	Inf	Inf
SNORA45	10.6	2.3	0.0	0.2	#NAME?
SNORA4	22.0	10.1	6.7	0.5	0.3
SNORA51	10.0	4.3	4.4	0.4	0.4
SNORA55	7.3	0.0	0.0	#NAME?	#NAME?
SNORA58	0.4	0.2	0.0	0.5	#NAME?
SNORA5C	29.3	5.1	3.3	0.2	0.1
SNORA60	7.8	3.5	0.0	0.5	#NAME?
SNORA62	6.3	0.9	0.0	0.1	#NAME?
SNORA65	23.3	0.0	0.0	#NAME?	#NAME?
SNORA66	9.3	0.0	0.0	#NAME?	#NAME?
SNORA69	10.0	0.0	4.4	#NAME?	0.4
SNORA74B	0.0	0.3	0.5	Inf	Inf
SNORA75	14.7	0.0	3.3	#NAME?	0.2
SNORA77	64.3	22.8	13.5	0.4	0.2
SNORA79	6.2	0.0	0.0	#NAME?	#NAME?
SNORD100	1838.4	441.2	0.0	0.2	#NAME?
SNORD110	0.0	497.0	938.0	Inf	Inf
SNORD116-4	320.8	68.0	156.4	0.2	0.5
SNORD119	190.2	0.0	0.0	#NAME?	#NAME?
SNORD12B	103.1	0.0	0.0	#NAME?	#NAME?
SNORD16	134.9	0.0	0.0	#NAME?	#NAME?
SNORD23	164.6	0.0	22.6	#NAME?	0.1
SNORD26	998.4	497.0	0.0	0.5	#NAME?
SNORD2	4706.3	0.0	0.0	#NAME?	#NAME?
SNORD33	0.0	581.4	821.7	Inf	Inf
SNORD36A	3894.7	1440.6	0.0	0.4	#NAME?
SNORD46	160.4	0.0	0.0	#NAME?	#NAME?
SNORD79	621.9	0.0	0.0	#NAME?	#NAME?
SNORD80	5699.4	2453.0	1441.1	0.4	0.3
SNORD83A	207.1	0.0	0.0	#NAME?	#NAME?
SNORD83B	244.9	59.9	0.0	0.2	#NAME?
SNORD88B	174.7	37.9	0.0	0.2	#NAME?
SNPH	0.1	0.6	0.6	4.2	4.5
SNX10	2.1	5.3	4.6	2.6	2.2
SNX16	1.5	3.2	3.1	2.1	2.0
SNX20	2.3	6.1	5.8	2.6	2.5

SNX22	14.7	6.1	6.8	0.4	0.5
SNX32	0.2	0.5	0.8	2.2	4.1
SOHLH2	0.0	0.3	0.0	3572676.7	2.7
SORBS3	0.1	0.2	0.2	3.4	3.9
SORL1	0.1	0.4	0.4	5.3	5.3
SOS1	6.7	19.1	18.2	2.8	2.7
SOWAHC	0.6	3.8	3.9	6.2	6.3
SOX6	2.4	0.4	0.6	0.2	0.3
SPARC	1.6	6.4	6.2	4.0	3.9
SPATA25	0.5	0.2	0.2	0.4	0.4
SPATA6	0.0	0.1	0.1	4.7	2.4
SPATA9	0.2	0.8	0.6	3.8	2.7
SPDYC	0.1	0.7	0.9	5.8	7.1
SPDYE1	0.2	0.4	0.5	2.4	3.1
SPEG	0.0	0.1	0.1	19.9	37.8
SPIN3	1.7	3.4	3.3	2.1	2.0
SPINK1	0.0	0.5	0.2	Inf	Inf
SPINK2	0.0	0.4	0.2	Inf	Inf
SPINT1	0.4	1.7	2.2	4.5	5.7
SPIRE1	0.3	1.3	1.2	4.7	4.3
SPOCK1	0.2	0.7	0.7	3.5	3.5
SPOCK3	0.1	0.0	0.0	0.1	#NAME?
SPP1	1.6	7.6	8.1	4.8	5.1
SPRY4	0.4	0.9	1.2	2.3	2.8
SPTBN1	0.3	1.2	1.1	3.9	3.6
SPTB	136.2	45.9	53.9	0.3	0.4
SPTLC3	0.1	0.5	0.5	6.0	5.8
SPTY2D1-AS1	0.1	0.2	0.4	3.4	6.6
SRC	1.8	3.9	3.8	2.2	2.1
SREBF1	92.0	33.4	39.9	0.4	0.4
SRGN	90.4	300.2	258.8	3.3	2.9
SRMS	0.0	0.0	0.1	Inf	Inf
SRPX2	0.1	0.4	0.3	3.7	3.1
ST20-MTHFS	0.0	0.1	0.1	676732.3	640736.1
ST3GAL4	1.7	5.6	7.0	3.4	4.2
ST3GAL5	0.1	0.4	0.5	3.9	4.6
ST3GAL6	0.2	0.9	0.6	4.5	3.0
ST6GAL2	0.1	0.2	0.3	3.4	5.3
ST6GALNAC4	40.7	16.0	19.3	0.4	0.5
ST8SIA6	8.6	19.4	19.0	2.3	2.2
STAB1	0.1	0.6	0.5	6.6	5.7
STAC3	1.5	4.6	4.3	3.0	2.8
STAC	1.5	3.6	4.0	2.4	2.6
STAMBPL1	0.5	1.7	1.6	3.2	3.0
STAP1	2.8	9.1	9.7	3.3	3.5
STARD9	0.1	0.3	0.3	2.5	2.4
STAR	0.8	2.2	1.7	2.7	2.1

STAT4	0.1	0.4	0.5	4.3	5.4
STAU2-AS1	0.1	0.2	0.2	2.1	2.1
STEAP4	0.0	0.0	0.1	Inf	Inf
STK33	0.2	0.6	0.6	2.9	3.0
STK39	3.2	8.7	8.5	2.8	2.7
STL	0.1	0.1	0.1	2.2	2.3
STON1	0.3	0.8	1.0	2.5	3.3
STRADB	36.6	17.3	15.5	0.5	0.4
STX16-NPEPL	0.3	1.3	1.1	4.3	3.9
STX1B	0.5	1.1	1.1	2.1	2.2
STXBP1	2.8	5.8	7.0	2.1	2.5
STXBP5	4.3	11.0	11.0	2.6	2.6
STYK1	0.1	0.4	0.4	2.5	2.6
SUCNR1	0.1	0.9	0.7	6.4	5.4
SULF2	0.1	0.3	0.4	2.8	4.2
SULT1A1	3.9	9.0	8.6	2.3	2.2
SULT1A2	0.3	1.0	0.8	3.0	2.4
SUMO2	229.6	87.8	78.7	0.4	0.3
SUSD3	2.4	9.8	9.8	4.0	4.1
SUSD4	0.4	0.1	0.1	0.4	0.3
SV2A	0.6	3.8	4.6	6.6	8.0
SVIL	0.1	0.4	0.4	2.9	2.7
SYBU	0.1	0.3	0.3	2.5	2.5
SYCP1	0.1	0.0	0.0	#NAME?	#NAME?
SYCP2	0.0	0.3	0.1	6.9	3.5
SYNE4	0.2	0.1	0.1	0.4	0.4
SYNGR3	0.2	0.3	0.6	2.1	4.0
SYNGR4	0.0	0.2	0.1	4.7	2.0
SYT11	0.6	2.7	2.5	5.0	4.5
SYT5	0.0	0.1	0.1	8.3	5.2
TAC3	1.2	2.9	2.7	2.4	2.2
TACC2	1.9	4.4	3.9	2.3	2.0
TACR2	0.0	0.1	0.2	3.5	4.2
TAGAP	0.5	1.1	1.3	2.3	3.0
TANGO2	64.0	29.8	31.8	0.5	0.5
TAS2R19	0.0	0.4	0.4	9.3	9.2
TAS2R30	0.0	0.2	0.2	6.0	4.1
TAS2R42	0.0	0.1	0.0	Inf	Inf
TBC1D19	0.6	1.7	1.2	2.9	2.1
TBC1D8B	0.3	0.6	0.9	2.0	3.0
TBKBP1	0.2	0.8	0.7	3.8	3.4
TBXA2R	0.4	0.9	0.8	2.7	2.2
TBXAS1	3.9	11.9	13.3	3.0	3.4
TC2N	0.3	0.6	0.7	2.1	2.5
TCEA2	0.5	1.6	1.1	3.4	2.2
TCEAL3	5.4	11.6	11.1	2.1	2.0
TCF15	0.1	0.9	0.7	6.9	5.4

TCF7	0.1	0.2	0.2	2.1	2.4
TCTE3	0.3	0.1	0.1	0.4	0.3
TCTEX1D1	0.3	1.1	0.7	3.8	2.7
TDRD10	0.2	0.0	0.0	0.0	#NAME?
TDRD9	0.1	0.7	0.5	5.4	3.9
TEAD1	1.1	0.2	0.1	0.2	0.1
TEAD4	0.3	1.8	2.1	5.2	6.3
TEK	0.0	0.3	0.3	7.9	8.3
TERC	1.2	0.2	0.4	0.2	0.3
TERT	0.0	0.1	0.1	2.9	4.3
TET1	0.3	0.8	0.7	2.5	2.1
TET3	17.7	6.4	6.7	0.4	0.4
TEX15	0.0	0.4	0.4	9.1	8.2
TFEC	0.0	0.2	0.1	5.7	3.5
TGFA	0.0	0.1	0.1	3.5	2.1
TGFBI	0.0	0.2	0.1	Inf	Inf
TGM2	27.1	10.0	10.1	0.4	0.4
THBS1	22.1	69.7	72.6	3.2	3.3
THRB-AS1	0.2	0.4	0.5	2.2	3.1
THSD7A	0.0	0.1	0.1	30.2	38.1
TIAM2	0.0	0.1	0.1	6.8	3.4
TIE1	0.1	0.5	0.4	6.6	6.4
TIMP2	0.0	0.3	0.3	7.0	5.3
TINAGL1	0.2	0.0	0.1	#NAME?	0.4
TINCR	1.2	0.5	0.5	0.4	0.4
TJP1	0.1	0.3	0.1	5.5	2.6
TK1	111.4	46.3	50.6	0.4	0.5
TKTL1	0.1	0.5	0.3	7.6	4.7
TLE2	0.2	0.1	0.1	0.3	0.5
TLR2	0.0	0.2	0.1	7.7	4.6
TLR4	0.1	1.0	0.6	8.0	5.0
TLX3	0.2	0.0	0.1	0.1	0.4
TM4SF19-TC	0.0	0.2	0.3	################	################
TM6SF1	2.9	7.6	6.5	2.6	2.2
TMC5	1.0	0.4	0.3	0.4	0.3
TMC7	0.1	0.1	0.0	0.5	0.4
TMCC2	7.1	1.9	2.3	0.3	0.3
TMEM110-M	0.0	0.3	0.9	21420.2	64835.8
TMEM136	0.0	0.3	0.3	6.7	7.4
TMEM14E	0.1	0.2	0.2	4.8	4.7
TMEM150B	0.0	0.4	0.1	10.0	2.0
TMEM154	0.5	1.6	1.3	3.2	2.7
TMEM156	0.1	0.3	0.5	3.9	6.3
TMEM158	0.2	1.2	0.8	5.9	4.1
TMEM163	0.1	0.9	1.0	8.7	10.4
TMEM169	0.0	0.1	0.2	2.1	5.0
TMEM170B	0.6	1.3	1.3	2.2	2.2

TMEM176B	0.0	0.1	0.1	Inf	Inf
TMEM189-U	0.0	0.0	0.5	485619.6	################
TMEM191C	0.0	0.1	0.2	Inf	Inf
TMEM198	0.1	0.4	0.6	2.7	4.1
TMEM204	1.4	4.4	4.4	3.3	3.2
TMEM220	1.2	2.6	2.7	2.2	2.3
TMEM229B	0.8	2.2	2.8	2.9	3.6
TMEM232	0.0	0.1	0.2	4.5	9.5
TMEM233	0.7	0.0	0.0	#NAME?	0.1
TMEM238	0.7	2.4	1.9	3.3	2.7
TMEM249	0.2	0.5	0.6	2.2	2.3
TMEM253	0.3	0.7	0.8	2.3	2.6
TMEM255B	0.0	0.2	0.3	4.7	8.2
TMEM25	0.1	0.6	0.6	5.9	5.9
TMEM45A	0.4	1.1	0.8	2.5	2.0
TMEM51	0.3	0.7	0.7	2.5	2.8
TMEM63C	0.8	1.7	1.7	2.1	2.1
TMEM71	0.1	0.2	0.2	2.4	3.2
TMEM74B	0.1	0.5	0.3	5.2	3.5
TMEM98	13.8	5.8	6.7	0.4	0.5
TMOD2	0.4	0.1	0.1	0.4	0.2
TMX2	59.9	28.0	27.9	0.5	0.5
TNFAIP2	0.2	0.9	0.9	3.9	4.2
TNFAIP6	0.2	0.4	0.5	2.3	3.0
TNFAIP8L2-S	0.6	1.2	1.6	2.1	2.8
TNFAIP8L2	2.3	6.6	6.5	2.8	2.8
TNFRSF11A	0.2	0.7	0.6	4.1	3.6
TNFRSF19	10.6	4.6	3.6	0.4	0.3
TNFRSF21	1.4	4.4	5.2	3.2	3.8
TNFRSF25	0.4	2.3	2.3	5.2	5.1
TNFRSF8	0.1	1.1	1.2	14.3	15.5
TNFSF13B	0.9	4.6	4.0	5.1	4.4
TNNI3	1.8	5.4	5.1	3.0	2.8
TNNT1	0.3	1.1	1.9	3.9	6.5
TOP1P1	0.1	0.2	0.3	2.5	4.2
TP53AIP1	0.0	0.2	0.1	Inf	Inf
TP53I11	0.4	5.8	6.5	14.1	15.7
TP53TG5	0.3	0.1	0.0	0.4	#NAME?
TP73	0.3	0.1	0.0	0.4	0.1
TPD52	1.9	6.7	5.8	3.6	3.1
TPH1	0.1	0.4	0.2	4.1	2.1
TPM2	0.6	1.5	2.0	2.4	3.3
TPM4	29.2	78.8	68.1	2.7	2.3
TPR	9.8	28.3	27.8	2.9	2.8
TPST1	0.2	0.5	0.4	3.2	2.3
TPTE2P6	0.1	0.4	0.4	3.5	3.3
TPTE2	0.0	0.1	0.2	3.6	8.8

TRABD2A	0.1	0.0	0.0	0.2	0.1
TRAF3IP3	1.2	3.0	2.8	2.5	2.4
TRAK2	32.5	12.0	11.3	0.4	0.3
TREML2	1.5	4.4	4.3	2.9	2.9
TREML3P	0.1	0.6	0.4	9.8	7.2
TRERF1	0.3	1.7	1.7	5.1	5.1
TRHR	0.0	0.2	0.1	Inf	Inf
TRIB3	21.6	3.2	2.8	0.1	0.1
TRIM10	14.7	3.2	3.7	0.2	0.2
TRIM16L	1.1	3.4	3.0	3.0	2.6
TRIM16	1.1	3.1	3.1	3.0	3.0
TRIM17	1.5	3.2	3.2	2.1	2.1
TRIM2	0.0	0.1	0.2	8.1	12.7
TRIM46	0.2	1.8	1.6	9.0	7.9
TRIM63	0.1	0.4	0.3	3.0	2.2
TRIM9	0.0	0.1	0.1	2.6	3.7
TRIO	0.9	2.1	2.1	2.3	2.3
TRIP11	2.0	5.3	5.2	2.6	2.6
TRO	0.1	0.6	0.4	10.0	6.9
TRPC1	0.1	0.9	0.6	6.5	4.3
TRPC2	0.2	0.6	0.7	3.5	4.0
TRPV3	0.0	0.2	0.1	4.9	2.4
TRPV5	0.2	0.0	0.1	0.2	0.3
TSC22D1-AS1	0.2	0.7	0.7	3.6	3.6
TSGA10	0.6	1.3	1.2	2.2	2.0
TSPAN10	0.5	0.1	0.2	0.2	0.4
TSPAN12	0.0	0.2	0.3	13.9	21.0
TSPAN2	0.1	0.3	0.3	5.8	4.9
TSPAN33	1.7	3.7	4.5	2.2	2.7
TSPAN9	0.7	1.5	2.0	2.2	2.8
TSPO2	16.1	2.8	1.8	0.2	0.1
TSPYL5	0.1	0.7	0.7	4.9	4.9
TSSK2	0.3	0.6	0.8	2.2	2.9
TTC29	1.1	0.4	0.4	0.4	0.4
TTC39A	0.3	0.1	0.1	0.4	0.2
TTC39C	0.9	3.7	3.7	4.3	4.2
TTC3P1	0.0	0.1	0.1	4.2	9.4
TTLL7	0.3	1.3	0.7	3.6	2.0
TTTY10	0.0	0.1	0.2	3.1	5.7
TTYH3	2.7	7.0	7.9	2.6	3.0
TUBA1A	14.1	67.7	60.6	4.8	4.3
TUBB2B	1.5	5.4	5.8	3.5	3.8
TUBB4A	0.2	0.9	1.1	5.2	6.3
TUBG1	68.6	31.0	30.6	0.5	0.4
TUBG2	14.7	5.7	5.4	0.4	0.4
TVP23B	4.1	9.4	8.5	2.3	2.1
TXK	0.4	1.1	1.1	2.7	2.8

TXLNB	0.0	0.2	0.1	27.2	20.0
TXNDC2	0.0	0.3	0.2	9.0	5.2
TYROBP	1.9	6.6	6.5	3.5	3.5
UACA	0.1	0.5	0.5	4.6	4.0
UBAC1	204.7	85.6	89.7	0.4	0.4
UBASH3B	2.8	7.3	6.4	2.6	2.3
UBE2F-SCLY	1.1	0.2	0.4	0.2	0.3
UBE2MP1	0.4	1.3	1.2	3.0	2.8
UCHL1	0.1	0.8	0.5	12.5	8.7
UCN2	0.0	0.3	0.2	14.3	9.3
UGDH-AS1	9.2	24.0	21.7	2.6	2.4
UGT2B28	0.0	0.2	0.1	10.2	3.1
ULK4P3	0.0	0.2	0.3	6.8	10.3
UMODL1	0.0	0.7	0.5	23.8	15.0
UNC119	2.0	4.6	5.3	2.3	2.6
UPK1A-AS1	1.0	2.3	2.6	2.3	2.5
UPK1A	0.1	0.1	0.5	2.0	9.3
UPP1	0.1	0.1	0.1	2.1	2.1
USP27X	0.6	1.6	1.2	2.6	2.0
USP2	0.0	0.1	0.1	2.4	5.9
USP51	0.4	1.0	0.9	2.3	2.1
USP53	0.7	1.4	1.4	2.0	2.1
USP9Y	0.2	0.6	0.5	2.7	2.4
UTRN	0.5	2.3	2.2	4.8	4.5
UTS2	0.2	1.3	1.3	5.7	5.9
VANGL2	0.3	0.7	0.8	2.3	2.4
VASN	0.2	0.6	0.5	2.9	2.2
VAV2	1.4	4.2	4.9	2.9	3.4
VAV3	2.5	6.8	5.5	2.7	2.2
VEGFC	0.1	0.6	0.3	4.7	2.6
VEPH1	0.0	0.2	0.1	15.7	5.3
VIP	0.0	0.1	0.0	Inf	Inf
VNN1	0.0	0.2	0.1	11.1	5.3
VNN2	0.4	1.0	0.8	2.5	2.0
VPS13A	5.3	14.7	15.4	2.8	2.9
VPS13C	2.8	7.3	7.2	2.6	2.6
VSIG10L	0.5	1.0	1.2	2.2	2.6
VSIG10	0.1	0.4	0.4	2.9	3.1
VSIG2	0.1	0.2	0.5	2.3	5.5
VSIG4	0.0	0.1	0.0	Inf	Inf
VTN	0.1	0.1	0.1	2.1	2.4
VWA2	0.1	0.2	0.2	4.1	3.9
VWA7	0.3	0.9	1.2	3.5	4.2
VWDE	0.6	1.5	1.4	2.3	2.1
VWF	0.8	2.5	2.8	3.2	3.6
WASF3	0.2	0.6	0.4	3.8	2.7
WAS	4.1	10.5	12.7	2.5	3.1

WBP5	11.6	36.6	28.0	3.2	2.4
WDFY4	0.0	0.1	0.1	6.9	5.3
WDR54	1.2	8.3	6.3	7.0	5.3
WFDC1	0.4	1.3	1.2	3.5	3.2
WIPF1	4.1	13.3	13.0	3.2	3.2
WNK2	0.0	0.1	0.1	5.5	6.8
WNK3	0.1	0.0	0.0	0.3	0.2
WWTR1	0.1	0.2	0.2	2.4	2.9
XIRP1	0.3	2.2	3.1	8.7	12.2
XIRP2	0.0	0.2	0.2	6.0	4.9
XKR9	0.2	0.7	0.6	3.3	2.6
XYLT1	0.1	0.7	0.6	8.0	7.4
ZBTB41	0.4	1.6	1.3	4.3	3.6
ZBTB46	0.2	0.6	0.5	2.6	2.0
ZBTB47	0.0	0.2	0.2	12.1	14.8
ZC3H12C	0.2	1.3	0.9	5.4	3.5
ZDHHC15	0.0	0.4	0.7	7.2	13.4
ZDHHC19	0.1	0.2	0.2	2.0	3.1
ZDHHC21	1.4	3.6	3.3	2.5	2.3
ZDHHC23	0.5	2.1	2.1	4.1	4.3
ZFC3H1	5.8	11.9	12.5	2.0	2.1
ZFHX2	0.0	0.1	0.1	2.9	5.6
ZFP28	0.2	0.5	0.7	2.6	4.0
ZFP36	40.9	14.7	15.6	0.4	0.4
ZFPM2	0.0	0.2	0.3	6.0	8.9
ZFX-AS1	0.1	0.0	0.0	#NAME?	#NAME?
ZG16B	0.2	0.7	0.5	3.1	2.2
ZMAT1	0.3	2.0	1.4	7.8	5.5
ZNF100	1.8	4.2	4.4	2.3	2.4
ZNF107	1.5	3.8	3.9	2.6	2.6
ZNF117	0.7	4.2	3.4	5.9	4.8
ZNF135	0.0	0.2	0.2	8.1	8.3
ZNF177	0.1	0.6	0.7	5.2	6.1
ZNF214	0.1	0.2	0.2	2.7	2.2
ZNF260	2.0	4.4	4.2	2.2	2.1
ZNF286A	0.5	1.1	1.4	2.3	2.7
ZNF287	0.1	0.4	0.5	2.6	3.4
ZNF28	1.5	3.3	3.1	2.1	2.1
ZNF292	2.1	6.1	6.0	2.8	2.8
ZNF32	1.5	4.9	3.5	3.2	2.3
ZNF334	0.2	0.7	0.7	3.3	3.3
ZNF33A	3.6	9.3	8.8	2.6	2.5
ZNF33B	2.5	5.8	5.7	2.3	2.2
ZNF354C	0.2	0.5	0.5	3.1	3.1
ZNF37A	2.5	5.3	5.0	2.1	2.0
ZNF385A	2.6	7.3	8.7	2.8	3.3
ZNF439	1.1	2.7	2.7	2.5	2.5

ZNF43	0.7	1.8	1.9	2.7	2.9
ZNF462	0.0	0.5	0.7	13.7	17.4
ZNF467	0.1	0.2	0.2	2.5	3.1
ZNF469	0.0	0.1	0.1	6.9	6.8
ZNF470	0.2	0.8	0.7	3.5	3.2
ZNF471	0.3	0.7	0.8	2.6	2.9
ZNF480	2.5	6.4	6.9	2.5	2.8
ZNF485	0.4	0.8	0.8	2.0	2.1
ZNF518A	2.3	5.5	4.8	2.4	2.1
ZNF528	1.2	2.4	2.4	2.1	2.0
ZNF546	0.4	0.9	0.9	2.1	2.3
ZNF596	0.4	1.0	1.0	2.3	2.5
ZNF606	0.7	1.4	1.6	2.1	2.3
ZNF625-ZNF2	0.2	0.3	0.4	2.0	2.6
ZNF658	0.1	0.2	0.2	2.2	3.0
ZNF667	0.5	1.5	1.5	3.3	3.2
ZNF677	0.3	0.6	0.7	2.1	2.3
ZNF678	0.9	2.2	2.1	2.3	2.3
ZNF681	0.1	0.5	0.3	4.5	2.5
ZNF682	0.0	0.3	0.2	7.8	4.5
ZNF711	2.3	8.3	7.2	3.6	3.1
ZNF721	2.4	6.2	5.3	2.6	2.2
ZNF804A	0.2	2.2	1.5	13.9	9.7
ZNF804B	0.0	0.0	0.1	6.3	17.9
ZNF876P	0.1	0.5	0.5	3.7	3.6
ZNF880	1.4	3.7	3.8	2.6	2.7
ZNF91	0.5	1.6	1.7	3.2	3.5
ZNRF2P2	0.1	0.0	0.0	0.3	0.3
ZRANB2-AS1	0.5	1.2	1.3	2.6	2.9
